# ﻿Description of immature stages of *Rhinusa* species (Coleoptera, Curculionidae, Mecinini) with a focus on diagnostic morphological characters at the species and genus levels

**DOI:** 10.3897/zookeys.1195.112328

**Published:** 2024-03-14

**Authors:** Rafał Gosik, Roberto Caldara, Ivo Toševski, Jiří Skuhrovec

**Affiliations:** 1 Department of Zoology and Nature Protection, Faculty of Biology and Biotechnology, Maria Curie-Skłodowska University, Akademicka 19, 20-033 Lublin, Poland Maria Curie-Skłodowska University Lublin Poland; 2 Via Lorenteggio 37, 20146 Milan, Italy Unaffiliated Milan Italy; 3 CABI, Rue des Grillons 1, 2800 Delémont, Switzerland CABI Delémont Switzerland; 4 Institute for Plant Protection and Environment, Banatska 33, 11080 Zemun, Serbia Institute for Plant Protection and Environment Zemun Serbia; 5 Group Function of Invertebrate and Plant Biodiversity in Agro-Ecosystems, Crop Research Institute, Prague 6–Ruzyně, Czech Republic Crop Research Institute Prague Czech Republic

**Keywords:** Biology, mature larva, Mecinini, morphology, pupa, taxonomy

## Abstract

The mature larvae of the following fourteen *Rhinusa* species are described and illustrated: *Rhinusaantirrhini* (Paykull, 1800), *R.asellus* (Gravenhorst, 1807), *R.collina* (Gyllenhal, 1813), *R.eversmanni* (Rosenschoeld, 1838), *R.florum* (Rubsaamen, 1895), *R.herbarum* (H. Brisout de Barneville, 1862), *R.incana* (Kirsch, 1881), *R.linariae* (Panzer, 1796), *R.melas* (Boheman, 1838), *R.neta* (Germar, 1821), *R.pilosa* (Gyllenhal, 1838), *R.rara* Toševski & Caldara, 2015, *R.tetra* (Fabricius, 1792), and *R.vestita* (Germar, 1821). The pupae of thirteen of them (except *R.incana*) were also described. The comparison of larval morphological characters and plant preferences provides evidence supporting the existence of different species groups previously established according to a phylogenetic analysis based on adult morphological characters. The following diagnostic attributes distinguishing the genus *Rhinusa* are highlighted. For the larvae: (1) pronotal shield indistinct; (2) thoracic prodorsal fold small or even vestigial; (3) abdominal postdorsal folds (especially of segments III–VII) high or even in the form of conical protuberances; (4) cuticle of abdominal segments densely covered with asperities; (5) cuticle without dark spots or dark pigmentation; (6) head suboval, rarely round; (7) labrum usually with 2 *als*; (8) *des_1_* short or absent, rarely elongated; and (9) *fs_1_*_-3_ usually absent or minute. For the pupae: (1) body stout; (2) head protuberances always present; (3) pronotal protuberances (if present), separated at bases of the pronotum, always wider than higher; (4) abdominal protuberance usually present, wide or round; (5) femora usually with a single *fes*; and (6) urogomphi short or vestigial. Keys to the larvae and pupae described here are provided. All the characters used for identification are illustrated by photographs or drawings. Biological and distribution data, including new information, are provided for all the species studied.

## ﻿Introduction

The weevil genus *Rhinusa* Stephens, 1829, is a member of the tribe Mecinini (Curculionidae, Curculioninae) and is currently composed of 52 valid species with a Palearctic distribution (Reitter 1908; Caldara 2001; Caldara et al. 2010; Alonso-Zarazaga et al. 2023). Whereas two groups – the *R.tetra* group and the *R.bipustulata* group – live on *Verbascum* L. and *Scrophularia* L. in the plant family Scrophulariaceae (Caldara 2014), the other groups live on species of Plantaginaceae as presently circumscribed (APG 2016), which belong to the genera *Antirrhinum* L., *Chaenorhinum* (D.C.) Rchb, *Kickxia* Dumort., *Linaria* Miller, and *Misopates* Raf., all being either monophagous or oligophagous because living on one or several plant species of the same genus (Hernández-Vera et al. 2010; Caldara 2014; Caldara and Toševski 2019; Toševski et al. 2023). The larvae develop inside the ovaries, stems, or roots of their host plants and sometimes induce galls (Hoffmann 1958; Caldara 2001). Interestingly, several of them are inquilines in galls produced by other species of the same genus (Hoffmann 1958; Arzanov 2000; Caldara 2001, 2003, 2005, 2007; Korotyaev et al. 2005). The genus contains several species that have been the subject of detailed ecological studies (Smith 1959; Groppe 1992; Jordan 1994; Gassmann and Paetel 1998; Toševski and Gassmann 2004), because of their value as potential biological control agents of several species of toadflax (*Linaria*), which were introduced into North America and became invasive (Saner et al. 1995; Vujnovic and Wein 1997).

To date, larvae of approximately 45 Mecinini species have been described (Gardner 1934; van Emden 1938; Scherf 1964; Anderson 1973; Lee and Morimoto 1988; May 1994; Gosik 2010; Jiang and Zhang 2015; Ścibior and Łętowski 2018; Skuhrovec et al. 2018; Gosik et al. 2020; Skuhrovec et al. 2022), while descriptions of pupae are known for 33 Mecinini species (Scherf 1964; Anderson 1973; Gosik 2010; Jiang and Zhang 2015; Skuhrovec et al. 2018; Gosik et al. 2020; Skuhrovec et al. 2022). Unfortunately, the use of some of these descriptions for comparison is somewhat problematic due to missing data on the chaetotaxy and/or the absence of drawings with enough detail.

The taxonomic classification of species within *Rhinusa* and of the whole tribe Mecinini has proven difficult, and it is still the subject of extensive studies (Caldara 2008; Caldara and Fogato 2013; Caldara et al. 2013; Gosik et al. 2020). The previous arrangement of *Rhinusa* into four species groups as well as its treatment as a subgenus of *Gymnetron*, as proposed by Reitter (1908) based on few adult morphological characters, were generally accepted (Hustache 1931; Hoffmann 1958; Smreczyński 1976; Lohse and Tischler 1983). However, a more recent taxonomic treatment based on a comprehensive morphological study suggests that *Rhinusa* is monophyletic and sister group of *Gymnetron* Schoenherr, 1825 (Caldara 2001; Caldara et al. 2010). The immature stages are a source of additional characters that may help solve taxonomic problems at species, genus and tribal level for this important group of weevils.

Therefore, the aims of the present study are to describe the 14 *Rhinusa* species in complete detail (with larvae and pupae) for the first time, to identify characters that are diagnostic at the genus and species levels, and finally, to compare the characters of the immature stages of this genus with those of other genera of Mecinini.

## ﻿Materials and methods

### ﻿Insect collection

Mature larvae (those of the last, third, instar, L3) and pupae of each of the studied species were obtained by collecting them from the host plants on which the adults were observed or by rearing them from the galls or seed capsules of those same host plants. Some larvae were preserved for rearing pupae and these were in turn used to obtain adults in order to be sure about the identity of the species. These specimens were then preserved in 2 ml screw-cap microtubes (Sarstedt, Germany) that were half-filled with 96% ethanol and kept at 4–6 °C. RC and IT were responsible for classifying the insect and plant taxa, respectively. For the morphological descriptions, some of the larval and pupal material was employed. These specimens have been added to the collection of Maria Curie-Skłodowska University’s Department of Zoology and Nature Protection in Lublin, Poland. The Results section includes information about the numbers of specimens obtained and their dates and localities of collection.

The larvae and pupae of the majority of the studied species (*R.collina* (Gyllenhal, 1813), *R.eversmanni* (Rosenschoeld, 1838), *R.florum* (Rubsaamen, 1895), *R.herbarum* (H. Brisout de Barneville, 1862), *R.incana* (Kirsch, 1881), *R.linariae* (Panzer, 1796), *R.melas* (Boheman, 1838), *R.pilosa* (Gyllenhal, 1838), *R.rara* Toševski & Caldara, 2015, and *R.vestita* (Germar, 1821)) are described and illustrated for the first time. Detailed redescriptions are provided for those of *R.asellus* (Gravenhorst, 1807), *R.antirrhini* (Paykull, 1800), *R.neta* (Germar, 1821), and *R.tetra* (Fabricius, 1792), updating and enlarging the information given in previous papers (van Emden 1938; Scherf 1964; Anderson 1973; Ścibior and Łętowski 2018).

### ﻿Morphological descriptions

The preparation of the slide-mounted material basically followed May (1994). The larvae chosen for microscopic examination were first dissected (the head, mouthparts, and body were separated), then cleared in 10% potassium hydroxide (KOH), rinsed in distilled water, and mounted on permanent microscope slides in Faure–Berlese fluid (50 g of gum arabic and 45 g of chloral hydrate dissolved in 80 g of distilled water and 60 cm^3^ of glycerol; Hille Ris Lambers 1950). All of the mentioned specimens were preserved in 95% ethanol and examined using calibrated oculars and an optical stereomicroscope (Olympus SZ 60 and Nikon Eclipse 80i). Using Corel Photo-Paint X7 and Corel Draw X7, drawings and outlines were created using a drawing tube (MNR-1) mounted on a stereomicroscope (Ampliwal). The larval instars’ body length (**BL**) , body width (**BW**) at the third abdominal segment , and head capsule width (**HW**) were all measured (see Gosik et al. 2016, or Skuhrovec and Bogusch 2016). Pupae were measured for body length (**BL**), body width (**BW**) at the level of the midlegs, rostrum length (**RL**), and pronotum width (**PW**) (see Gosik and Skuhrovec 2011). Marvaldi (1997, 1999, 2003) and Oberprieler et al. (2014) were followed for terminology on chaetotaxy and body parts, and Zacharuk (1985) for terms concerning the antennae.

## ﻿Results

### ﻿ Rhinusa

Taxon classificationAnimaliaColeopteraCurculionidae

Stephens, 1829

9B0EA9BB-EE8A-54A8-92B9-B3ABCA293F3E

#### Description of mature larva (L3).

***Measurements*** (in mm). Body length: 2.00 (*R.florum*) – 9.00 (*R.asellus*). The widest point of the body (metathorax) measures up to 2.35 (*R.vestita*). Head width: 0.46 (*R.florum*) – 1.05 (*R.vestita*).

***General*.** Body elongate, slender, curved, and usually rounded in cross section. All thoracic segments almost equal in size, or pronotum smaller than the next segments. Meso- and metathorax each divided dorsally into two folds, the prodorsum distinctly smaller than postdorsum or even vestigial. The pedal fold of thoracic segments very distinct, usually conical, and prominent. Abdominal segments I–VI of similar size, next segments tapering towards the posterior body end. Abdominal segments I–VII each divided dorsally into two transverse folds: prodorsum slightly smaller than postdorsum; postdorsum usually higher than prodorsum or in the form of conical protuberances; seldom both folds equally raised. Segments VIII and IX dorsally undivided. Epipleural fold of segments I–VIII conical. Laterosternal and eusternal folds of segments I–VIII conical, usually weakly distinct. Thoracic and abdominal cuticle densely covered with fine, unicoloured cuticular asperities. Abdominal segment X divided into four folds of equal size; almost completely hidden by the previous segment. Anus situated ventrally.

Thoracic spiracles often unicameral, but sometimes bicameral (*R.antirrhini*, *R.florum*, and *R.melas*), abdominal spiracles always unicameral; thoracic spiracles placed laterally on prothorax, close to mesothorax; abdominal spiracles placed antero-laterally or antero-medially on segments I–VIII.

***Colouration*.** Head capsule light yellow to dark brown, medial parts of epicranium usually less sclerotised. All thoracic and abdominal segments whitish or light yellow. Pronotal sclerite indistinct, not more pigmented than the rest of the segment (only in *R.eversmanni* and *R.neta* slightly more pigmented than the rest of the segment).

***Vestiture*.** Setae on body thin, yellowish, different in length (very short or medium), transparent or brownish.

***Head capsule*.** Head suboval or slightly narrowed bilaterally, endocarinal line present, reaching from 1/2 to 4/5 of the length of frons. Frontal sutures usually very wide, hardly or weakly distinct. Frons covered with knobby asperities (*R.collina*, *R.eversmanni*, *R.incana*, and *R.neta*) or smooth. Usually only one single pair of anterior stemmata present, in the form of small black spots (st) close to the end of the frontal suture, two pairs of stemmata present in *R.asellus*, *R.collina*, *R.incana*, and *R.linariae*. *Des_1_* usually short or absent; *des_2_* usually elongated, located on the lateral part of the epicranium; long *des_3_* located anteriorly on the epicranium on the border of the frontal suture; *des_4_* minute or absent; and *des_5_* long, located anterolaterally. *Fs_1-3_* minute or absent, located medially; *fs_4_* long, located anteriorly; and long *fs_5_* located anterolaterally, close to the antenna. *Les_1_* and *les_2_* medium to short; single *ves* short or absent. One to five minute postepicranial setae (*pes*).

***Antennae*** placed distally of the frontal suture, on the inside. Membranous and distinctly convex basal article bearing one conical sensorium plus some smaller sensilla: ampullacea, basiconica, or styloconica.

***Clypeus*** trapezoidal, usually with two *cls* short to relatively elongated; sometimes basal part much more sclerotised than the apical parts; anterior border more or less curved towards the inside.

***Mouth parts*.** Labrum usually distinct from clypeus by clypeo-labral suture (in *R.pilosa*, *R.rara*, and *R.linariae* clypeus and labrum fused) with three piliform *lrs* (only in *R.linariae* single *lrs*), usually *lrs_1_* and *lrs_2_* elongated, located medially, and *lrs_3_* short, located laterally; anterior border of labrum bi-sinuate or slightly rounded. Epipharynx mostly with two (rarely three) relatively elongated, finger-like *als* (almost identical in length); two or three piliform *ams* varying in size; without or with up to two short, finger-like *mes*; labral rods (lr) usually prominent, elongated, kidney-shaped, or rounded, almost indistinct. Mandibles apically bifid, cutting edge with additional protuberance or smooth; two medium-sized piliform *mds*, both located close to the lateral border. Maxillolabial complex: maxilla usually more sclerotised than labium, stipes with one *stps*, two *pfs*, and one *mbs*; *stps* and both *pfs_1–2_* short to elongated; mala with four to six finger-like *dms* variable in length; from two up to four piliform *vms*, medium to short in length. Maxillary palpi two-segmented; basal palpomere usually distinctly wider than distal one; length ratio of the basal and distal palpomeres usually almost 1:1; basal palpomere with short *mpxs* and two sensilla, distal palpomere with a group of two to six apical sensilla in the terminal receptive area. Prementum close to oval-shaped, with one medium *prms* (only *R.herbarum* with two *prms*); ligula with round or sinuate margin and one to three *ligs*; premental sclerite sclerotised in cup or ring form, sometimes incomplete, only in *R.linariae* indistinguishable, posterior extension absent or elongated; anterior median extension absent. Labial palpi one-segmented (in *R.linariae* labial palpi vestigial and almost invisible); each palp with a single pore, and a group of one to four apical sensilla (ampullacea) in the terminal receptive area; the surface of the labium smooth. Postmentum with two or three *pms*; membranous area smooth or partially covered with sharp or knobby asperities.

***Thorax*.** Prothorax with four to 12 *prns*; two *ps*; and usually a short, single *eus*. Mesothorax with a single minute *prs* or without; three to four *pds* (variable in length) (only in *R.linariae* one *pds* and in *R.pilosa* two *pds*); one medium *as* (only *R.rara* without); three medium to minute *ss*; one medium *eps*; one or two medium *ps*; and a single minute *eus* (sometimes absent). Chaetotaxy of metathorax almost identical to that of mesothorax. Each pedal area of thoracic segments with three to six *pda*.

***Abdomen*.** Segments I–VIII usually with one minute *prs* (sometimes absent on segment VIII, only *R.bipustulata* with two *prs*) and one to four *pds*; usually one minute and one medium *ss*; one to three *eps*; one *ps*; one minute *lsts*; and usually two minute *eus*. Abdominal segment IX without or with up to three minute *ds*; without or with up to two minute *ps*; and without or with up to two minute *sts*. Abdominal segment X mostly without seta.

#### Description of pupa.

***Measurements*** (in mm). Body length: 1.86 (*R.florum*) – 6.50 (*R.vestita*). Body width: 1.66–3.50. Thorax width: 1.00–2.00.

***Body*.** Integument white, sometimes with some parts dark and sclerotised; setae sometimes placed on pigmented spots, more or less stout, curved. Head with a pair of protuberances (h–pr) above eyes. Rostrum moderately elongated, in males usually as long as in females or only slightly shorter than in females, reaching mesocoxae (only in *R.asellus* rostrum very elongated, distinctly variable in both sexes, much longer in females). Pronotum trapezoidal. Pronotal protuberances (p–pr) separated at bases, wider than tall (conical in *R.asellus*), sometimes vestigial (*R.linariae*) or even absent (*R.herbarum*, *R.pilosa*, and *R.rara*). Meso- and metanotum similar in size. Abdominal segments I–VI almost identical in size; segment VII semicircular; segment VIII narrow, small; segment IX reduced. Abdominal protuberance (a–pr) on abdominal segment VIII usually visible, flattened or rounded, sometimes vestigial (*R.linariae*) or even absent (*R.collina*, *R.eversmanni*, *R.pilosa*, and *R.rara*). Urogomphi (ur) short, often ending in sclerotised, sharp apexes, sometimes vestigial or completely absent.

***Chaetotaxy*** well developed, setae short to elongated, transparent or brownish. Head without or with one *os*, without or with one *sos*; rostrum without or with one *pas*, without or with up to two *rs*, without or with one *es*. Pronotum with one to three *as*, without or with up to four *ls*, two to four *pls*; without or with one *ds*. Dorsal parts of meso- and metathorax with two or three setae placed medially. Apex of femora usually with a single long *fes*, with two *fes* in *R.asellus*, *R.tetra* and *R.bipustulata*. Abdominal segments I–VII with two or up to six setae dorsally (segment VIII usually with fewer setae); one or two setae laterally, and two or up to five setae ventrally. Abdominal segment IX with two or up to four setae ventrally.

##### ﻿Descriptions of the species

Species are arranged according to the species groups proposed by Caldara et al. (2010) on the basis of a morphological study of the adults. For each group, a combination of the diagnostic characters is here listed, whereas a key to all the groups is reported by Caldara and Toševski (2019).

###### ﻿*Rhinusatetra* group

**Adult diagnosis.** Protibiae and metatibiae with a premucro, which is more pronounced in female; uncus of protibiae with base placed towards middle of apex and almost as long as width of tibiae in female, protibiae in male distinctly arcuate in apical quarter, profemora very globose, ventrites 3–5 in male along midline with hairlike scales dense and ruffled.

### ﻿1) Rhinusa asellus

Taxon classificationAnimaliaColeopteraCurculionidae

(Gravenhorst, 1807)

61EA2828-2E91-5D54-B637-040BF6182F5C

#### Material examined.

16 mature larvae; 4 ♂ pupae and 1 ♀ pupa. Serbia, Pirot, 700 m a.s.l., ex *Verbascumthapsus* L., 06.03.2017, leg., det. I. Toševski.

#### Description of mature larva

**Figure 1.**
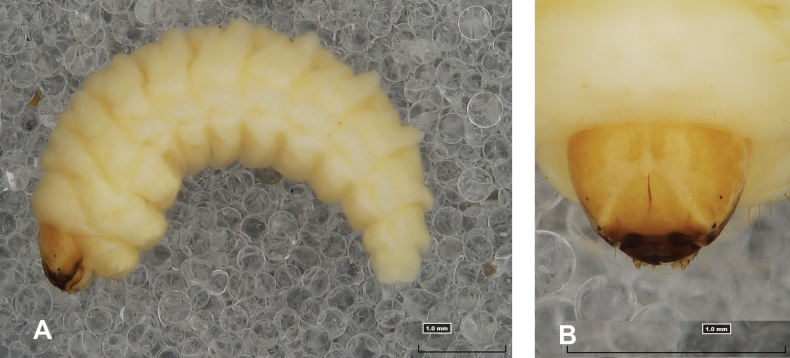
*Rhinusaasellus* (Gravenhorst, 1807) mature larva **A** habitus **B** head, frontal view.

**Figure 2.**
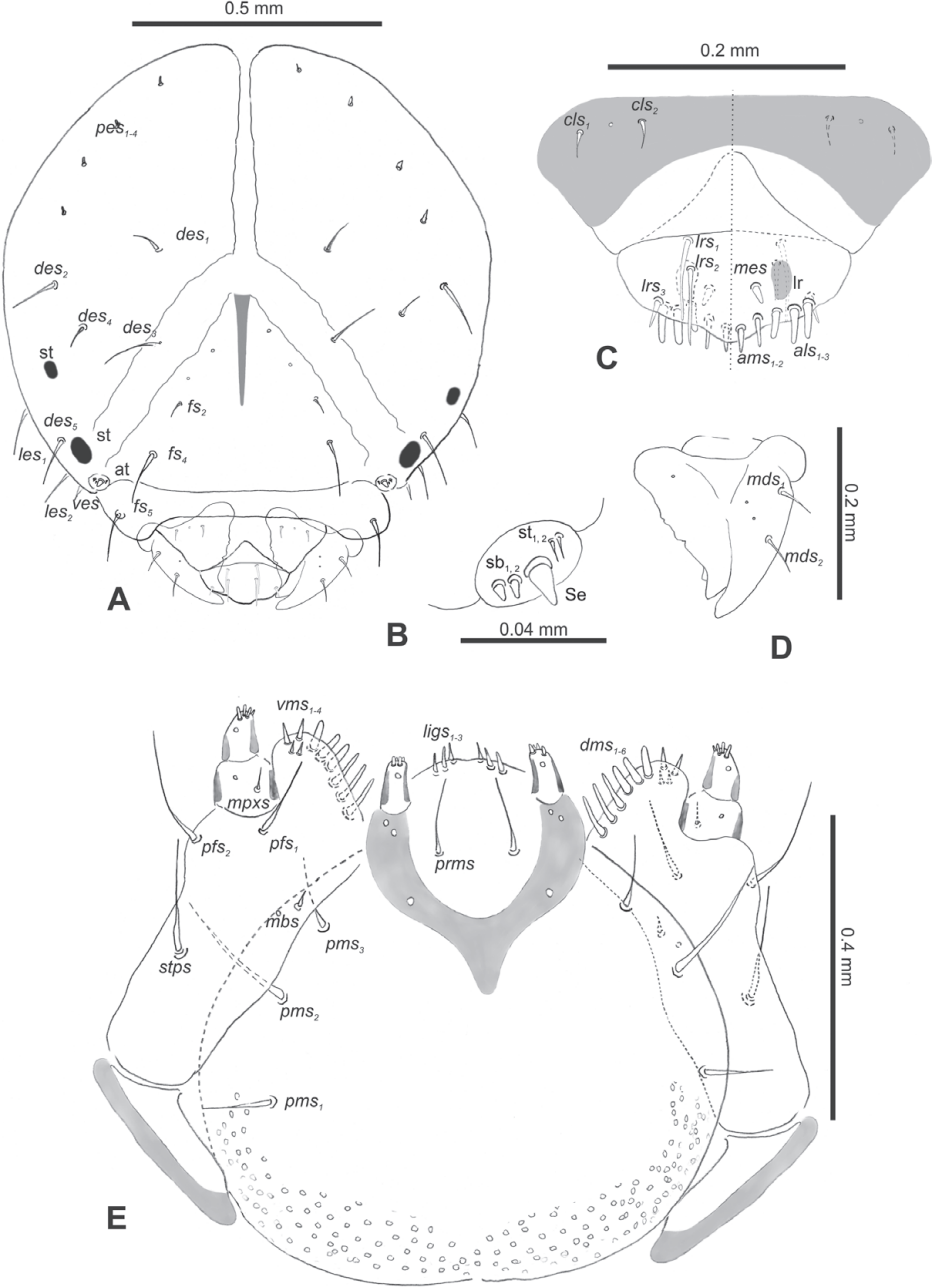
*Rhinusaasellus* (Gravenhorst, 1807) mature larva, head and mouth parts **A** head **B** antenna **C** clypeus and labrum (left side), epipharynx (right side) **D** left mandible **E** maxillolabial complex (schemes). Abbreviations: at–antenna, lr–labral rods, st–sensillum styloconicum, sb–sensillum basiconicum, Se–sensorium, st–stemmata, setae: *als*–anterolateral, *ams*–anteromedial, *cls*–clypeal, *des*–dorsal epicranial, *dms*–dorsal malar, *fs*–frontal epicranial, *les*–lateral epicranial, *ligs*–ligular, *lrs*–labral, *mbs*–malar basiventral, *mds*–mandibular dorsal, *mes*–medial, *mpxs*–maxillary palp, *pes*–postepicranial, *pfs*–palpiferal, *pms*–postmental, *prms*–premental, *stps*–stipital, *ves*–ventral, *vms*–ventral malar.

**Figure 3.**
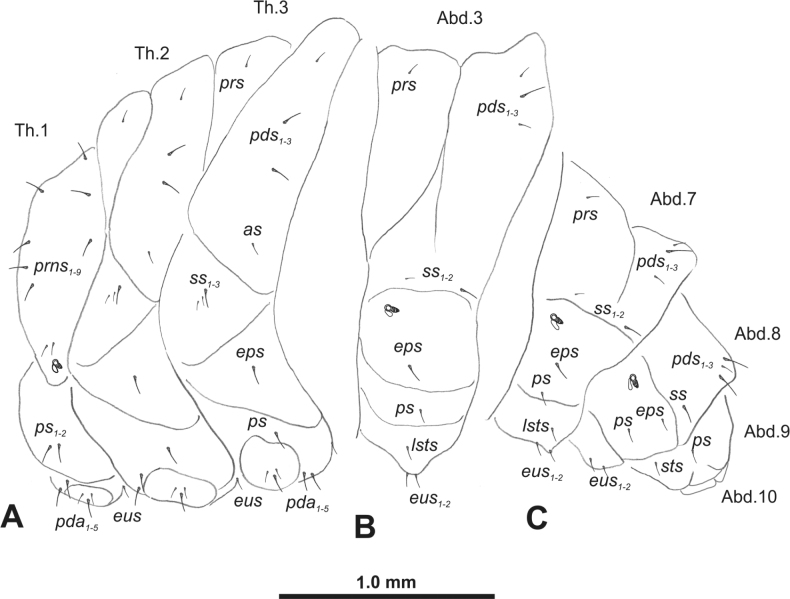
*Rhinusaasellus* (Gravenhorst, 1807) mature larva, habitus **A** lateral view of thoracic segments **B** lateral view of abdominal segment I **C** lateral view of abdominal segments VII–X (schemes). Abbreviations: Th. 1–3–number of thoracic segments, Abd. 1–10–number of abdominal seg, setae: *as*–alar, *ds*–dorsal, *eps*–epipleural, *eus*–eusternal, *lsts*–laterosternal, *pda*–pedal, *pds*–postdorsal, *prns*–pronotal, *prs*–prodorsal, *ss*–spiracular, *ps*–pleural, *sts*–sternal.

**(Figs 1A, B, 2A–E, 3A–C). *Measurements*** (in mm). Body length: 5.72–9.00 (avg. 7.55). The widest place in the body (meso- and metathorax) measures up to 2.40. Head width: 0.80–1.00 (avg. 0.85).

***General*.** Body elongate, moderately slender, curved, rounded in cross section (Fig. 1A). Prothorax small, pronotal shield not pigmented. Mesothorax slightly smaller than metathorax; each divided dorsally into two folds (prodorsal fold smaller than postdorsal fold). Pedal folds of thoracic segments isolated, conical, prominent. Abdominal segments I–III of similar size, next segments tapering towards posterior body end. Abdominal segments I–VII each divided dorsally into two folds; postdorsal folds distinctly higher than prodorsal. Segments VIII and IX dorsally undivided. Epipleural folds of segments I–VII slightly conical. Laterosternal and eusternal folds of segments I–VII conical, weakly isolated. Abdominal segment X divided into four folds of equal size. Anus situated ventrally.

All spiracles unicameral; thoracic spiracles placed laterally close to mesothorax; abdominal spiracles (Fig. 1A) placed mediolaterally on segments I–VIII.

***Colouration*.** Yellow to brownish head, medial parts of epicranium less sclerotised (Fig. 1B). All thoracic and abdominal segments white (Fig. 1A). Cuticle covered with fine asperities.

***Vestiture*.** Setae on body thin, yellowish, different in length (very short or medium).

***Head capsule*** (Figs 1B, 2A). Head rather wide, endocarinal line present, reaching to 1/2 of the length of frons. Frontal sutures on head wide, unclear. Two pairs of stemmata (st): first in the form of a prominent pigmented spot with a convex cornea, close to the end of the frontal suture, second pair small, placed laterally, above the anterior stemma. *Des_1_* short, located in the central part of epicranium; long *des_2_* placed mediolaterally; long *des_3_* located anteriorly on epicranium close to the border with the frontal suture; *des_4_* short; long *des_5_* located anterolaterally above stemma (Fig. 2A). *Fs_1_* absent; *fs_2_* short; *fs_3_* absent; *fs_4_* long, located anteriorly; and long *fs_5_* located anterolaterally, close to antenna (Fig. 2A). *Les_1_* and *les_2_* medium, and one short *ves*. Epicranial area with four *pes*.

***Antennae*** placed distally of the frontal suture, on the inside; membranous and distinctly convex basal article bearing one conical sensorium, relatively short, plus four sensilla differing in type: two basiconica and two styloconica (Fig. 2B).

***Clypeus*** (Fig. 2C) ~ 3 × as wide as long with two medium *cls*, localised posterolaterally, with one sensillum between them; basal part distinctly sclerotised; anterior border straight.

***Mouth parts*.** Labrum (Fig. 2C) trapezoidal, ~ 2.2 × as wide as long, with three piliform *lrs*, different in length; *lrs_1_* elongated, located medially, *lrs_2_* elongated, located posteromedially, and *lrs_3_* short, located anterolaterally; anterior border bi-sinuate. Epipharynx (Fig. 2C) with three medium finger-like *als*, almost identical in length; two medium piliform *ams*; and single medium finger-like *mes*; labral rods (lr) distinct, kidney shaped. Mandibles (Fig. 2D) bifid, cutting edge with additional protuberance; two medium piliform *mds*, both located in shallow pits, close to lateral border. Maxillolabial complex: maxilla (Fig. 2E) stipes with one *stps*, two *pfs* and one very short *mbs* and one sensillum, *stps* and both *pfs_1–2_* relatively long; mala with six finger-like *dms* variable in length; four piliform *vms*, medium to short in length. Maxillary palpi two-segmented; basal palpomere distinctly wider than distal one; length ratio of basal and distal palpomeres almost 1:1; basal palpomere with short *mpxs* and one sensillum, distal palpomere with a group of four apical sensilla in terminal receptive area. Prementum (Fig. 2E) oval-shaped, with one long *prms*; ligula with round margin and three short *ligs*; premental sclerite broad, sclerotised, cup-shaped, posterior extension medium in length, with thick apex. Labial palpi one-segmented; palpi with a single pore, and a group of four apical sensilla (ampullacea) on terminal receptive area; surface of labium smooth. Postmentum (Fig. 2E) with three *pms*, medium *pms_1_* located posterolaterally, elongated *pms_2_* located mediolaterally, and medium *pms_3_* located anterolaterally; posterior part of membranous area covered with knobby asperities.

***Thorax*.** Prothorax (Fig. 3A) with nine medium *prns*, dorsal sclerite weakly visible; two medium *ps*; and single short *eus*. Mesothorax (Fig. 3A) with one short *prs*, one short and two medium *pds*; one medium *as*; three minute and one medium *ss*; one medium *eps*; one medium *ps*; and single short *eus*. Chae­totaxy of metathorax (Fig. 3A) almost identical to that of mesothorax. Each pedal area of thoracic segments with three long and two short *pda*.

***Abdomen*.** Segments I–VII (Fig. 3B, C) with one very short *prs*; two short and one medium *pds*; one minute and one medium *ss*; one medium *eps*; one medium *ps*; one short *lsts*; and two short *eus*. Segment VIII (Fig. 3B, C) with two medium and one short *pds*; one medium *ss*; one medium *eps*; one medium *ps*; and two short *eus*. Abdominal segment IX (Fig. 3C) with one minute *ps* and one minute *sts*.

#### Description of pupa

**Figure 4.**
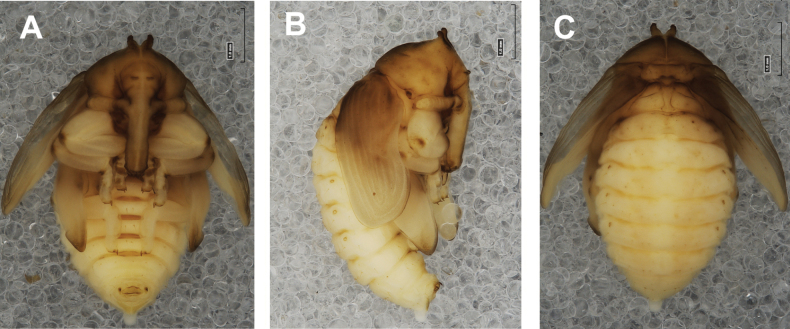
*Rhinusaasellus* (Gravenhorst, 1807) pupa habitus **A** ventral view **B** lateral view **C** dorsal view.

**Figure 5.**
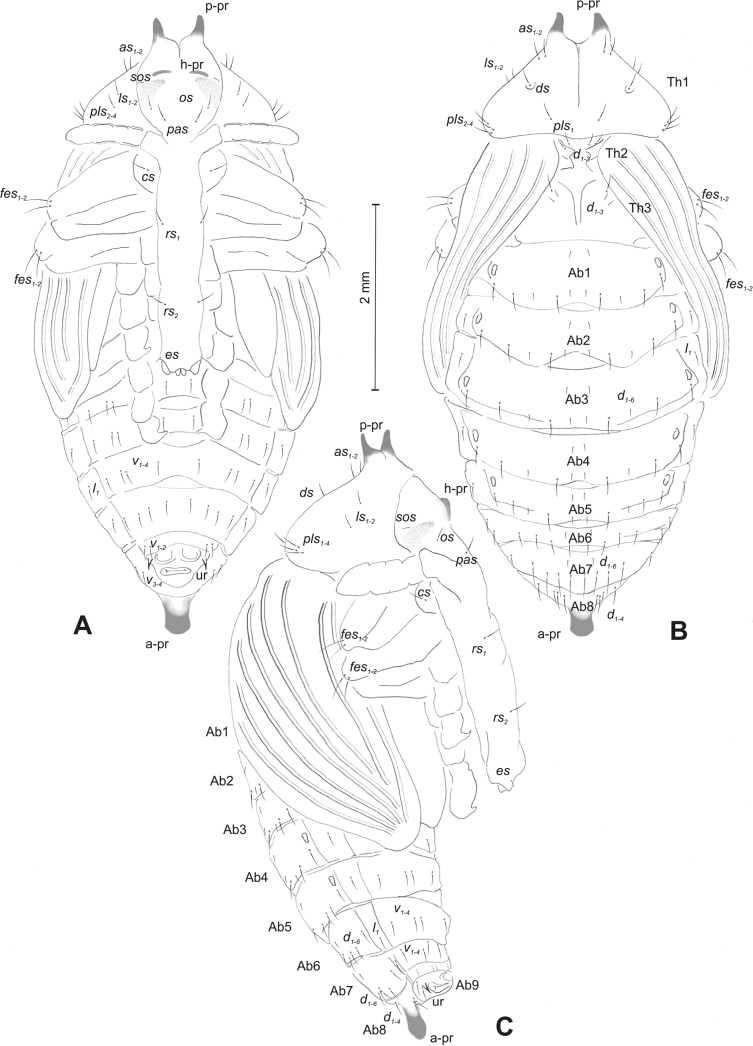
*Rhinusaasellus* (Gravenhorst, 1807) pupa habitus **A** ventral view **B** dorsal view **C** lateral view (schemes). Abbreviations: a–pr–abdominal protuberances, h–pr–head protuberances, p–pr–pronotal protuberances, ur–urogomphi, setae: *as*–apical, *cs*–coxal, *d*–dorsal, *ds*–discal, *es*–epistomal, *fes*–femoral, *l*, *ls*–lateral, *os*–orbital, *pas*–postantennal, *pls*–posterolateral, *rs*–rostral, *sos*–supraorbital, *v*–ventral.

**(Figs 4A–C, 5A–C). *Measurements*** (in mm). Body length: 4.50–6.00; body width: 2.75–3.15; thorax width: 1.75–2.90; rostrum length: up to 1.60 ♂ and 2.60 ♀.

***Body*.** Integument brownish; moderately stout, curved. Elongated head protuberances present (h–pr) on head above eyes. Rostrum very elongate, in male almost 4 × as long as wide and reaching metacoxae; in female 7 × as long as wide and protruding past metacoxae. Pronotum trapezoidal, 1.5 × as wide as long. Pronotal protuberances (p–pr) well developed, conical, sclerotised, fused at base. Mesonotum slightly smaller than metanotum. Abdominal segments I–VI almost identical in size; segment VII semicircular; segment VIII narrow; segment IX reduced. Abdominal segment VIII dorsally with rounded, prominent, sclerotised abdominal protuberance (a–pr). Urogomphi (ur) vestigial, in the form of very short sclerotised, sharp protuberances. (Fig. 5C).

***Chaetotaxy*.** Well developed, setae medium to elongated, transparent. Head with one very short *sos*, one medium *os*, and one medium-sized *pas*. Rostrum with two *rs* and one minute *es* (Fig. 5A). Pronotum with two *as*, one *ds*, two *ls*, and four *pls* almost equal in length; *ds* located in shallow pits. Dorsal parts of meso- and metathorax with three setae of various lengths, placed medially. Apex of femora with two long *fes* (Fig. 5A–C). Procoxae with a single seta (*cs*). Abdominal segments I–VII with six setae dorsally, variable in length: first minute, placed anteromedially; second and fourth minute; third and fifth medium, placed close to posterior margin of the segment; six, medium, placed below stigma (on segments VI and VII all setae medium). Abdominal segment VIII with four elongated setae dorsally. Each lateral part of abdominal segments I–VII with a single medium seta. Ventral parts of abdominal segments I–VIII with four setae, median pair longer than other ventral setae. Abdominal segment IX with four medium-sized setae ventrally (Fig. 5A–C).

#### Remarks and comparative notes.

This species is widely distributed in central and southern Europe, in the states of the Caucasus, and in western and central Turkey (Alonso-Zarazaga et al. 2023). Recently, it was reported as introduced into the USA (DiGirolomo et al. 2019). It is clearly related to *R.tetra*, from which the adult differs by the longer rostrum, especially in the female, the elongated shape of the elytra, and the male genitalia (Caldara 2014).

#### Biological notes.

Larval hosts of *R.asellus* are restricted to species of *Verbascum* (*V.nigrum* L., *V.phlomoides* L., *V.pulverulentum* Vill., *V.sinuatum* L., *V.thapsoides* Schw., *V.thapsus*, and *V.virgatum* Stokes; Caldara 2014). The biology of this species was carefully studied by Gumovsky (2007). Adults can be found feeding on the shoots and leaves of the host plant beginning in May. Oviposition sites are typically concentrated in the top portion of the host plant. The female drills a hole in the host-plant tissues with her very long rostrum, and then she lays eggs there. Eggs develop in ~ 7 days. After hatching, the larva bores into the stem and feeds on plant tissues, often leading to swelling of the stem. Mature larvae form a pupal cell just beneath the outer layer of plant tissue within the stem. Larval and pupal development take on average 20 and 10 days, respectively. Sometimes *R.asellus* lives on the same host plant as *R.tetra*, although they occupy different niches.

### ﻿2) Rhinusa tetra

Taxon classificationAnimaliaColeopteraCurculionidae

(Fabricius, 1792)

5746F492-64AC-52A8-B593-7DED13B7FEF1

#### Material examined.

21 mature larvae; 5 ♂ and 2 ♀ pupae. Italy, Lombardia, Linarolo (Pavia), ex *Verbascumthapsus*, 25.08.2015; 19 mature larvae; 7 premature; 3 ♂ and 4 ♀ pupae, Italy, Alto Adige, Castelrotto (Bolzano), ex *Verbascumblattaria* L., 10.08. 2017, all leg., det. R. Caldara.

#### Description of mature larva

**Figure 6.**
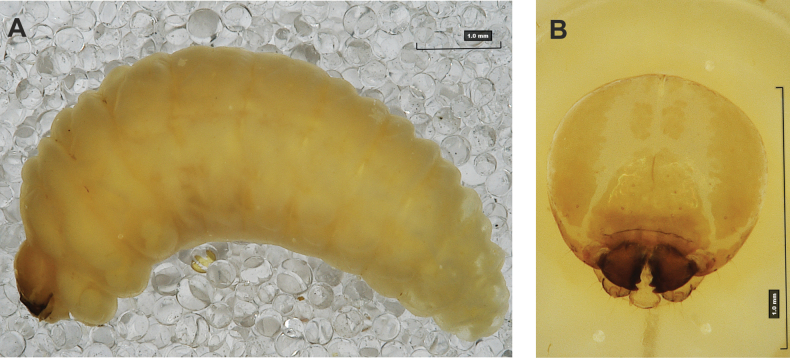
*Rhinusatetra* (Fabricius, 1792) mature larva **A** habitus **B** head, frontal view.

**Figure 7.**
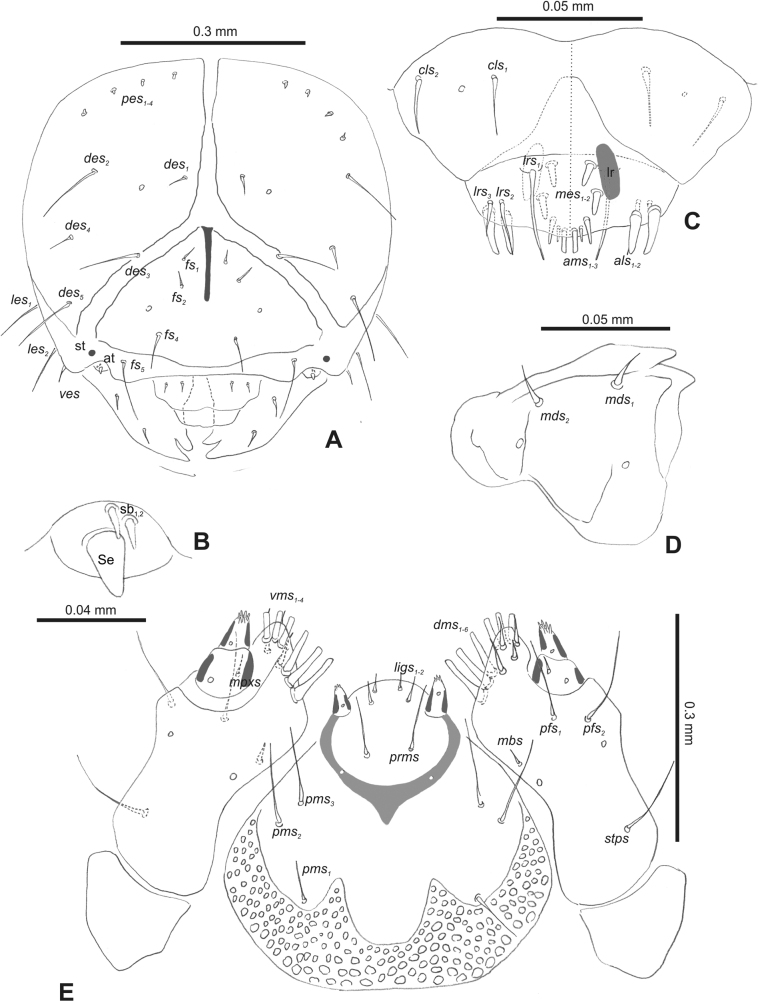
*Rhinusatetra* (Fabricius, 1792) mature larva, head and mouth parts **A** head **B** antenna **C** clypeus and labrum (left side), epipharynx (right side) **D** left mandible **E** maxillolabial complex (schemes). Abbreviations: at–antenna, lr–labral rods, sb–sensillum basiconicum, Se–sensorium, st–stemmata, setae: *als*–anterolateral, *ams*–anteromedial, *cls*–clypeal, *des*–dorsal epicranial, *dms*–dorsal malar, *fs*–frontal epicranial, *les*–lateral epicranial, *ligs*–ligular, *lrs*–labral, *mbs*–malar basiventral, *mds*–mandibular dorsal, *mes*–medial, *mpxs*–maxillary palp, *pes*–postepicranial, *pfs*–palpiferal, *pms*–postmental, *prms*–premental, *stps*–stipital, *ves*–ventral, *vms*–ventral malar.

**Figure 8.**
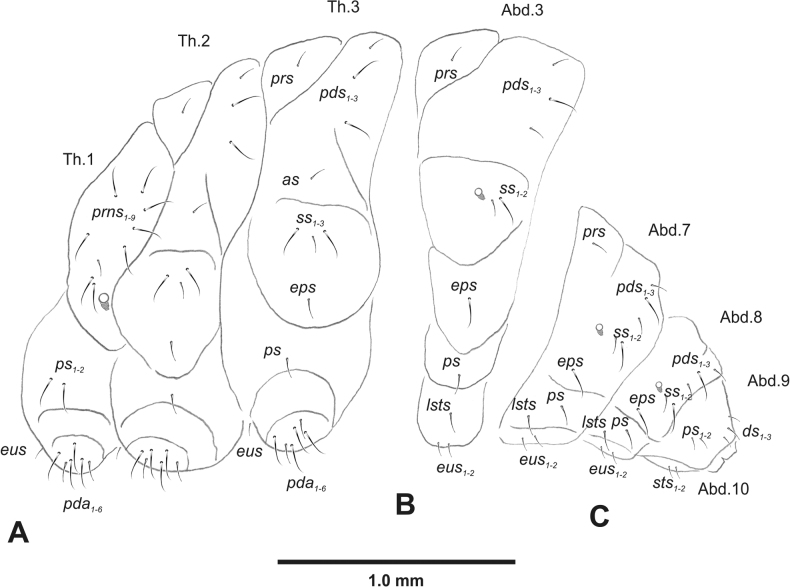
*Rhinusatetra* (Fabricius, 1792) mature larva, habitus **A** lateral view of thoracic segments **B** lateral view of abdominal segment I **C** lateral view of abdominal segments VII–X (schemes). Abbreviations: Th. 1–3–number of thoracic segments, Abd. 1–10–number of abdominal seg, setae: *as*–alar, *ds*–dorsal, *eps*–epipleural, *eus*–eusternal, *lsts*–laterosternal, *pda*–pedal, *pds*–postdorsal, *prns*–pronotal, *prs*–prodorsal, *ss*–spiracular, *ps*–pleural, *sts*–sternal.

**(Figs 6A, B, 7A–E, 8A–C). *Measurements*** (in mm). Body length: 3.25–6.50 (avg. 5.90). The widest place in the body (meso- and metathorax) measures up to 2.25. Head width: 0.85–0.90 (avg. 0.87).

***General*.** Body elongate, slender, curved, rounded in cross section (Fig. 6A). Prothorax smaller than mesothorax. Metathorax as wide as mesothorax; each divided dorsally into two folds (prodorsal fold much smaller than postdorsal fold). Pedal folds of thoracic segments prominent, conical, well isolated. Abdominal segments I–V of similar size, next segments tapering towards posterior body end. Abdominal segments I–VI each divided dorsally into two folds almost identical in size. Segments VII–IX dorsally undivided. Epipleural folds of segments I–VIII conical, well developed. Laterosternal and eusternal folds of segments I–VIII conical, well isolated. Abdominal segment X divided into four folds of equal size. Anus situated ventrally, almost completely hidden in segment IX.

Thoracic and abdominal spiracles unicameral; thoracic spiracles (Fig. 6A) placed laterally close to mesothorax; abdominal spiracles (Fig. 6A) placed medially on segments I–VIII.

***Colouration*.** Light yellow to brownish head (Fig. 6B). All thoracic and abdominal segments whitish (Fig. 6A). Cuticle densely covered with fine asperities.

***Vestiture*.** Setae on body thin, hair-like, different in length (minute to medium).

***Head capsule*** (Figs 6B, 7A). Head wide, endocarinal line present, reaching to 1/2 of the length of frons. Frontal sutures on head wide, indistinct. A single pair of stemmata in the form of small black spots (st) placed laterally, close to the end of the frontal suture. *Des_1_* very short, located in middle part of epicranium; very long *des_2_* located anteriorly; very long *des_3_* placed almost on the border of the frontal suture; very short *des_4_*, located laterally; and long *des_5_* placed anterolaterally above stemma (Fig. 7A). *Fs_1_* and *fs_2_* minute, located posterolaterally; *fs_3_* absent; *fs_4_* medium, located anteriorly; and long *fs_5_* located anterolaterally, close to antenna (Fig. 7A). *Les_1_* and *les_2_* medium; single short *ves*. Epicranial area with four *pes*.

***Antennae*** placed distally of the frontal suture, on the inside; membranous and distinctly convex basal article bearing one conical, moderately elongate sensorium, plus two sensilla basiconica (Fig. 7B).

***Clypeus*** (Fig. 7C) trapezoidal, ~ 2.5 × as wide as long with two relatively long *cls*, localised posterolaterally, with one sensillum between them; anterior border almost straight.

***Mouth parts*.** Labrum (Fig. 7C) ~ 2.2 × as wide as long, with three piliform *lrs*, various long; *lrs_1_* elongated, located posteromedially, on small protuberance, *lrs_2_* medium, located lateromedially, and *lrs_3_* short, located laterally; anterior border almost straight. Epipharynx (Fig. 7C) with two elongated finger-like *als* identical in length, three piliform *ams* variable in length, and two short, finger-like *mes*; labral rods (lr) distinct, kidney-shaped. Mandibles (Fig. 7D) bifid, cutting edge straight; two medium piliform and short *mds*, both located close to lateral border. Maxillolabial complex: maxilla brownish sclerotised (Fig. 7E) stipes with one *stps*, two *pfs* and one short *mbs*, *stps* and both *pfs_1–2_* elongated; mala with six finger-like *dms* variable in length; four medium piliform *vms*. Maxillary palpi two-segmented; basal palpomere distinctly wider than distal one; both palpomeres equal in length; basal palpomere with short *mpxs* and two sensilla, distal palpomere with a group of six apical sensilla in terminal receptive area. Prementum (Fig. 7E) close to oval-shaped, with a single elongated *prms*; ligula with rounded margin and two, short *ligs*; premental sclerite broad, sclerotised, cup-shaped, posterior extension short with thick apex. Labial palpi one-segmented; palpi with a single pore, and four apical sensilla in terminal receptive area; surface of labium smooth. Postmentum (Fig. 7E) with three *pms*, medium *pms_1_* located posteromedially, long *pms_2_* located mediolaterally, and elongated *pms_3_* located anterolaterally; membranous area partially covered with knobby asperities.

***Thorax*.** Prothorax (Fig. 8A) with eight elongated and single medium *prns*; two elongated *ps*; and single short *eus*. Mesothorax (Fig. 8A) with a single short *prs*; two medium and one short *pds* (ordered: medium, medium, short); one short *as*; two medium and one short *ss*; one medium *eps*; one medium *ps*; and single minute *eus*. Chaetotaxy of metathorax (Fig. 8A) almost identical to that of mesothorax. Each pedal area of thoracic segments with five elongated and one short *pda*.

***Abdomen*.** Segments I–VIII (Fig. 8B, C) with one very short *prs* (segment VIII without *prs*), three *pds* of various length; one medium and one long *ss*; single elongated *eps*; one medium *ps*; one short *lsts*; and two minute *eus*. Abdominal segment IX (Fig. 8C) with one short and two minute *ds*; two minute *ps*; and two minute *sts*.

#### Description of pupa

**Figure 9.**
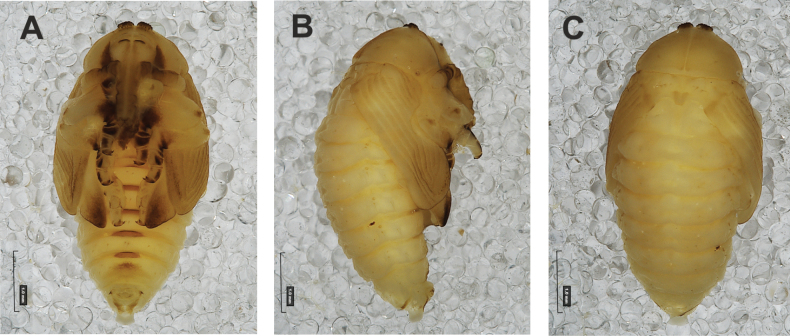
*Rhinusatetra* (Fabricius, 1792) pupa habitus **A** ventral view **B** lateral view **C** dorsal view.

**Figure 10.**
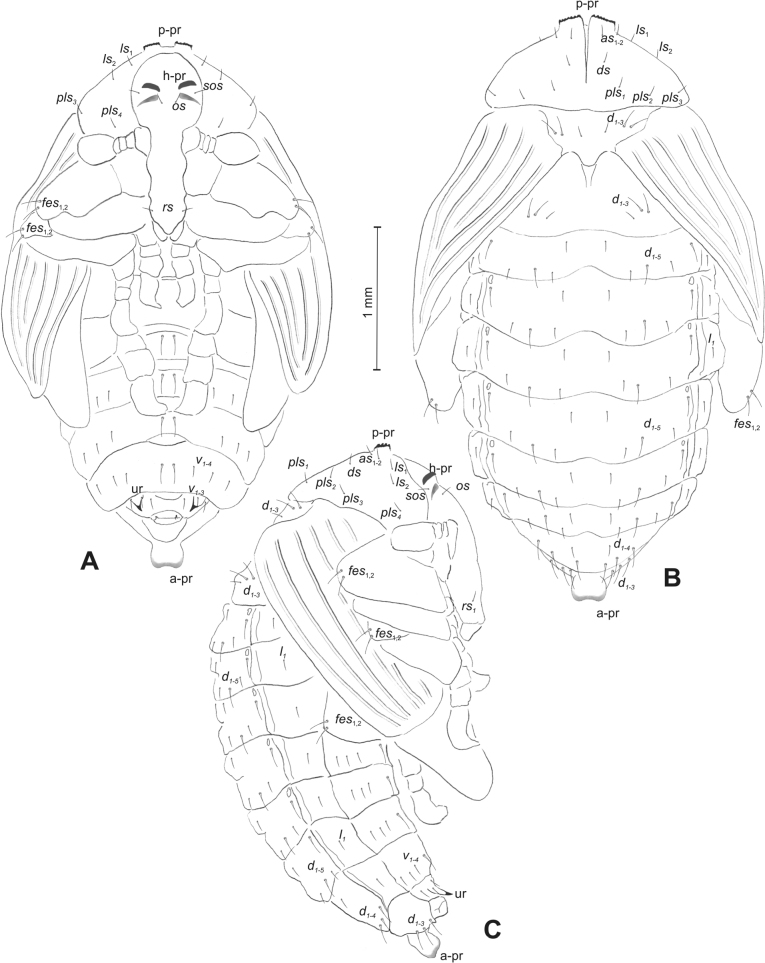
*Rhinusatetra* (Fabricius, 1792) pupa habitus **A** ventral view **B** dorsal view **C** lateral view (schemes). Abbreviations: a–pr–abdominal protuberances, h–pr–head protuberances, p–pr–pronotal protuberances, ur–urogomphi, setae: *as*–apical, *d*–dorsal, *ds*–discal, *fes*–femoral, *l*, *ls*–lateral, *os*–orbital, *pls*–posterolateral, *sos*–supraorbital, *rs*–rostral, *v*–ventral.

**(Figs 9A–C, 10A–C). *Measurements*** (in mm). Body length: 3.75–5.25; body width: 2.40–2.75; thorax width: 1.05–1.75; rostrum length: up to 0.70 ♂ and ♀.

***Body*.** Integument white, with some parts dark sclerotised; moderately elongated. Elongated head protuberances (h–pr) present on head above eyes. Rostrum moderately elongated, on both sexes almost 2.8 × as long as wide and protruding mesocoxae. Pronotum trapezoidal 2 × as wide as long. Pronotal protuberances (p–pr) flattened, sclerotised, separated at bases. Mesonotum slightly narrower than metanotum. Abdominal segments I–VI almost identical in size, VII semicircular, segment VIII narrow, segment IX reduced. Abdominal segment VIII dorsally with rounded, well developed abdominal protuberance (a–pr). Urogomphi (ur) very short, ending with sclerotised, sharp apexes (Fig. 9A–C).

***Chaetotaxy*.** Well developed, setae medium to short. Head with one medium *sos* and one medium *os*. Rostrum with a single *rs* (Fig. 10A). Pronotum with two *as*, two *ls*, single *ds*, and four *pls*, all equal in length. Dorsal parts of meso- and metathorax with three setae of various length, placed medially. Apex of femora with two long *fes* (Fig. 10A–C). Abdominal segments I–VII dorsally with five setae dorsally, variable in length: first, second and fourth minute, third and fifth medium; first seta placed posteromedially, second to fourth placed close to posterior margin of the segment, fifth placed below stigma. Segment VII with four elongated setae dorsally, segment VIII with three elongated setae dorsally. Each lateral part of abdominal segments I–VIII with a single short seta. Ventral parts of abdominal segments I–VIII with four setae (first distinctly longer than other setae). Abdominal segment IX with three short setae ventrally (Fig. 10A–C).

#### Remarks and comparative notes.

This species is one of the most common and widespread of the genus *Rhinusa*. It has been reported in all of Europe, Siberia, North Africa, the Middle East, central Asia, and northern India. It was accidentally introduced in North America, where it is currently distributed in several states of the USA and Canada (O’Brien and Wibmer 1982; DiGirolomo et al. 2019). Although it was proposed as a potential candidate for the biological control of invasive common mullein, *Verbascumthapsus*, it was not used in North America in this regard. In the southern part of its area of distribution, it can be confused with other species of the group, such as *R.verbasci* (Rosenschoeld, 1838), *R.moroderi* (Reitter, 1906), and *R.weilli* Caldara, 2014, from which it can be separated only by the shape of the female rostrum, most easily if observed in lateral view (Caldara 2014). It is well known that adults of *R.tetra* are highly variable in size (2.0–4.5 mm), even among specimens collected from the same plant. Additionally, the rostrum length of the female distinctly varies among populations living on different plants, probably related to oviposition inside seed capsules of different sizes and thicknesses, as suggested to occur in *R.dieckmanni* (Behne, 1988) (Toševski et al. 2023).

#### Biological notes.

This species was reported to feed on several species of *Verbascum*. Caldara et al. (2012) verified the following plant associations: *Verbascumblattaria*, *V.boerhavii* L., *V.creticum* (L.) Cav., *V.lychnitis* L., *V.nigrum*, *V.phlomoides*, *V.phoeniceum* L., *V.pulverulentum*, *V.speciosum* Schrader, *V.thapsiforme* Schrader, and *V.thapsus*. Adults were also collected on *Scrophulariaauriculata* L., *S.canina* L., and *S.laevigata* Vahl.

Marquess (2000) provides very detailed information on the biology of this species. Adults feed on the dorsal surfaces of leaves and on the seed capsules of the host plant. Once a plant flowers, mating occurs on the entire inflorescence. The female usually oviposits 1–3 eggs per seed capsule. Egg hatching occurs 7–11 days after deposition. Larvae feed on seeds within capsules and consume the majority of them. Pupation occurs within the seed capsule, and the emergence of the adults occurs ~ 25 days later. Adults exit the seed capsule by chewing through the hardened pericarp.

##### ﻿*Rhinusaantirrhini* group

**Adult diagnosis.** Rostrum in dorsal view from base to antennal insertion with a trapezoidal outline, with dorsal part narrower than ventral part, in male with distinctly visible scrobe; rostrum in lateral view with dorsal margin abruptly narrowed in apical part; first elytral interstria apically covered with dense vestiture.

### ﻿3) Rhinusa antirrhini

Taxon classificationAnimaliaColeopteraCurculionidae

(Paykull, 1800)

6415E23D-EC1F-570B-88EE-4064A7781411

#### Material examined.

29 mature larvae; 28 ♂ and 26 ♀ pupae. Serbia, Zemun, ex *Linariavulgaris* Mill., 15.08.2017, leg., det. I. Toševski.

#### Description of mature larva

**Figure 11.**
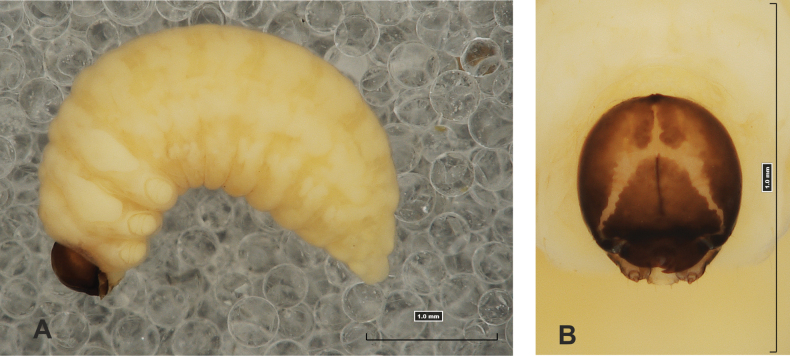
*Rhinusaantirrhini* (Paykull, 1800) mature larva **A** habitus **B** head, frontal view.

**Figure 12.**
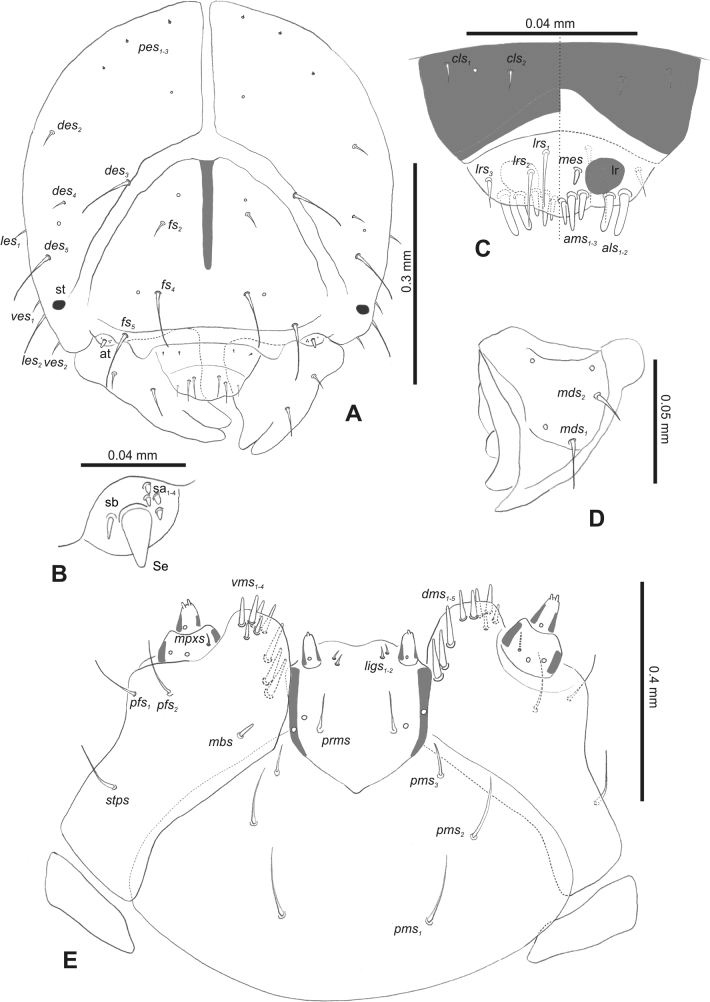
*Rhinusaantirrhini* (Paykull, 1800) mature larva, head and mouth parts **A** head **B** antenna **C** clypeus and labrum (left side), epipharynx (right side) **D** left mandible **E** maxillolabial complex (schemes). Abbreviations: at–antenna, lr–labral rods, sa–sensillum ampullaceum, sb–sensillum basiconicum, Se–sensorium, st–stemmata, setae: *als*–anterolateral, *ams*–anteromedial, *cls*–clypeal, *des*–dorsal epicranial, *dms*–dorsal malar, *fs*–frontal epicranial, *les*–lateral epicranial, *ligs*–ligular, *lrs*–labral, *mbs*–malar basiventral, *mds*–mandibular dorsal, *mes*–medial, *mpxs*–maxillary palp, *pes*–postepicranial, *pfs*–palpiferal, *pms*–postmental, *prms*–premental, *stps*–stipital, *ves*–ventral, *vms*–ventral malar.

**Figure 13.**
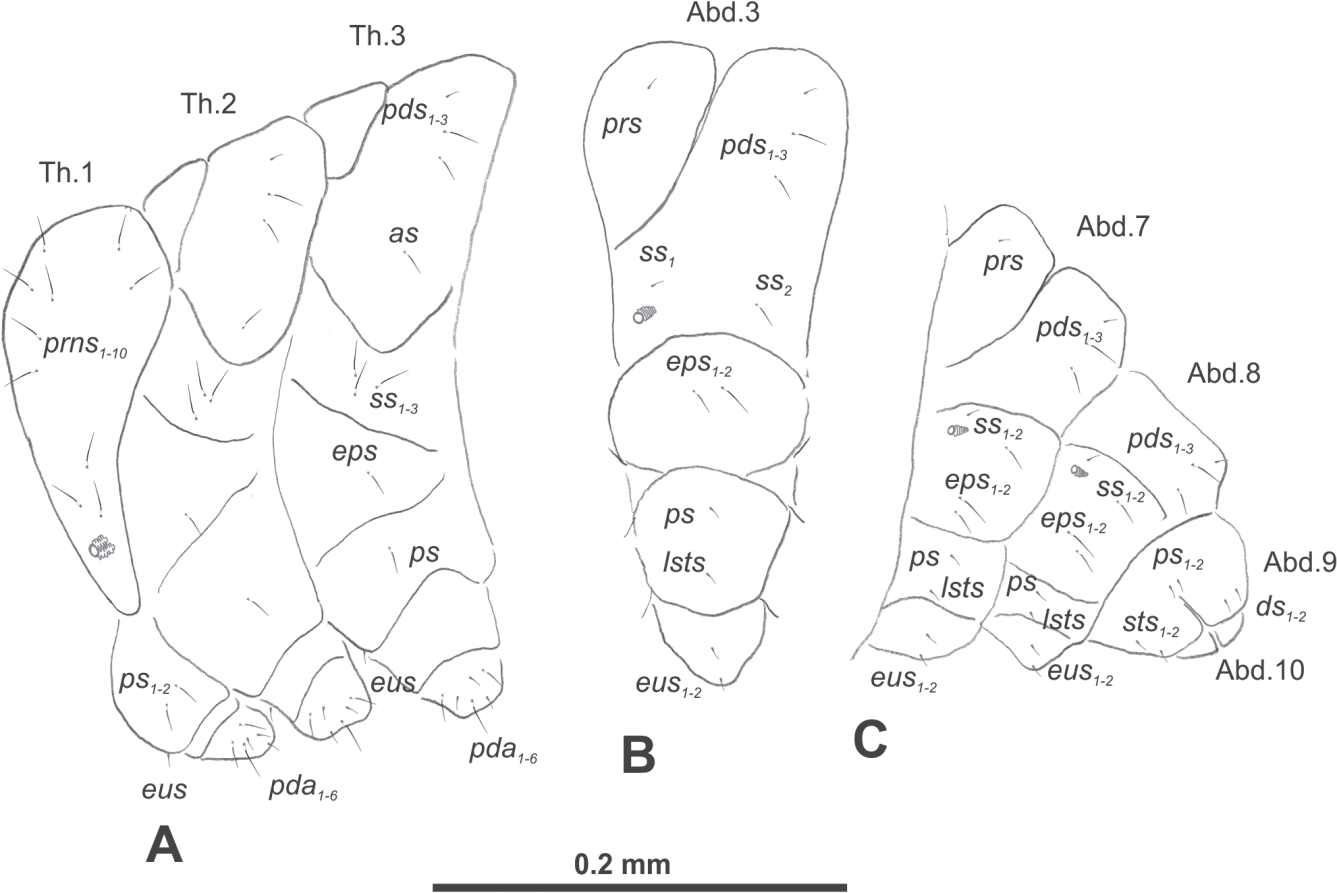
*Rhinusaantirrhini* (Paykull, 1800) mature larva, habitus **A** lateral view of thoracic segments **B** lateral view of abdominal segment I **C** lateral view of abdominal segments VII–X (schemes). Abbreviations: Th. 1–3–number of thoracic segments, Abd. 1–10–number of abdominal seg, setae: *as*–alar, *ds*–dorsal, *eps*–epipleural, *eus*–eusternal, *lsts*–laterosternal, *pda*–pedal, *pds*–postdorsal, *prns*–pronotal, *prs*–prodorsal, *ss*–spiracular, *ps*–pleural, *sts*–sternal.

**(Figs 11A, B, 12A–E, 13A–C). *Measurements*** (in mm). Body length: 3.00–4.25 (avg. 3.60). The widest place in the body (meso- and metathorax) measures up to 1.25. Head width: 0.55–0.60 (avg. 0.56).

***General*.** Body elongate, slender, distinctly curved, rounded in cross section (Fig. 11A). All thoracic segments almost equal in size. Meso- and metathorax each divided dorsally into two folds (prodorsal fold distinctly smaller than postdorsal fold). Pedal folds of thoracic segments isolated, conical, prominent. Abdominal segments I–VI of similar size, next segments tapering towards posterior body end. Abdominal segments I–VII each divided dorsally into two folds: prodorsal fold slightly smaller than postdorsal; both folds equally in high. Segments VIII and IX dorsally undivided. Epipleural folds of segments I–VIII conical. Laterosternal and eusternal folds of segments I–VIII conical, weakly isolated. Abdominal segment X (almost completely hidden in previous segment) divided into four folds of equal size. Anus situated ventrally.

Thoracic spiracles bicameral, abdominal unicameral; thoracic spiracles (Fig. 11A) placed laterally close to mesothorax; abdominal spiracles (Fig. 11A) placed antero-laterally on segments I–VIII.

***Colouration*.** All thoracic and abdominal segments whitish (Fig. 11A). Cuticle densely covered with fine asperities. Dark yellow to dark brown head, medial parts of epicranium less sclerotised (Fig. 11B).

***Vestiture*.** Setae on body thin, yellowish, different in length (very short or medium).

***Head capsule*** (Figs 11B, 12A). Head slightly narrowed bilaterally, endocarinal line present, reaching to the 2/3 of the length of frons. Frontal sutures on head very wide, indistinct. Single pair of stemmata in the form of small black spots (st) close to the end of the frontal suture. *Des_1_* absent; *des_2_* short, located in lateral part of epicranium; long *des_3_* located anteriorly on epicranium on border of the frontal suture; *des_4_* minute; and *des_5_* long, located anterolaterally above stemma (Fig. 12A). *Fs_1_* absent; *fs_2_* short, located medially; *fs_3_* absent; *fs_4_* long, located anteriorly; and long *fs_5_* located anterolaterally, close to antenna (Fig. 12A). *Les_1_* short and medium *les_2_*; two short *ves*. Epicranial area with three *pes*.

***Antennae*** placed distally of the frontal suture, on the inside; membranous and distinctly convex basal article bearing one conical sensorium, plus four sensilla ampullacea and single sensillum basiconicum (Fig. 12B).

***Clypeus*** (Fig. 12C) trapezoidal, ~ 3.2 × as wide as long with two short *cls*, localised posterolaterally, with one sensillum between them; except the posterior part, whole clypeus darkly sclerotised; anterior border slightly curved towards the inside.

***Mouth parts*.** Labrum (Fig. 12C) ~ 2.2 × as wide as long, with three piliform *lrs*, various long; *lrs_1_* and *lrs_2_* elongated, located medially, and *lrs_3_* short, located laterally; anterior border bi-sinuate. Epipharynx (Fig. 12C) with two relatively elongated finger-like *als*, almost identical in length; three *ams* various in size; and single short finger-like *mes*; labral rods (lr) distinct, rounded. Mandibles (Fig. 12D) bifid, cutting edge with additional protuberance; two medium piliform *mds*, both located close to lateral border. Maxillolabial complex: maxilla more sclerotised than labium (Fig. 12E) stipes with one *stps*, two *pfs* and one short *mbs*, *stps*, and both *pfs_1–2_* medium; mala with five finger-like *dms* variable in length; four piliform *vms*, medium to short in length. Maxillary palpi two-segmented; basal palpomere distinctly wider than distal one; length ratio of basal and distal palpomeres almost 1:1; basal palpomere with short *mpxs* and two sensilla, distal palpomere with a group of two or three apical sensilla in terminal receptive area. Prementum (Fig. 12E) close to oval-shaped, with one medium *prms*; ligula with round margin and two short *ligs*; premental sclerite vestigial, only lateral parts dark sclerotised. Labial palpi one-segmented; each palp with a single pore, and a group of one or two apical sensilla (ampullacea) on terminal receptive area; surface of labium smooth. Postmentum (Fig. 12E) with three *pms*, elongated *pms_1_* located medially, long *pms_2_* located laterally, and medium *pms_3_* located antero-laterally; membranous area smooth.

***Thorax*.** Prothorax (Fig. 13A) with ten relatively long *prns*; two medium *ps*; and single short *eus*. Mesothorax (Fig. 13A) without *prs*; one minute and two medium *pds*; one medium *as*; three medium *ss*; one medium *eps*; one medium *ps*; and single minute *eus*. Chaetotaxy of metathorax (Fig. 13A) almost identical to that of mesothorax. Each pedal area of thoracic segments with three medium and three minute *pda*.

***Abdomen*.** Segments I–VIII (Fig. 13B, C) with one minute *prs* (segment VIII without); one minute and two medium *pds*; one minute and one medium *ss*; two medium *eps*; one minute *ps*; one minute *lsts*; and two minute *eus*. Abdominal segment IX (Fig. 13C) with two minute *ds*; two minute *ps*; and two minute *sts*.

#### Description of pupa

**Figure 14.**
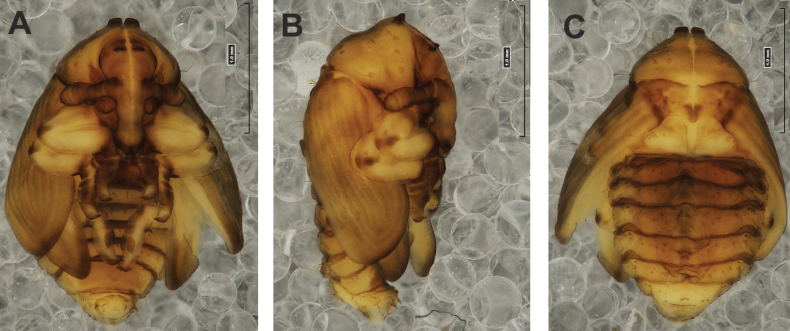
*Rhinusaantirrhini* (Paykull, 1800) pupa habitus **A** ventral view **B** lateral view **C** dorsal view.

**Figure 15.**
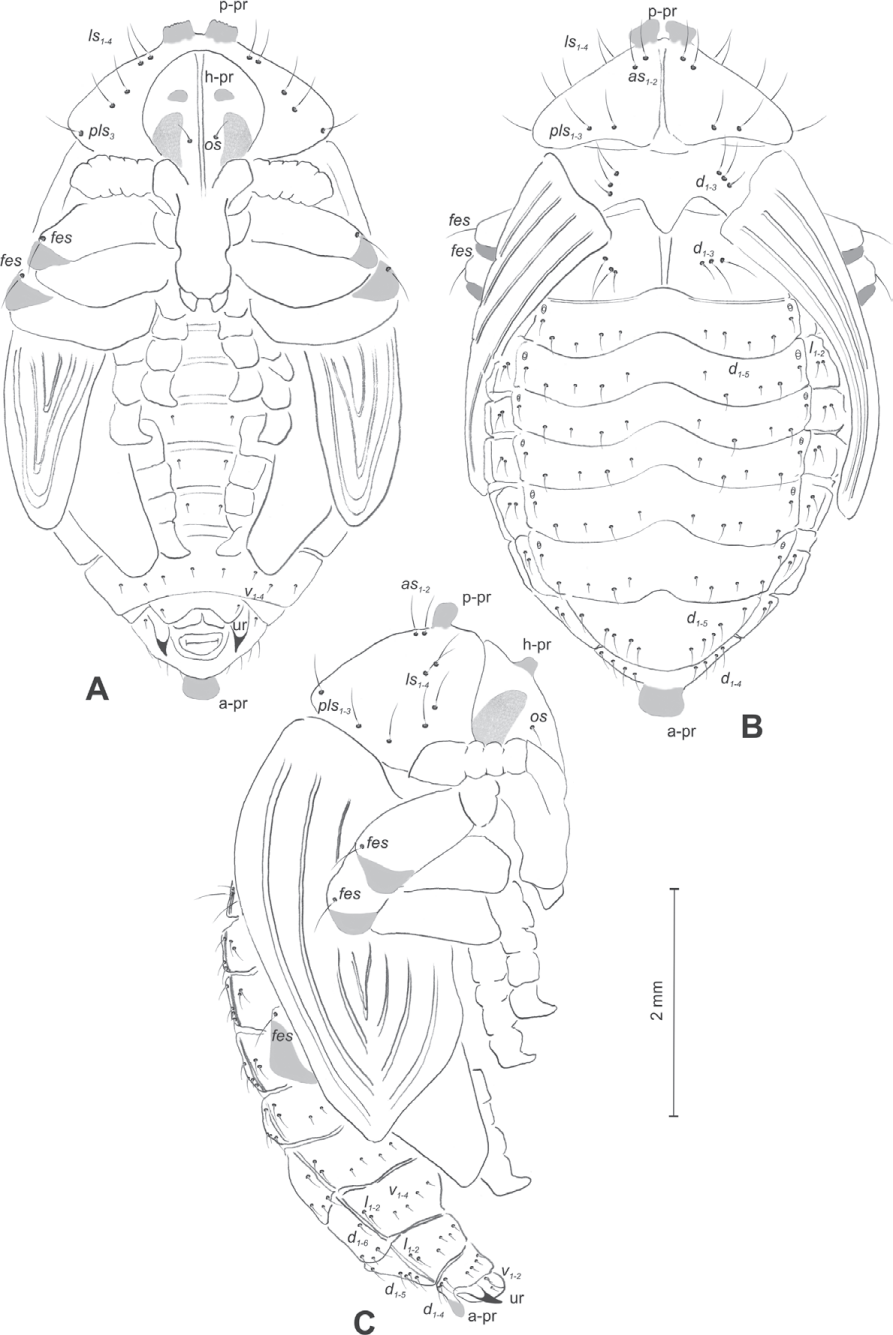
*Rhinusaantirrhini* (Paykull, 1800) pupa habitus **A** ventral view **B** dorsal view **C** lateral view (schemes). Abbreviations: a–pr–abdominal protuberances, h–pr–head protuberances, p–pr–pronotal protuberances, ur–urogomphi, setae: *as*–apical, *d*–dorsal, *fes*–femoral, *l*, *ls*–lateral, *os*–orbital, *pls*–posterolateral, *v*–ventral.

**(Figs 14A–C, 15A–C). *Measurements*** (in mm). Body length: 2.35–3.30 (avg. 2.75); body width: 1.45–1.85 (avg. 1.60); thorax width: 0.90–1.20 (avg. 1.05); rostrum length: up to 0.60 ♂ and 0.75 ♀.

***Body*.** Integument white, with some parts dark sclerotised; moderately stout, curved (Fig. 14A–C). All pronotal setae placed on pigmented spots. Head with a pair of distinct head protuberances (h–pr) above eyes. Rostrum rather short, in male usually only slightly shorter than in female: almost 3 × as long as wide, reaching mesocoxae. Pronotum trapezoidal 2 × as wide as long. Pronotal protuberances (p–pr) conical, flattened, sclerotised, separated at bases. Mesonotum and metanotum similar in size. Abdominal segments I–VI almost identical in size; segment VII semicircular; segment VIII narrow; segment IX reduced. Abdominal segment VIII dorsally with rounded, prominent, sclerotised abdominal protuberance (a–pr). Urogomphi (ur) short, ending with sclerotised, sharp apexes (Fig. 15A–C).

***Chaetotaxy*.** Well developed, setae short to elongated, transparent. Head with one short *os* (Fig. 15A). Pronotum with two *as*, four *ls*, and three *pls* almost equally in length. Dorsal parts of meso- and metathorax with three setae of various length, placed medially. Apex of femora with a single long *fes* (Fig. 15A–C). Abdominal segments I–VII with five setae dorsally, variable in length: first and third minute, second, fourth and fifth medium; first to fourth placed close to posterior margin of the segment, fifth placed below stigma (on segments VI and VII all setae medium). Abdominal segment VIII with four elongated setae dorsally. Each lateral part of abdominal segments I–VII with two medium setae. Ventral parts of abdominal segments I–VIII with four medium-sized setae. Abdominal segment IX with two medium setae ventrally (Fig. 15A–C).

#### Remarks and comparative notes.

This species is reported from all of Europe, although it is probable that, especially in the Balkans, it is confused with several cryptic species still to be described on the basis of molecular studies (Hernández-Vera et al. 2010; IT, pers. obs.). *Rhinusaantirrhini* lives on *Linaria* spp. other than *L.vulgaris*. This weevil was accidentally imported into North America at the beginning of the twentieth century, where it became important in applied entomology when proposed for the biological control of the invasive plant species *L.vulgaris* (see Hernández-Vera et al. 2010 and DiGirolomo et al. 2019 for references). The adult is sometimes confused with *R.dieckmanni*, as recently shown by Toševski et al. (2023).

#### Biological notes.

This univoltine weevil feeds on *L.vulgaris* and *L.angustissima* (Loisel.) Borbás, in the capsules of which larvae and pupae can be found. Adults emerge at the end of May, visiting flowers to start consuming pollen, and shortly after that, copulation begins. After mating, the females start laying eggs in well-developed floral ovaries, usually in their upper parts. When oviposition occurs, the outside of the seed capsule has a long, cone-shaped protrusion immediately above the oviposition site. This drives the formation of a semi-gall in that area. The early instar larvae feed primarily on hypertrophied seeds, while older and later instars consume abortive seeds. Most often, a single seed capsule contains one *R.antirrhini* larva, while at high densities, two larvae per capsule are commonly found. Development to adult takes ~ 30–50 days, depending on the environmental temperature. Newly emerged adults feed on young shoots of their host plant, expressing irregular aestivation periods until mid-autumn, when adults intensify feeding just before entering hibernation. Adults overwinter at the soil surface, sheltered below or between dry plant remains (IT, pers. obs.).

### ﻿4) Rhinusa florum

Taxon classificationAnimaliaColeopteraCurculionidae

(Rubsaamen, 1895)

E3DC485C-F46D-5CE5-B041-3F7152892BD1

#### Material examined.

19 mature larvae; 20 ♂ and 6 ♀ pupae. Serbia, Vinci, ex *Linariagenistifolia* (L.) Miller, 05.06.2017, leg., det. I. Toševski.

#### Description of mature larva

**Figure 16.**
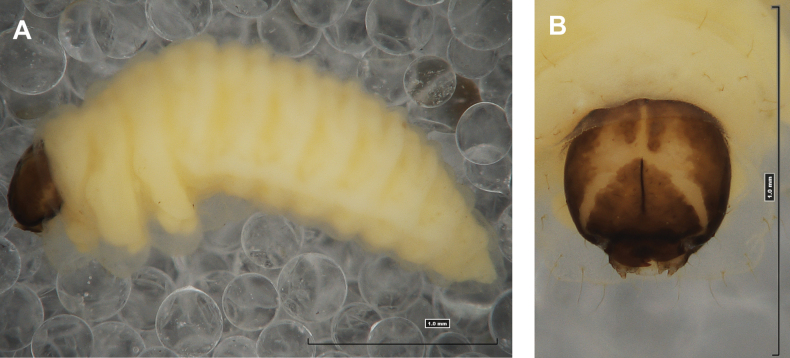
*Rhinusaflorum* (Rubsaamen, 1895) mature larva **A** habitus **B** head, frontal view.

**Figure 17.**
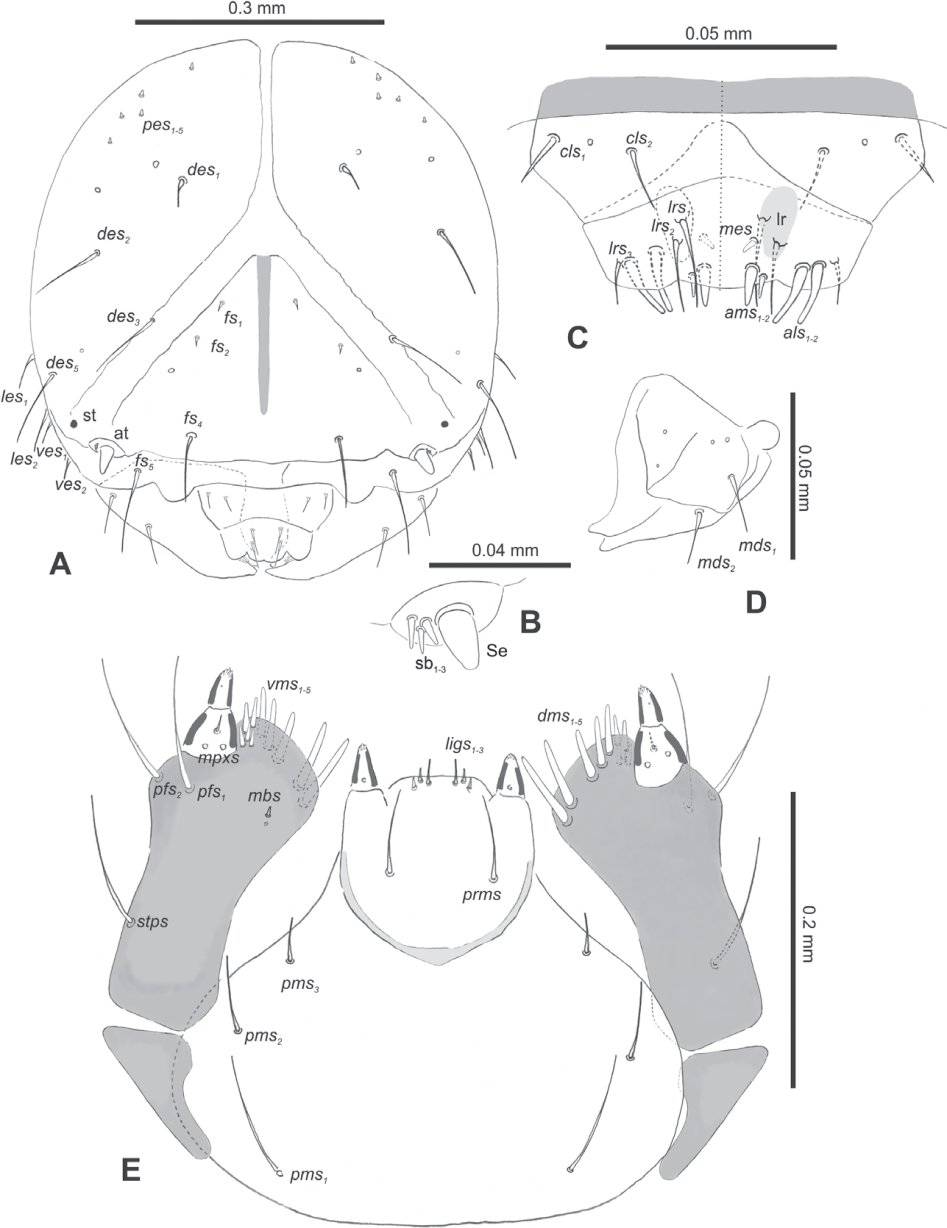
*Rhinusaflorum* (Rubsaamen, 1895) mature larva, head and mouth parts **A** head **B** antenna **C** clypeus and labrum (left side), epipharynx (right side) **D** left mandible **E** maxillolabial complex (schemes). Abbreviations: at–antenna, lr–labral rods, sb–sensillum basiconicum, Se–sensorium, st–stemmata, setae: *als*–anterolateral, *ams*–anteromedial, *cls*–clypeal, *des*–dorsal epicranial, *dms*–dorsal malar, *fs*–frontal epicranial, *les*–lateral epicranial, *ligs*–ligular, *lrs*–labral, *mbs*–malar basiventral, *mds*–mandibular dorsal, *mes*–medial, *mpxs*–maxillary palp, *pes*–postepicranial, *pfs*–palpiferal, *pms*–postmental, *prms*–premental, *stps*–stipital, *ves*–ventral, *vms*–ventral malar.

**Figure 18.**
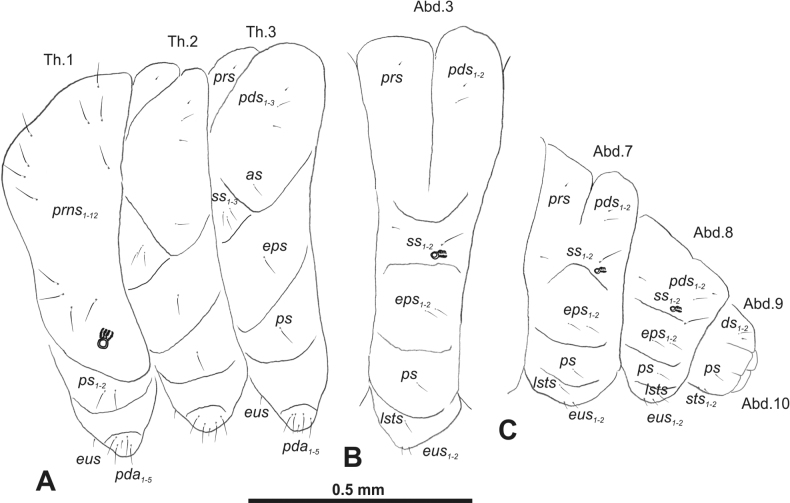
*Rhinusaflorum* (Rubsaamen, 1895) mature larva, habitus **A** lateral view of thoracic segments **B** lateral view of abdominal segment I **C** lateral view of abdominal segments VII–X (schemes). Abbreviations: Th. 1–3–number of thoracic segments, Abd. 1–10–number of abdominal seg, setae: *as*–alar, *ds*–dorsal, *eps*–epipleural, *eus*–eusternal, *lsts*–laterosternal, *pda*–pedal, *pds*–postdorsal, *prns*–pronotal, *prs*–prodorsal, *ss*–spiracular, *ps*–pleural, *sts*–sternal.

**(Figs 16A, B, 17A–E, 18A–C). *Measurements*** (in mm). Body length: 2.00–3.50 (avg. 3.00). The widest place in the body (meso- and metathorax) measures up to 1.20. Head width: 0.46–0.54 (avg. 0.52).

***General*.** Body elongate, slightly curved, oblate dorsoventrally in cross section (Fig. 16A). Prothorax prominent, pronotal shield not pigmented; meso-­ and metathorax equal in size, smaller than prothorax. Meso- and metathorax each divided dorsally into two folds (prodorsal fold distinctly smaller than postdorsal fold). Pedal folds of thoracic segments isolated, conical, prominent. Abdominal segments I–VI of similar size, next segments tapering towards posterior body end. Abdominal segments I–VII each divided dorsally into two almost equal in size folds; postdorsal folds only slightly higher than prodorsal folds. Segments VIII and IX dorsally undivided. Epipleural folds of segments I–VIII conical. Laterosternal and eusternal folds of segments I–VIII conical, weakly isolated. Abdominal segment X divided into four folds of equal size. Anus situated ventrally, almost completely covered with the ninth abdominal segment.

Thoracic spiracles bicameral, abdominal unicameral; thoracic spiracles (Fig. 16A) placed laterally close to mesothorax; abdominal spiracles (Fig. 16A) placed mediolaterally on segments I–VIII.

***Colouration*.** Cuticle covered with fine asperities. Brown head, medial parts of epicranium less sclerotised (Fig. 16B). All thoracic and abdominal segments whitish (Fig. 16A).

***Vestiture*.** Setae on body thin, yellowish, different in length (very short or medium).

***Head capsule*** (Figs 16B, 17A). Head suboval, endocarinal line present, reaching to the 3/4 of the length of frons. Frontal sutures on head distinct, very wide. Single pair of stemmata in the form of small black spots (st) close to the end of the frontal suture. *Des_1_* short, located in middle part of epicranium; long *des_2_*; long *des_3_* located anteriorly on epicranium close to the border with the frontal suture; *des_4_* absent; and *des_5_* long, located anterolaterally above stemma (Fig. 17A). *Fs_1_* and *fs_2_* short, located medially; *fs_3_* absent; long *fs_4_* located anteriorly; and long *fs_5_* located anterolaterally, close to antenna (Fig. 17A). *Les_1_* and *les_2_* medium; single medium *ves*. Epicranial area with five *pes*.

***Antennae*** placed distally of the frontal suture, on the inside; membranous and distinctly convex basal article bearing one conical, slightly elongate sensorium, plus three sensilla basiconica (Fig. 17B).

***Clypeus*** (Fig. 17C) trapezoidal, ~ 3.6 × as wide as long with two relatively long *cls*, localised posterolaterally, with one sensillum between them; basal part distinctly sclerotised; anterior border curved towards the inside.

***Mouth parts*.** Labrum (Fig. 17C) ~ 3 × as wide as long, with three piliform *lrs*, variously long; *lrs_1_* and *lrs_2_* elongated, located medially, *lrs_3_* short, located anterolaterally; anterior border bi-sinuate. Epipharynx (Fig. 17C) with two relatively elongated finger-like *als*, almost identical in length; two piliform *ams* various in size; and single short finger-like *mes*; labral rods (lr) close to kidney-shaped. Mandibles (Fig. 17D) bifid, cutting edge straight; two medium piliform *mds*, both located in shallow pits, close to lateral border. Maxillolabial complex: maxilla dark sclerotised (Fig. 17E), stipes with one *stps*, two *pfs*, and one very short *mbs* and one sensillum, *stps* and both *pfs_1–2_* relatively long; mala with five finger-like *dms* variable in length (first and second much longer than others); five piliform *vms*, medium to short in length. Maxillary palpi two-segmented; basal palpomere distinctly wider than distal one; length ratio of basal and distal palpomeres almost 1:1; basal palpomere with short *mpxs* and two sensilla, distal palpomere with a group of six apical sensilla in terminal receptive area. Prementum (Fig. 17E) oval-shaped, with one long *prms*; ligula with round margin and three short *ligs*; premental sclerite vestigial, only basal part highly sclerotised. Labial palpi one-segmented; palpi with a single pore, and a group of five apical sensilla (ampullacea) on terminal receptive area; surface of labium smooth. Postmentum (Fig. 17E) with three *pms*, elongated *pms_1_* located medially, medium *pms_2_* located laterally, and relatively short *pms_3_* located antero-laterally; membranous area smooth.

***Thorax*.** Prothorax (Fig. 18A) with 12 medium *prns*, dorsal sclerite weakly visible; two medium *ps*; and single short *eus*. Mesothorax (Fig. 18A) with one minute *prs*, two minute and one medium *pds* (ordered: minute, medium, minute); one medium *as*; three medium *ss*; one medium *eps*; one medium *ps*; and single minute *eus*. Chaetotaxy of metathorax (Fig. 18A) almost identical to that of mesothorax. Each pedal area of thoracic segments with four medium and one minute *pda*.

***Abdomen*.** Segments I–VIII (Fig. 18B, C) with one minute *prs* (segment VIII without); one minute and one medium *pds*; one minute and one medium *ss*; two medium *eps*; one medium *ps*; one medium *lsts*; and two short *eus*. Abdominal segment IX (Fig. 18C) with one minute and medium *ds*; one medium *ps*; and two medium *sts*.

#### Description of pupa

**Figure 19.**
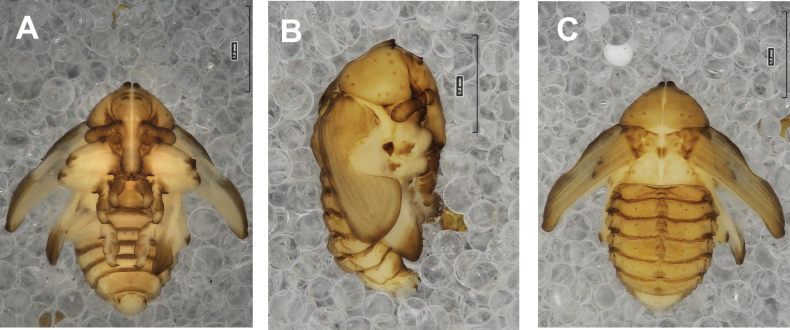
*Rhinusaflorum* (Rubsaamen, 1895) pupa habitus **A** ventral view **B** lateral view **C** dorsal view.

**Figure 20.**
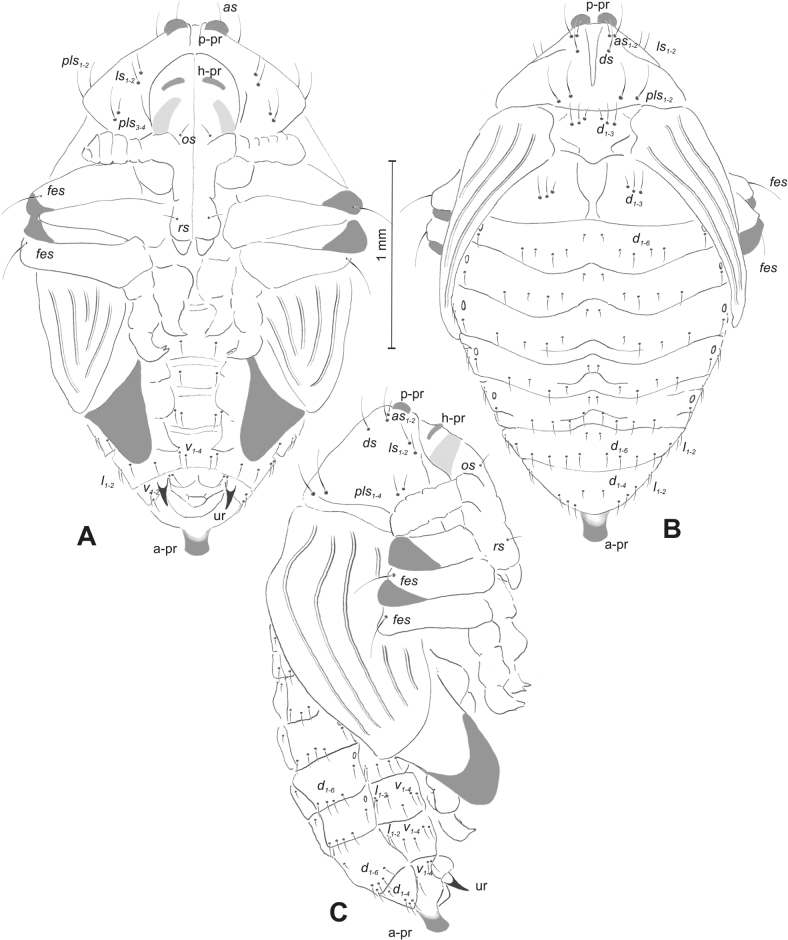
*Rhinusaflorum* (Rubsaamen, 1895) pupa habitus **A** ventral view **B** dorsal view **C** lateral view (schemes). Abbreviations: a–pr–abdominal protuberances, h–pr–head protuberances, p–pr–pronotal protuberances, ur–urogomphi, setae: *as*–apical, *d*–dorsal, *ds*–discal, *fes*–femoral, *l*, *ls*–lateral, *os*–orbital, *pls*–posterolateral, *rs*–rostral, *v*–ventral.

**(Figs 19A–C, 20A–C). *Measurements*** (in mm). Body length: 1.86–2.93 (avg. 2.60); body width: 1.66–1.86 (avg. 1.80); thorax width: 1.00–1.16 (avg. 1.06); rostrum length: up to 0.66 ♂ and 0.73 ♀.

***Body*.** Integument white, with some parts dark sclerotised; moderately stout, curved. All setae placed on dark brown spots. Head protuberances (h–pr) present on head above eyes. Rostrum rather short, in male usually only slightly shorter than in female almost 2.3 × as long as wide, reaching mesocoxae. Pronotum trapezoidal 2.4 × as wide as long. Pronotal protuberances (p–pr) conical, sclerotised, separated at bases. Meso- and metanotum similar in size. Abdominal segments I–VI almost identical in size; segment VII semicircular; segment VIII narrow; segment IX reduced. Abdominal segment VIII dorsally with rounded, prominent, sclerotised abdominal protuberance (a–pr). Urogomphi (ur) medium, ending with sclerotised, sharp apexes (Fig. 19A–C).

***Chaetotaxy*.** Well developed, setae medium to elongated, transparent. Head with one medium *os*. Rostrum with a single *rs* (Fig. 20A). Pronotum with two *as*, one *ds*, two *ls*, and four *pls* variable in length. Dorsal parts of meso- and metathorax with three setae of various length, placed medially. Apex of femora with a single long *fes* (Fig. 20A–C). Abdominal segments I–VII with six setae dorsally, variable in length: first minute, placed anteromedially; second and fourth minute; third and fifth medium, placed close to posterior margin of the segment; sixth medium, placed below stigma (on segments VI and VII all setae from second to sixth medium). Abdominal segment VIII with four elongated setae dorsally. Each lateral part of abdominal segments I–VII with two medium setae. Ventral parts of abdominal segments I–VIII with four medium setae. Abdominal segment IX with four medium setae ventrally (Fig. 20A–C).

#### Remarks and comparative notes.

This species is distributed in Central Europe, the Balkans, the Caucasus, and the Middle East (Alonso-Zarazaga et al. 2023). It is clearly distinguishable from *R.antirrhini* and the other species of this group by the shape of its rostrum, which in lateral view is almost straight and gradually narrowed from base to apex and not abruptly tapered at the antennal insertion.

#### Biological notes.

*Rhinusaflorum* is a univoltine weevil that inhabits lowlands, hilly slopes, and mountain meadows at elevations up to 2000 m. The host plants are *Linariagenistifolia* and *L.dalmatica* (L.) Mill. Adults emerge in early June following the occurrence of flowering. Copulation occurs shortly thereafter, with the egg-laying period lasting from mid-June until the end of July. Oviposition occurs on the widest part of the developing ovary. During oviposition, females secrete a fluid that fixes the egg to the ovule. Females lay one egg per ovary. Egg deposition triggers a strong proliferative tissue reaction manifested as a solid, conical gall formation that grows inside the flower lumen. The galled tissue is the only resource for larval development. Pupation takes place inside the gall, and adults emerge after approximately two weeks. The adults overwinter in soil and litter close to the host plants (IT, pers. obs.).

##### ﻿*Rhinusalinariae* group

**Adult diagnosis.** Rostrum strongly curved in lateral view; outer margin of tibiae distinctly curved outwards near apex; uncus of metatibiae well developed in both sexes.

### ﻿5) Rhinusa linariae

Taxon classificationAnimaliaColeopteraCurculionidae

(Panzer, 1796)

8FBF961A-DC50-50CA-A826-2C05B88A8FA6

#### Material examined.

7 mature larvae; 7 ♂ and 12 ♀ pupae. Serbia, Didič, ex *Linariavulgaris* galls, 05.07.2017, leg., det. I. Toševski.

#### Description of mature larva

**Figure 21.**
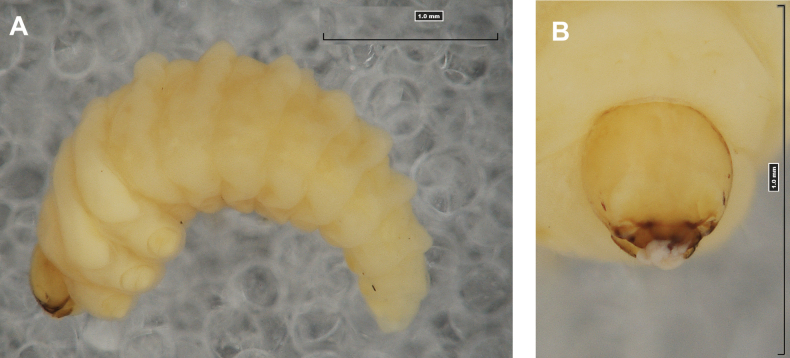
*Rhinusalinariae* (Panzer, 1796) mature larva **A** habitus **B** head, frontal view.

**Figure 22.**
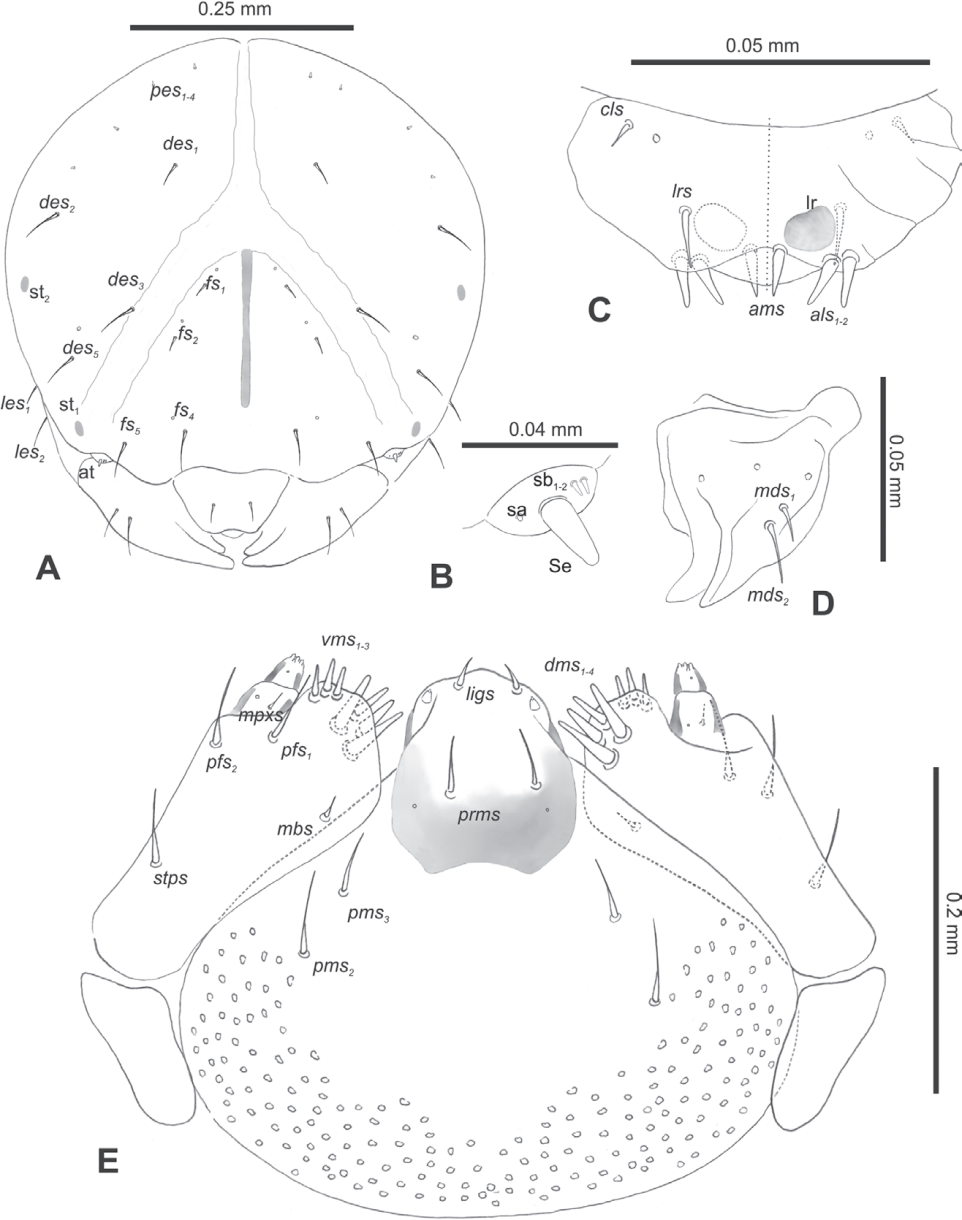
*Rhinusalinariae* (Panzer, 1796) mature larva, head and mouth parts **A** head **B** antenna **C** clypeus and labrum (left side), epipharynx (right side) **D** left mandible **E** maxillolabial complex (schemes). Abbreviations: at–antenna, lr–labral rods, sa–sensillum ampullaceum, sb–sensillum basiconicum, Se–sensorium, st–stemmata, setae: *als*–anterolateral, *ams*–anteromedial, *cls*–clypeal, *des*–dorsal epicranial, *dms*–dorsal malar, *fs*–frontal epicranial, *les*–lateral epicranial, *ligs*–ligular, *lrs*–labral, *mbs*–malar basiventral, *mds*–mandibular dorsal, *mpxs*–maxillary palp, *pes*–postepicranial, *pfs*–palpiferal, *pms*–postmental, *prms*–premental,, *stps*–stipital, *ves*–ventral, *vms*–ventral malar.

**Figure 23.**
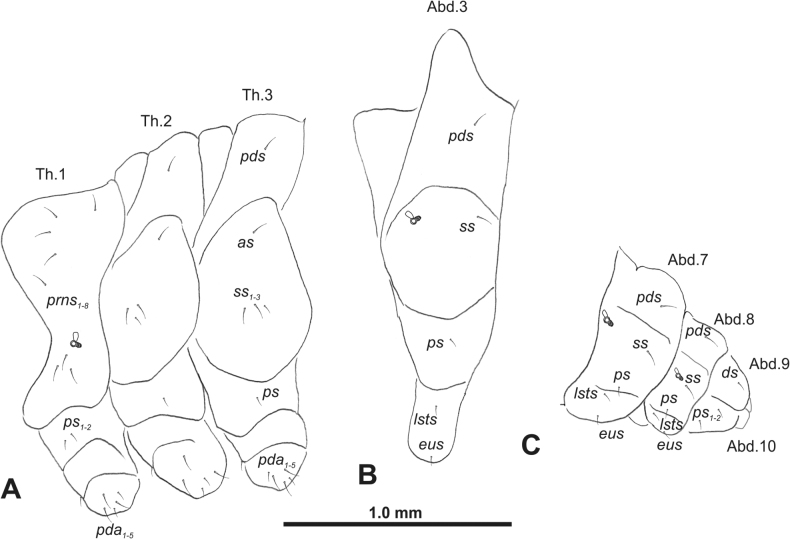
*Rhinusalinariae* (Panzer, 1796) mature larva, habitus **A** lateral view of thoracic segments **B** lateral view of abdominal segment I **C** lateral view of abdominal segments VII–X (schemes). Abbreviations: Th. 1–3–number of thoracic segments, Abd. 1–10–number of abdominal seg, setae: *as*–alar, *ds*–dorsal, *eus*–eusternal, *lsts*–laterosternal, *pda*–pedal, *pds*–postdorsal, *prns*–pronotal, *ss*–spiracular, *ps*–pleural.

**(Figs 21A, B, 22A–E, 23A–C). *Measurements*** (in mm). Body length: 2.33–4.30 (avg. 3.66). The widest place in the body (meso- and metathorax) measures up to 1.16. Head width: 0.46–0.53 (avg. 0.50).

***General*.** Body elongate, slender, strongly curved, rounded in cross section (Fig. 21A). All thoracic segments almost equal in size. Pronotal shield not pigmented. Meso- and metathorax each divided dorsally into two folds (prodorsal fold small, postdorsal prominent). Pedal folds of thoracic segments prominent, conical, and well isolated. Abdominal segments I–IV of similar size, as large as metathorax. Segments V–IX tapering towards posterior body end. Abdominal segments I–VI each divided dorsally into two variously sized folds: prodorsal small, postdorsal folds distinctly larger and much higher than prodorsal folds. Segments VII–IX dorsally undivided. Epipleural folds of segments I–VIII conical. Laterosternal and eusternal folds of segments I–VIII weakly isolated. Abdominal segment X divided into four folds of equal size. Anus situated ventrally, hidden inside ninth segment.

Thoracic and abdominal spiracles unicameral; thoracic spiracles (Figs 21A, 23A) placed laterally close to mesothorax; abdominal spiracles (Figs 21A, 23B, C) placed antero-laterally on segments I–VIII.

***Colouration*.** Light yellow head (Fig. 21B). All thoracic and abdominal segments white (Fig. 21A). Cuticle covered with fine asperities.

***Vestiture*.** Setae on body thin, transparent, different in length (very short or medium).

***Head capsule*** (Figs 21B, 22A). Head suboval, endocarinal line present, reaching to 2/3 length of frons. Frontal sutures on head indistinct, very wide. Two pairs of stemmata in the form of small dark spots (st) placed mediolaterally. *Des_1_* short, located medially; *des_2_* long; long *des_3_* located anteriorly on border of the frontal suture; *des_4_* absent; and *des_5_* long, located anterolaterally (Fig. 22A). *Fs_1_* and *fs_2_* minute, located medially; *fs_3_* absent; *fs_4_* long, located anteriorly; and long *fs_5_* located anterolaterally, close to antenna (Fig. 22A). *Les_1_* and *les_2_* medium. Epicranial area with four *pes*.

***Antennae*** placed distally of the frontal suture, on the inside; membranous and distinctly convex basal article bearing one conical elongate sensorium, plus three sensilla: two basiconica and single ampullacea (Fig. 22B).

***Clypeus and labrum*** (Fig. 22C) completely fused, trapezoidal, 3 × as wide as long, with a single short *cls*, localised posterolaterally; one sensillum posteromedially and single medium piliform *lrs*, located medially; anterior border sinuate. Epipharynx (Fig. 22C) with two finger-like *als* and single piliform *ams*, all relatively elongated; labral rods (lr) rounded, placed close to the anterior border.

***Mouth parts*.** Mandibles (Fig. 22D) bifid, cutting edge with small protuberance; two piliform various in size *mds*, both located, close to lateral border. Maxillolabial complex: maxilla dark sclerotised (Fig. 22E), stipes with one *stps*, two *pfs*, and one minute *mbs*, *stps*, and both *pfs_1–2_* relatively short; mala with four finger-like *dms* variable in length; three piliform *vms*, medium to short in length. Maxillary palpi two-segmented; basal palpomere distinctly wider than distal one; length ratio of basal and distal palpomeres almost 1:1; basal palpomere with short *mpxs* and single sensillum, distal palpomere with a group of five apical sensilla in terminal receptive area. Prementum (Fig. 22E) oval-shaped, with one medium *prms*; ligula with round margin and single medium *ligs*; premental sclerite undefined, weakly sclerotised, without posterior extension. Labial palpi one-segmented, vestigial, visible only under great magnification (40×). Each terminal receptive area with a single apical sensilla. Postmentum (Fig. 22E) with only two *pms*: *pms_1_* absent; medium *pms_2_* located mediolaterally, and relatively short *pms_3_* located anterolaterally; membranous area covered with knobby processes.

***Thorax*.** Prothorax (Fig. 23A) with eigth medium *prns*, dorsal sclerite weakly visible; two medium *ps*; and single short *eus*. Mesothorax (Fig. 23A) with one medium *pds*; one medium *as*; two medium and single minute *ss*; and one short *ps*. Chaetotaxy of metathorax (Fig. 23A) almost identical to that of mesothorax. Each pedal area of thoracic segments with five various in size *pda*.

***Abdomen*.** Segments I–VIII (Fig. 23B, C) with one medium *pds*; one medium *ss*; one short *ps*; one short *lsts*; and one short *eus*. Abdominal segment IX (Fig. 23C) with one medium *ds* and two short *ps*.

#### Description of pupa

**Figure 24.**
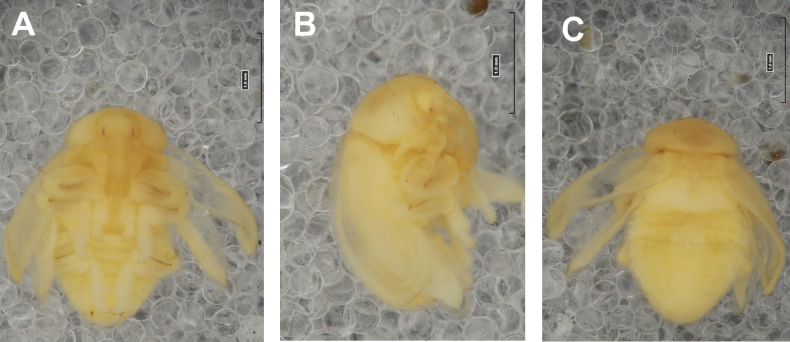
*Rhinusalinariae* (Panzer, 1796) pupa habitus **A** ventral view **B** lateral view **C** dorsal view.

**Figure 25.**
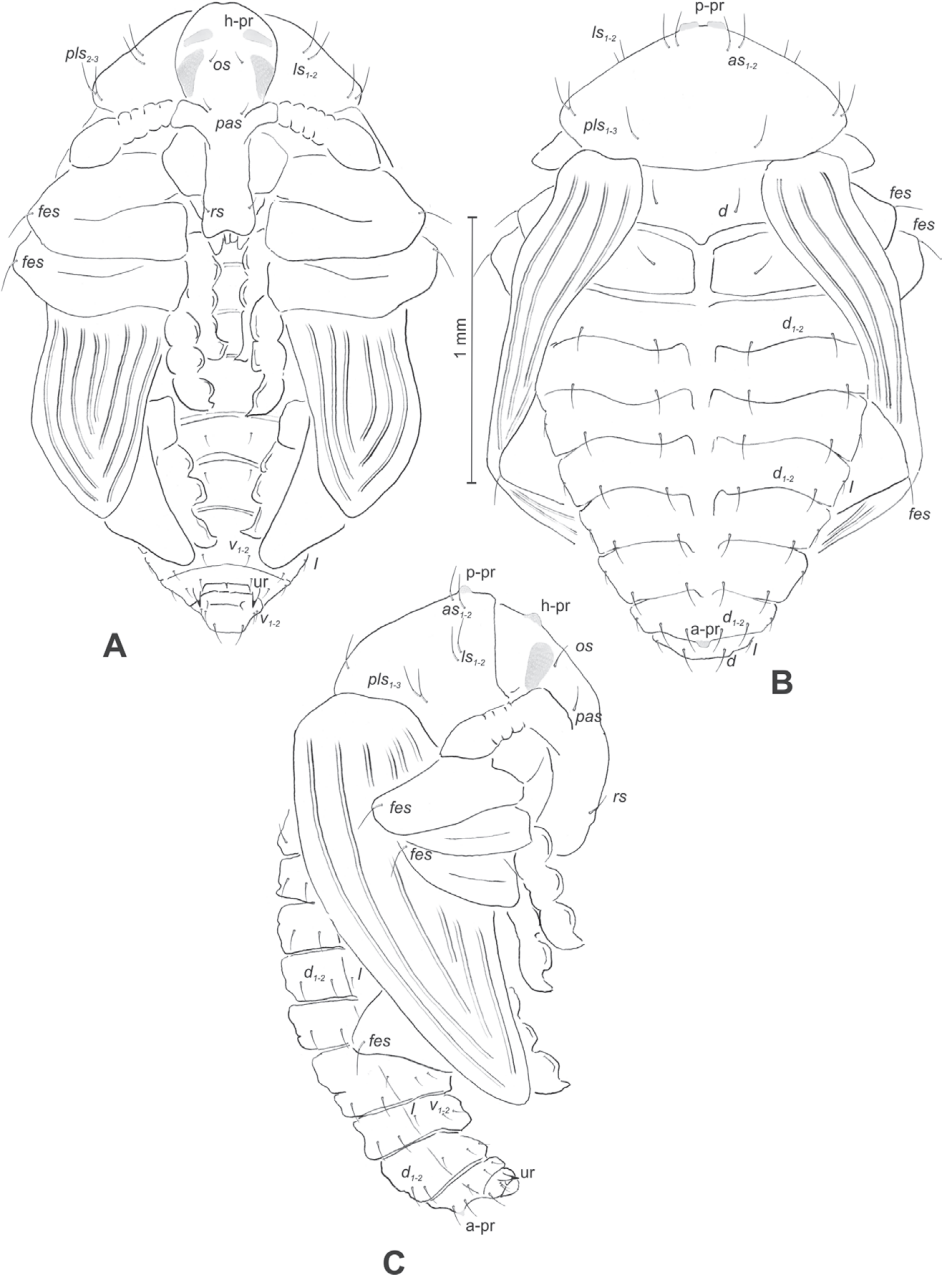
*Rhinusalinariae* (Panzer, 1796) pupa habitus **A** ventral view **B** dorsal view **C** lateral view (schemes). Abbreviations: a–pr–abdominal protuberances, h–pr–head protuberances, p–pr–pronotal protuberances, ur–urogomphi, setae: *as*–apical, *d*–dorsal, *fes*–femoral, *l*, *ls*–lateral, *os*–orbital, *pas*–postantennal, *pls*–posterolateral, *rs*–rostral, *v*–ventral.

**(Figs 24A–C, 25A–C). *Measurements*** (in mm). Body length: 1.66–2.66 (avg. 2.10); body width: 1.40–1.93 (avg. 1.60); thorax width: 0.90–1.18 (avg. 1.05); rostrum length: up to 0.66 on both ♀ and ♂.

***Body*.** Integument white, moderately stout slightly curved. Head elongated protuberances (h–pr) present on head above eyes, weakly sclerotised. Rostrum moderately elongated, curved, in male usually only slightly shorter than in female almost 3 × as long as wide, reaching mesocoxae. Pronotum trapezoidal 3 × as wide as long. Pronotal protuberances (p–pr) conical, flattened, indistinct. Meso- and metanotum similar in size. Abdominal segments I–III almost identical in size; segments IV–VII tapering gradually, segment VIII narrow; segment IX reduced. Abdominal segment VIII dorsally with very small, rounded, weakly sclerotised abdominal protuberance (a–pr). Urogomphi (ur) very short, ending with sclerotised, sharp apexes (Fig. 24A–C).

***Chaetotaxy*.** Well developed, setae variable in length, transparent. Head with one short *os* and short *pas*. Rostrum with a single short *rs* (Fig. 25A). Pronotum with two *as*, two *ls*, and three elongated, equal-in-length *pls*. Dorsal parts of meso- and metathorax with a single medium-length seta, placed medially. Apex of femora with a single long *fes* (Fig. 25A–C). Abdominal segments I–VII with two, equal-in-length setae dorsally: first placed posteromedially, second posterolaterally. Abdominal segment VIII with a single elongated seta medially. Each lateral part of abdominal segments I–VII with a single short seta. Ventral parts of abdominal segments I–VIII with two short setae. Abdominal segment IX with two short setae ventrally (Fig. 25A–C).

#### Remarks and comparative notes.

This species is widely distributed in all of Europe, Turkey, Kazakhstan, and western Siberia (Alonso-Zarazaga et al. 2023). It was introduced in North America, where it was approved for release for biological control of invasive toadflaxes (*Linaria* spp.) in Canada in 1995 and 1996 (Sing et al. 2016). Afterwards, a population was established at sites in British Columbia and Colorado (Sing et al. 2016; DiGirolomo et al. 2019). In Europe, the adults of this species are distinguishable from all the other species of *Rhinusa* by the shape of the rostrum, which is strongly curved in lateral view, and the shape of the tibiae, with the uncus of the metatibiae of the same length in both sexes and with the outer margin distinctly curved outwards apically (Caldara and Toševski 2019).

#### Biological notes.

*Rhinusalinariae* is a univoltine root galling weevil. Gall induction and larval development are mainly recorded on *Linariavulgaris* and rarely on *L.genistifolia* in Southeastern Europe. Adults emerge in early spring, feed, and copulate on top of the young toadflax shoots. During oviposition, females glue eggs onto toadflax roots or, rarely, below root crowns with oviposition fluid. Eggs are laid singly or in small groups. Shortly after, oviposition triggers cell proliferation, which entwists the egg, forming a round gall. Larvae feed on galled root tissue through three instars. Pupation occurs in galls, while new adults emerge in mid- to late summer or rarely stay inside galls during winter, overwintering in soil or in plant litter close to their host plant. Twenty-five years ago, *R.linariae* was introduced as a biological control agent for invasive toadflaxes in Canada and the USA, where it was recently confirmed as established only in British Columbia. However, the current populations are still too small to have a significant biological impact (Sing et al. 2016; DiGirolomo et al. 2019).

##### ﻿*Rhinusapilosa* group

**Adult diagnosis.** Dorsal vestiture composed of very long seta-like scales, 20–40 × longer than wide; rostrum in lateral view strongly curved; eyes strongly convex; uncus of metatibiae well developed in both sexes; body of spermatheca globose at apex.

### ﻿6) Rhinusa pilosa

Taxon classificationAnimaliaColeopteraCurculionidae

(Gyllenhal, 1838)

51BF0A4C-9B0F-5889-83AA-BD981C67A2A9

#### Material examined.

4 mature larvae; 3 ♂ and 5 ♀ pupae. Serbia, Zemun, ex *Linariavulgaris* galls, 01.06.2018, leg., det. I. Toševski.

#### Description of mature larva

**Figure 26.**
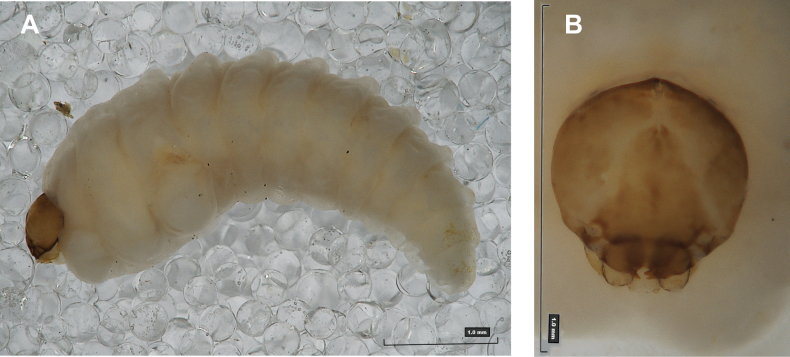
*Rhinusapilosa* (Gyllenhal, 1838) mature larva **A** habitus **B** head, frontal view.

**Figure 27.**
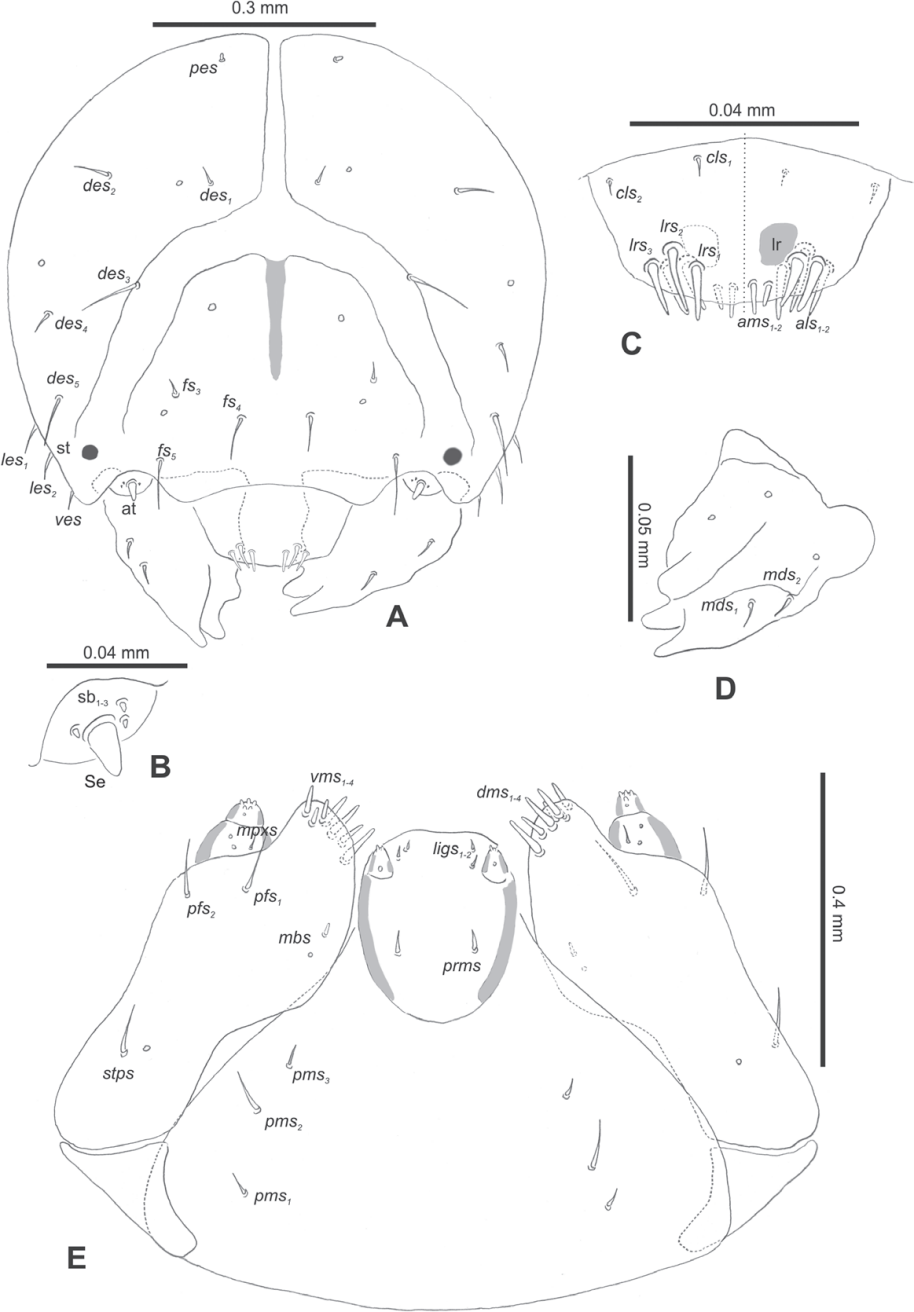
*Rhinusapilosa* (Gyllenhal, 1838) mature larva, head and mouth parts **A** head **B** antenna **C** clypeus and labrum (left side), epipharynx (right side) **D** left mandible **E** maxillolabial complex (schemes). Abbreviations: at–antenna, lr–labral rods, sb–sensillum basiconicum, Se–sensorium, st–stemmata, setae: *als*–anterolateral, *ams*–anteromedial, *cls*–clypeal, *des*–dorsal epicranial, *dms*–dorsal malar, *fs*–frontal epicranial, *les*–lateral epicranial, *ligs*–ligular, *lrs*–labral, *mbs*–malar basiventral, *mds*–mandibular dorsal, *mpxs*–maxillary palp, *pes*–postepicranial, *pfs*–palpiferal, *pms*–postmental, *prms*–premental, *stps*–stipital, *ves*–ventral, *vms*–ventral malar.

**Figure 28.**
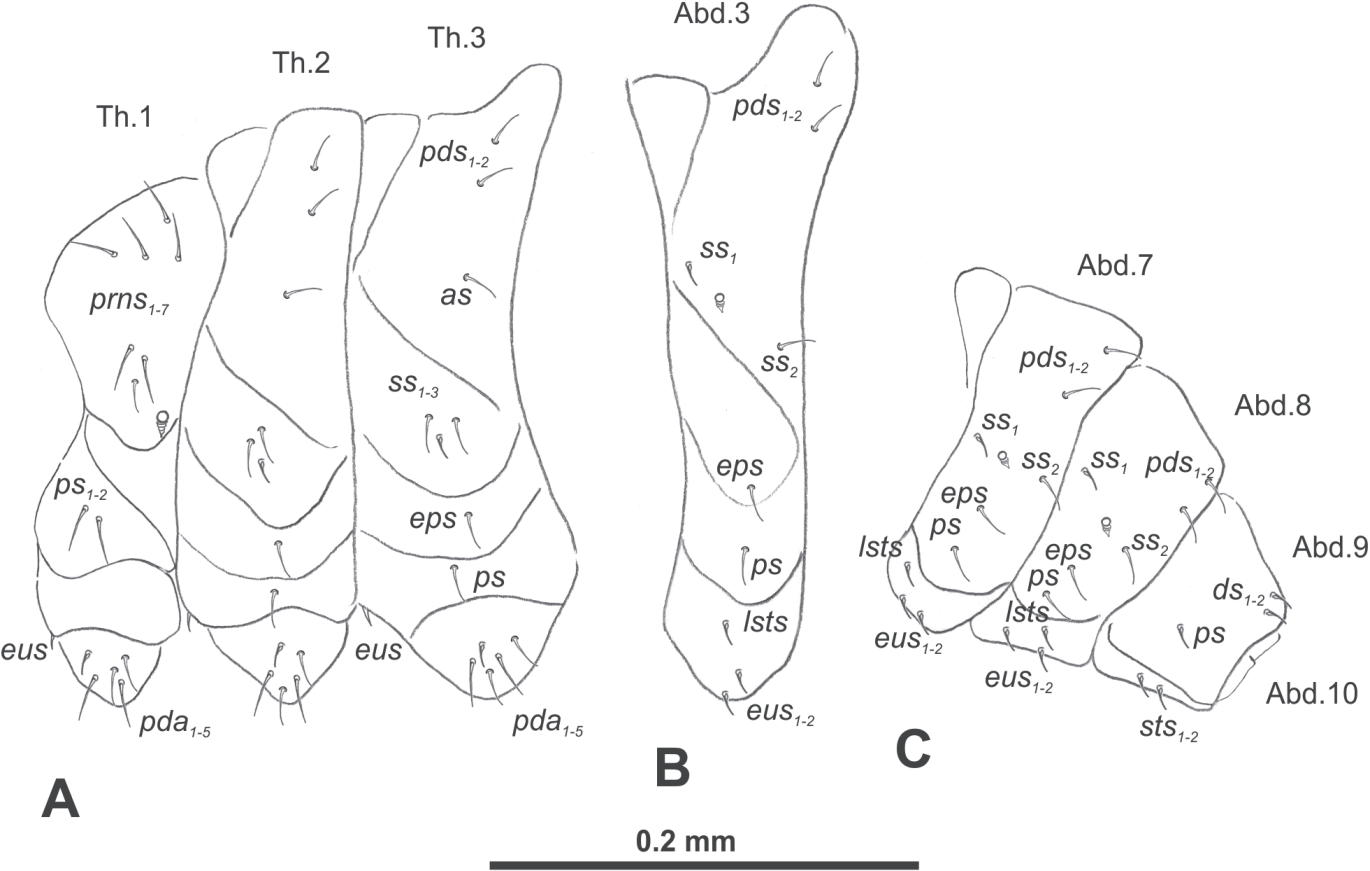
*Rhinusapilosa* (Gyllenhal, 1838) mature larva, habitus **A** lateral view of thoracic segments **B** lateral view of abdominal segment I **C** lateral view of abdominal segments VII–X (schemes). Abbreviations: Th. 1–3–number of thoracic segments, Abd. 1–10–number of abdominal seg, setae: *as*–alar, *ds*–dorsal, *eps*–epipleural, *eus*–eusternal, *lsts*–laterosternal, *pda*–pedal, *pds*–postdorsal, *prns*–pronotal, *ss*–spiracular, *ps*–pleural, *sts*–sternal.

**(Figs 26A, B, 27A–E, 28A–C). *Measurements*** (in mm). Body length: 4.00–5.75 (avg. 4.25). The widest place in the body (meso- and metathorax) measures up to 1.50. Head width: 0.60–0.68 (avg. 0.65).

***General*.** Body elongate, slightly curved, rounded in cross section (Fig. 26A). Prothorax slightly smaller than mesothorax, pronotal shield not pigmented. Meso- and metathorax equal in size; each divided dorsally into two folds (prodorsal fold distinctly smaller than postdorsal fold); postdorsal fold of metathorax conical. Pedal folds of thoracic segments isolated, prominent. Abdominal segments I–VI of similar size, next segments tapering towards posterior body end. Abdominal segments I–VII each divided dorsally into two folds of almost identical size; postdorsal folds of segments I–VI higher than prodorsal folds. Segments VIII and IX dorsally undivided. Epipleural folds of segments I–VIII conical. Laterosternal and eusternal folds of segments I–VIII weakly isolated. Abdominal segment X divided into four folds of equal size. Anus situated ventrally, almost completely covered with the ninth abdominal segment.

All spiracles unicameral; thoracic spiracles (Fig. 26A) placed laterally close to mesothorax; abdominal spiracles (Fig. 26A) placed anteromedially on segments I–VIII.

***Colouration*.** Light yellow to dark yellow head, medial parts of epicranium less sclerotised (Fig. 26B). All thoracic and abdominal segments whitish (Fig. 26A). Cuticle covered with asperities.

***Vestiture*.** Setae on body thin, transparent, different in length (very short or medium).

***Head capsule*** (Figs 26B, 27A). Head wide, endocarinal line present, reaching to 2/3 length of frons. Frontal sutures on head indistinct, very wide. Single pair of stemmata in the form of small black spots (st) close to the end of the frontal suture. *Des_1_* short, located in middle part of epicranium; medium *des_2_*; long *des_3_* located anteriorly on epicranium close to the border with the frontal suture; *des_4_* minute; and *des_5_* long, located anterolaterally above stemma (Fig. 27A). *Fs_1_* and *fs_2_* absent; *fs_3_* minute; *fs_4_* medium, located anteriorly; and long *fs_5_* located anterolaterally, close to antenna (Fig. 27A). *Les_1_* and *les_2_* medium; single short *ves*. Epicranial area with a single *pes*.

***Antennae*** placed distally of the frontal suture, on the inside; membranous and distinctly convex basal article bearing one conical relatively short sensorium, plus three sensilla basiconica (Fig. 27B).

***Clypeus and labrum*** (Fig. 27C) completely fused, trapezoidal, 3 × as wide as long, with two short *cls*, localised posteriorly three medium piliform *lrs*, located anteromedially. Epipharynx (Fig. 27C) with two finger-like elongated *als*; and two piliform *ams*, variable in length; labral rods (lr) indistinct, close to oval-shape; anterior border almost straight.

***Mouth parts*.** Mandibles (Fig. 27D) bifid, cutting edge with blunt additional teeth; two short piliform *mds*, close to lateral border. Maxillolabial complex: maxilla more sclerotised than labium (Fig. 27E) stipes with one *stps*, two *pfs* and one very short *mbs* and one sensillum, *stps* and both *pfs_1–2_* relatively short; mala with four finger-like *dms* variable in length; four piliform *vms*, medium to short in length. Maxillary palpi two-segmented; basal palpomere distinctly wider than distal one; length ratio of basal and distal palpomeres almost 1:2; basal palpomere with short *mpxs* and two sensilla, distal palpomere with a group of five apical sensilla in terminal receptive area. Prementum (Fig. 27E) oval-shaped, with one short *prms*; ligula with round margin and two minute *ligs*; premental sclerite vestigial, only lateral parts highly sclerotised, posterior extension absent. Labial palpi one-segmented; palpi very small, with a single pore, and a group of three or four apical sensilla (ampullacea) on terminal receptive area; surface of labium smooth. Postmentum (Fig. 27E) with three *pms*, short *pms_1_* located posteromedially, medium *pms_2_* located mediolaterally, and short *pms_3_* located anterolaterally; membranous area smooth.

***Thorax*.** Prothorax (Fig. 28A) with seven elongated to medium *prns*; two medium *ps*; and single short *eus*. Mesothorax (Fig. 28A) without *prs*; with two medium *pds*; one medium *as*; three *ss* (two medium and one short); one medium *eps*; one medium *ps*; and single minute *eus*. Chaetotaxy of metathorax (Fig. 28A) almost identical to that of mesothorax. Each pedal area of thoracic segments with five *pda* of various length.

***Abdomen*.** Segments I–VIII (Fig. 28B, C) without *prs*; with two medium *pds*; one minute and one medium *ss*; one medium *eps*; one medium *ps*; one short *lsts*; and two minute *eus*. Abdominal segment IX (Fig. 28C) with two minute *ds*; one minute *ps*; and two minute *sts*.

#### Description of pupa

**Figure 29.**
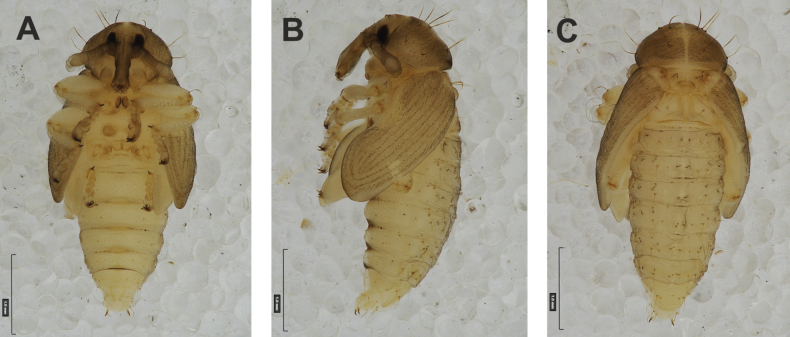
*Rhinusapilosa* (Gyllenhal, 1838) pupa habitus **A** ventral view **B** lateral view **C** dorsal view.

**Figure 30.**
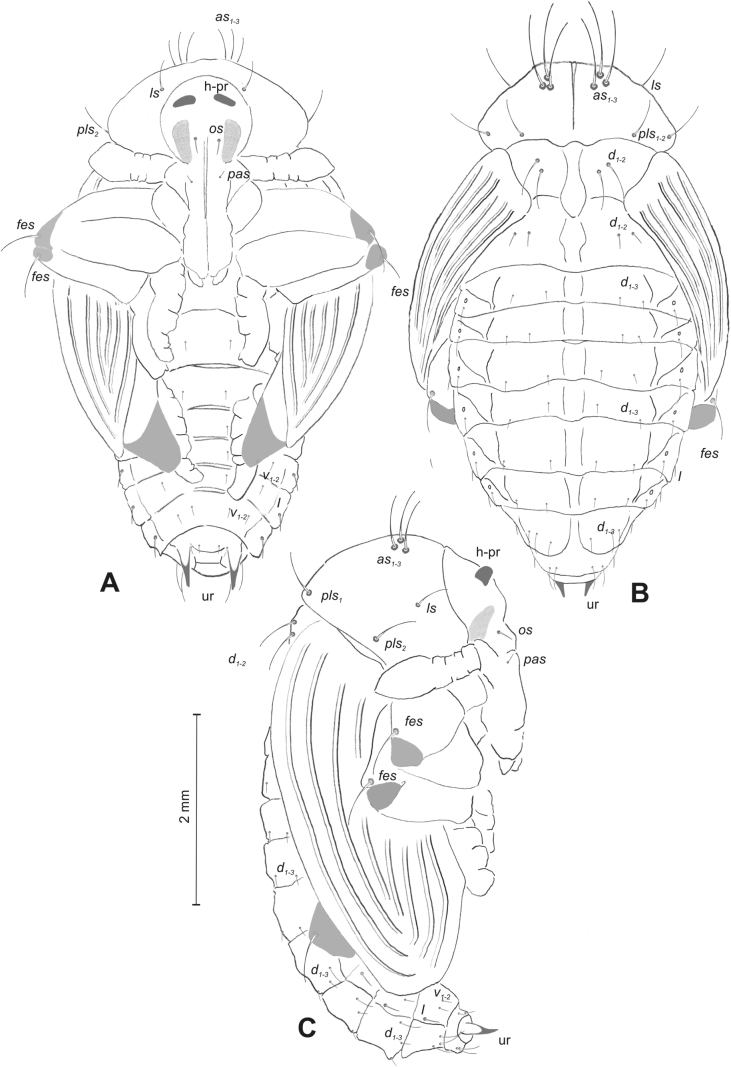
*Rhinusapilosa* (Gyllenhal, 1838) pupa habitus **A** ventral view **B** dorsal view **C** lateral view (schemes). Abbreviations: h–pr–head protuberances, ur–urogomphi, setae: *as*–apical, *d*–dorsal, *fes*–femoral, *l*, *ls*–lateral, *os*–orbital, *pas*–postantennal, *pls*–posterolateral, *v*–ventral.

**(Figs 29A–C, 30A–C). *Measurements*** (in mm). Body length: 2.86–3.75 (avg. 3.25); body width: 1.90–2.25 (avg. 2.00); thorax width: 1.10–1.35 (avg. 1.25); rostrum length: up to 0.40 ♂, ♀.

***Body*.** Integument white, with some parts dark sclerotised; moderately elongated, curved. Head protuberances (h–pr) elongated. Rostrum rather stout, on both sexes almost 2.3 × as long as wide, extended only to procoxae. Pronotum trapezoidal 3 × as wide as long. Pronotal protuberances (p–pr) absent. Meso- and metanotum similar in size. Abdominal segments I–VI almost identical in size; segment VII semicircular; segment VIII narrow; segment IX reduced. Abdominal protuberances (a–pr) absent. Urogomphi (ur) medium-sized, ending with sclerotised, sharp apexes (Fig. 29A–C).

***Chaetotaxy*.** Well developed, setae minute to elongated. Minute and medium setae transparent, elongated setae basally brown, apically transparent. Head with one minute *os* (Fig. 30A). Rostrum with a single minute *pas*. Pronotum with three *as*, single *ls*, and two *pls*; all pronotal setae almost equally in length, prominent, basally brownish, apically transparent. Dorsal parts of meso- and metathorax with two identical in length setae, placed medially. Setae of mesothorax as long as those on pronotum. Apex of femora with a single long *fes* (Fig. 30A–C). Abdominal segments I–VI with three setae: first and second minute placed anteromedially, third medium placed below stigma. Abdominal segments VII with three elongated setae dorsally and segment VIII with two elongated setae dorsally. Each lateral part of abdominal segments I–VII with a single medium seta. Ventral parts of abdominal segments I–VIII with two minute setae. Abdominal segment IX with two minute setae ventrally (Fig. 30A–C).

#### Remarks and comparative notes.

This species is distributed in northern and central Europe and in the Balkans (Alonso-Zarazaga et al. 2023). It is distinguishable from other species of the *R.pilosa* group by the rostrum being markedly bent at the level of antennal insertion in both sexes. It is a unique species in the group in being distributed northwards in the western Palaearctic, associated only with *L.vulgaris* as a host plant. All three species of this group differ from the other species of *Rhinusa* by the very long, hair-like scales of the dorsal vestiture.

#### Biological notes.

*Rhinusapilosa* is a shoot-galling weevil associated with *L.vulgaris* for larval development. Adults become active in early spring, and their appearance after winter hibernation coincides with the intensive shoot growth of their host plant. Females oviposit in the upper part of the young, growing shoots of *L.vulgaris*. Females oviposit three to six eggs, but the number of ovipositions has been observed to exceed 17 per shoot. Oviposition provokes the induction of a globose or elyptical gall on the apical part of the stem. Larvae feed and complete development within the induced galls. Pupation is also completed within the gall. Eclosed adults intensively feed on gall tissue, after which they leave the gall and enter into summer aestivation within the soil litter or soil cracks. In late autumn, adults are briefly active, feeding on young *L.vulgaris* shoots before entering diapause, sheltering close to the host plant. The biology of *R.pilosa* is described in detail by Gassmann et al. (2014).

### ﻿7) Rhinusa rara

Taxon classificationAnimaliaColeopteraCurculionidae

Toševski & Caldara, 2015

F6C554CB-5005-52FC-86C7-F2889C7D0749

#### Material examined.

2 mature larvae, 26.04.2014; 1♂ and 3♀ pupae, 10.05.2014; 8 mature larvae; 2♂ and 2♀ pupae, 12.05.2014, ex *Linariadalmatica*, Serbia, Staničenje, Pirot, leg., det. I. Toševski.

#### Description of mature larva

**Figure 31.**
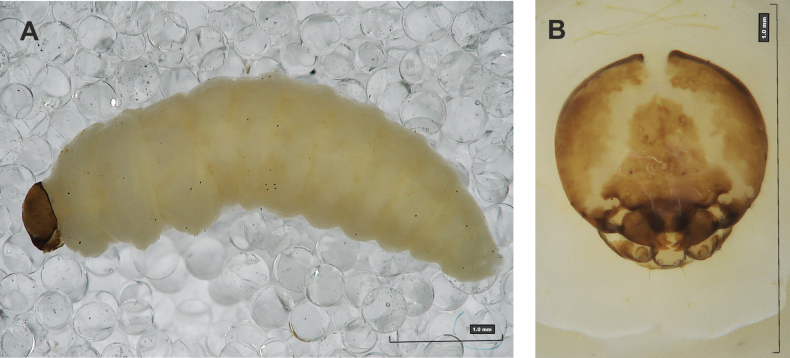
*Rhinusarara* Toševski & Caldara, 2015 mature larva **A** habitus **B** head, frontal view.

**Figure 32.**
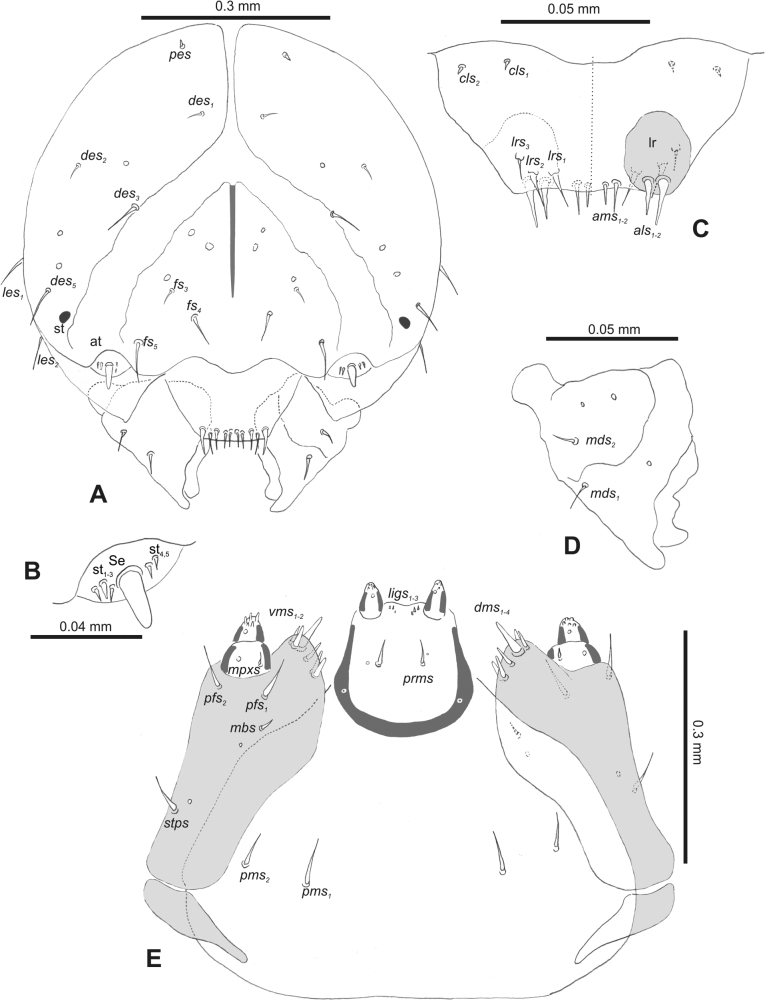
*Rhinusarara* Toševski & Caldara, 2015 mature larva, head and mouth parts **A** head **B** antenna **C** clypeus and labrum (left side), epipharynx (right side) **D** left mandible **E** maxillolabial complex (schemes). Abbreviations: at–antenna, lr–labral rods, st–sensillum styloconicum, Se–sensorium, st–stemmata, setae: *als*–anterolateral, *ams*–anteromedial, *cls*–clypeal, *des*–dorsal epicranial, *dms*–dorsal malar, *fs*–frontal epicranial, *les*–lateral epicranial, *ligs*–ligular, *lrs*–labral, *mbs*–malar basiventral, *mds*–mandibular dorsal, *mpxs*–maxillary palp, *pes*–postepicranial, *pfs*–palpiferal, *pms*–postmental, *prms*–premental, *stps*–stipital, *vms*–ventral malar.

**Figure 33.**
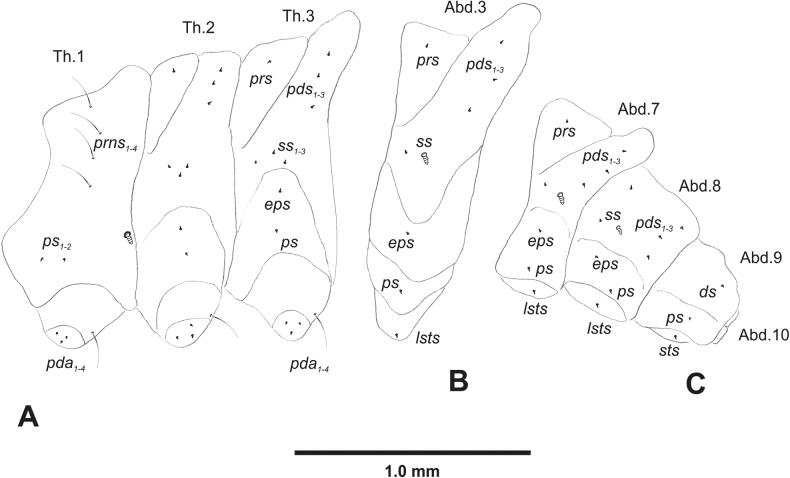
*Rhinusarara* Toševski, Caldara, 2015 mature larva, habitus **A** lateral view of thoracic segments **B** lateral view of abdominal segment I **C** lateral view of abdominal segments VII–X (schemes). Abbreviations: Th. 1–3–number of thoracic segments, Abd. 1–10–number of abdominal seg, setae: *ds*–dorsal, *eps*–epipleural, *pda*–pedal, *pds*–postdorsal, *prns*–pronotal, *prs*–prodorsal, *ss*–spiracular, *ps*–pleural, *sts*–sternal.

**(Figs 31A, B, 32A–E, 33A–C). *Measurements*** (in mm). Body length: 3.00–4.25 (avg. 3.60). The widest place in the body (meso- and metathorax) measures up to 1.25. Head width: 0.55–0.60 (avg. 0.56).

***General*.** Body elongate, slender, distinctly curved, rounded in cross section (Fig. 31A). All thoracic segments almost equal in size. Meso- and metathorax each divided dorsally into two folds (prodorsal fold distinctly smaller than postdorsal fold). Pedal folds of thoracic segments isolated, conical, prominent. Abdominal segments I–VI of similar size, next segments tapering towards posterior body end. Abdominal segments I–VII each divided dorsally into two folds: prodorsal fold slightly smaller than postdorsal, which form conical, prominent protuberances apically. Segments VIII and IX dorsally undivided. Epipleural folds of segments I–VIII conical. Laterosternal and eusternal folds of segments I–VIII conical, weakly isolated. Abdominal segment X (almost completely hidden in previous segment) divided into four folds of equal size. Anus situated ventrally.

All spiracles unicameral; thoracic spiracles (Fig. 31A) placed laterally close to mesothorax; abdominal spiracles (Fig. 31A) placed medio-laterally on segments I–VIII.

***Colouration*.** Light yellow to yellow head, medial parts of epicranium less sclerotised (Fig. 31B). All thoracic and abdominal segments white (Fig. 31A). Cuticle covered with asperities.

***Vestiture*.** Setae on body thin, yellowish, different in length (very short or medium).

***Head capsule*** (Figs 31B, 32A). Head wide, endocarinal line present, reaching to 2/3 length of frons. Frontal sutures on head very wide, indistinct. Single pair of stemmata in the form of small black spots (st) laterally to the end of the frontal suture. *Des_1_* short; *des_2_* short, located in lateral part of epicranium; long *des_3_* located anteriorly on epicranium on border of the frontal suture; *des_4_* absent; and *des_5_* long, located anterolaterally above stemma (Fig. 32A). *Fs_1_* and *fs_2_* absent; *fs_3_* minute, located medially; *fs_4_* long, located anteriorly; and long *fs_5_* located anterolaterally, close to antenna (Fig. 32A). *Les_1_* and *les_2_* medium. Epicranial area with a single *pes*.

***Antennae*** placed distally of the frontal suture, on the inside; membranous and distinctly convex basal article bearing one conical elongate sensorium, plus five sensilla styloconica (Fig. 32B).

***Clypeus and labrum*** (Fig. 32C) completely fused, trapezoidal, 2.7 × as wide as long, with two minute *cls*, localised posterolaterally; three piliform *lrs*, various long; *lrs_1_* and *lrs_2_* medium, located anteromedially, and *lrs_3_* short, located laterally; anterior border almost straight. Epipharynx (Fig. 32C) with two finger-like *als*, variable in length and two *ams* variable in length; labral rods (lr) indistinct, rounded; anterior border sinuate.

***Mouth parts*.** Mandibles (Fig. 32D) bifid, cutting edge with additional protuberance; two medium piliform *mds*, both located close to lateral border. Maxillolabial complex: maxilla more sclerotised than labium (Fig. 32E) stipes with one *stps*, two *pfs* and one very short *mbs*, *stps* and both *pfs_1–2_* relatively short; mala with four piliform *dms* variable in length; two short piliform *vms*. Maxillary palpi two-segmented; basal palpomere distinctly wider and slightly longer than distal one; basal palpomere with short *mpxs* and single sensillum, distal palpomere with a group of four or five apical sensilla in terminal receptive area. Prementum (Fig. 32E) close to oval-shaped, with one short *prms*; ligula with slightly sinuate margin and three minute *ligs*; premental sclerite sclerotised U-shaped. Labial palpi one-segmented; each palp with a single pore, and a group of three or four apical sensilla (ampullacea) on terminal receptive area; surface of labium smooth. Postmentum (Fig. 32E) with only two *pms*, medium *pms_1_* located medially and short *pms_2_* located laterally, *pms_3_* absent; membranous area smooth.

***Thorax*.** Only pronotal and single pedal setae elongated, rest of thoracic minute, feebly visible. Prothorax (Fig. 33A) with four *prns* and two *ps*. Mesothorax (Fig. 33A) with one *prs*, three *pds*; three *ss*; one *eps* and one *ps*. Chaetotaxy of metathorax (Fig. 33A) almost identical to that of mesothorax. Each pedal area of thoracic segments with three minute and one elongated *pda*.

***Abdomen*.** All abdominal setae minute, feebly visible. Segments I–VIII (Fig. 33B, C) with one *prs*; three *pds*; one *ss*; one *eps*; one *ps* and one *lsts*. Abdominal segment IX (Fig. 33C) with a single *ds*, single *ps*, and single *sts*.

#### Description of pupa

**Figure 34.**
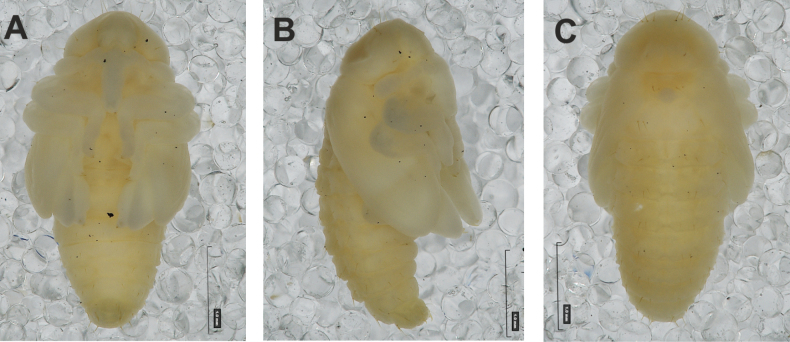
*Rhinusarara* Toševski & Caldara, 2015 pupa habitus **A** ventral view **B** lateral view **C** dorsal view.

**Figure 35.**
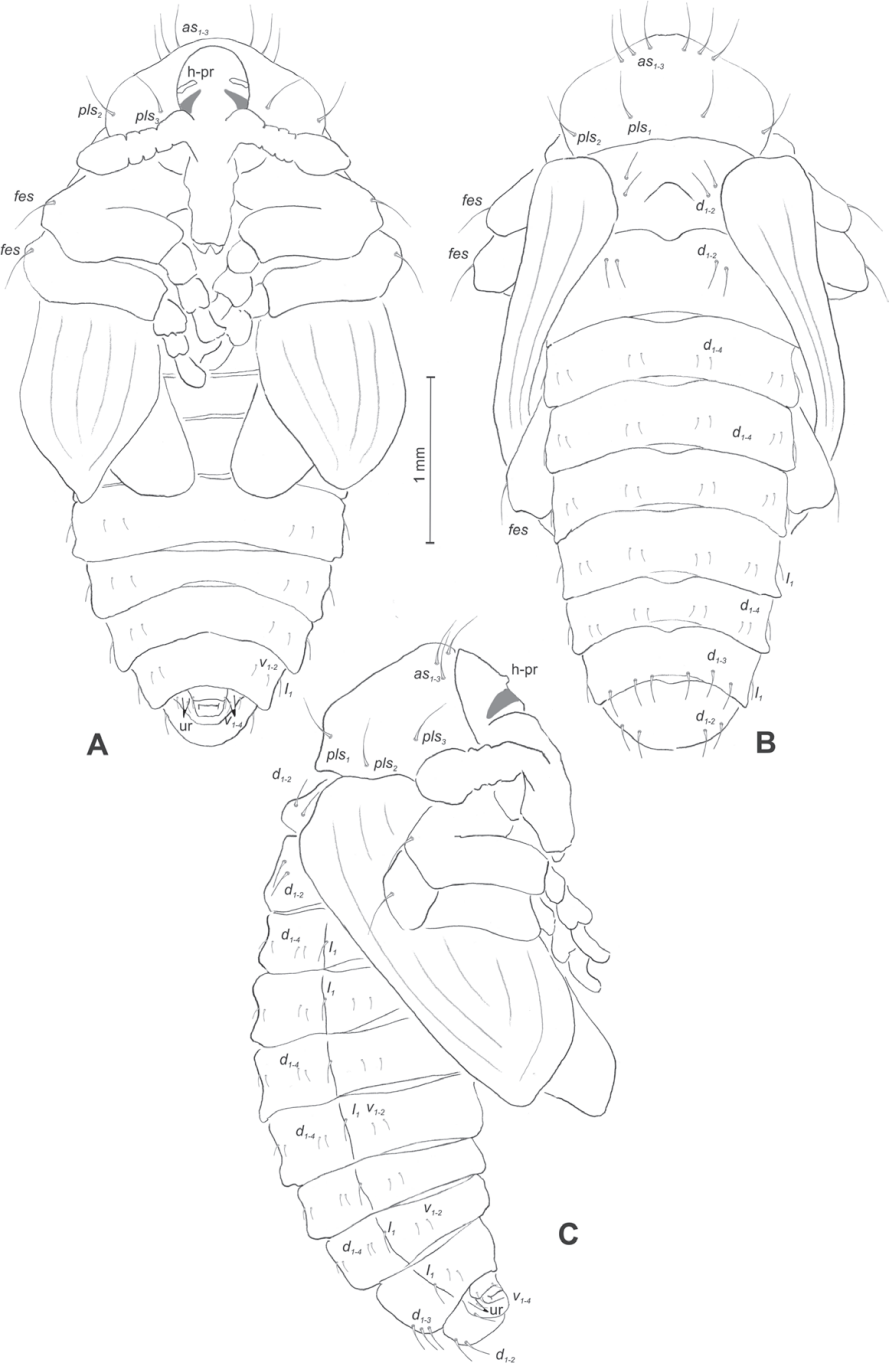
*Rhinusarara* Toševski & Caldara, 2015 pupa habitus **A** ventral view **B** dorsal view **C** lateral view (schemes). Abbreviations: h–pr–head protuberances, ur–urogomphi, setae: *as*–apical, *d*–dorsal, *fes*–femoral, *l*–lateral, *pls*–posterolateral, *v*–ventral.

**(Figs 34A–C, 35A–C). *Measurements*** (in mm). Body length: 3.35–3.85 (avg. 3.75); body width: 1.60–2.10 (avg. 1.75); thorax width: 1.05–1.30 (avg. 1.20); rostrum length: up to 1.50 for both sexes.

***Body*.** Integument white, moderately elongated, curved. Head protuberances (h–pr) above eyes present. Rostrum rather short, in male usually only slightly shorter than in female almost 2.5 × as long as wide, reaching mesocoxae. Pronotum trapezoidal 2 × as wide as long. Pronotal protuberances (p–pr) absent. Meso- and metanotum similar in size. Abdominal segments I–VI almost identical in size; segment VII semicircular; segment VIII narrow; segment IX reduced. Urogomphi (ur) short, ending with sclerotised, sharp apexes (Fig. 34A–C).

***Chaetotaxy*.** Well developed, setae short to elongated, short setae transparent, elongated brown. Head and rostrum without seta (Fig. 35A). Pronotum with three *as*, and three *pls* almost equally in length. Dorsal parts of meso- and metathorax with two setae of similar length, placed medially. Apex of femora with a single long *fes* (Fig. 35A–C). Abdominal segments I–VI with four short setae dorsally, all placed close to posterior margin. Abdominal segment VII with three elongated setae dorsally. Abdominal segment VIII with two elongated setae dorsally. Each lateral part of abdominal segments I–VII with a single short seta. Ventral parts of abdominal segments I–VIII with two short setae. Abdominal segment IX with two short setae ventrally (Fig. 35A–C).

#### Remarks and comparative notes.

This species is very restricted in its distribution and scarce. It is known only from calcareous regions in Serbia (Sićevo Gorge between the towns of Niš and Pirot), Hungary (Balaton), southern Slovakia (Šturovo), southern Czechia (Znojmo), Austria (Wien) and southern Russia (Toševski et al. 2015). It differs from the other European species of the *R.pilosa* group in its evenly curved rostrum in lateral view in both sexes (vs. abruptly narrowed and bent along the dorsal margin), almost flat pronotum and elytra (vs. moderately convex), and integument of adults covered with recumbent hair-like scales (vs. suberect hair-like scales).

#### Biological notes.

The biology of *R.rara* is similar to that of *R.pilosa*. The adults become active in the field very early, often in mid-February. The adults are hidden inside the rosette of the host plant, *L.genistifolia* or *L.dalmatica*. The females oviposit at the base of young, growing shoots. Induced galls are usually large, partly hidden below the soil surface. Usually, ~ 10 eggs are laid per shoot, but some shoots can be used for 20 or more ovipositions. The biology of *R.rara* is described in detail by Toševski et al. (2015).

##### ﻿*Rhinusaherbarum* group

**Adult diagnosis.** Rostrum in lateral view straight; elytra rectangular and only slightly wider than pronotum; third tarsomere weakly bilobed and slightly wider than second tarsomere; femora unarmed; body of penis short and in lateral view with sides distinctly widening in apical part.

### ﻿8) Rhinusa herbarum

Taxon classificationAnimaliaColeopteraCurculionidae

(H. Brisout de Barneville, 1862)

569C36AF-E2B0-5F28-8B2C-EB427CA0801D

#### Material examined.

20 mature larvae; 6 ♂ and 6 ♀ pupae. Serbia, Sredrievo, ex *Kickxiaelatine* (L.) Dumort., 15.08.2017, leg., det. I. Toševski.

#### Description of mature larva

**Figure 36.**
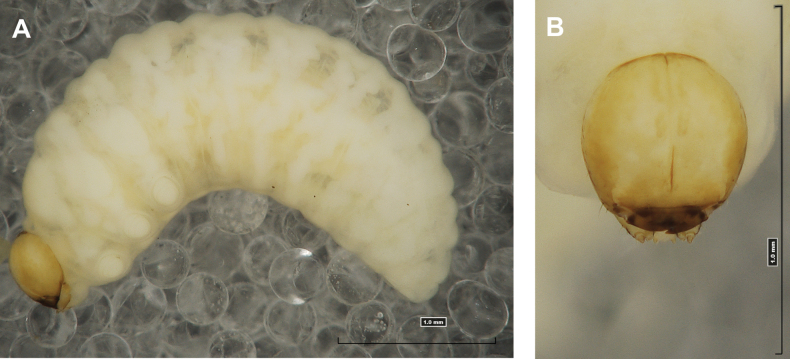
*Rhinusaherbarum* (H. Brisout de Barneville, 1862) mature larva **A** habitus **B** head, frontal view.

**Figure 37.**
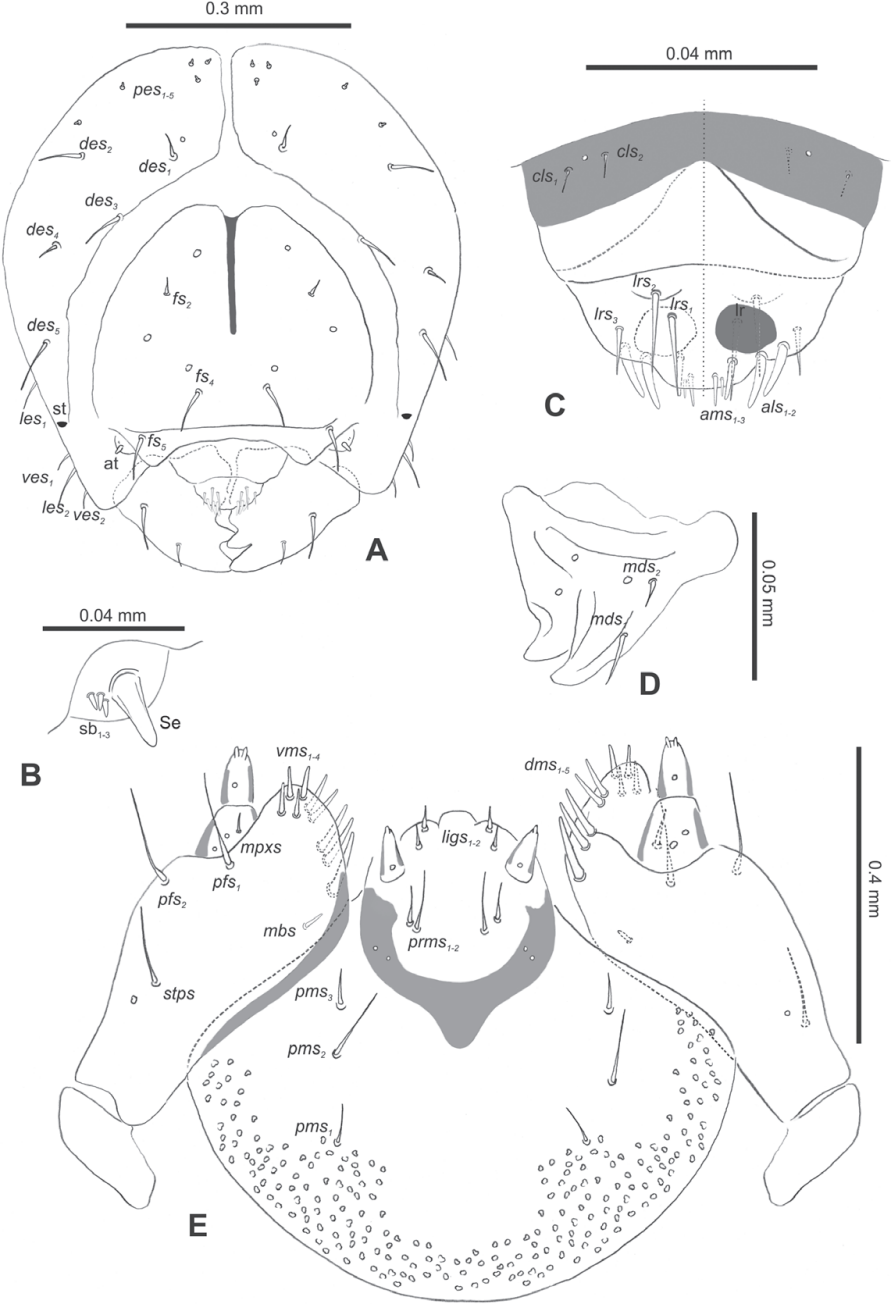
*Rhinusaherbarum* (H. Brisout de Barneville, 1862) mature larva, head and mouth parts **A** head **B** antenna **C** clypeus and labrum (left side), epipharynx (right side) **D** left mandible **E** maxillolabial complex (schemes). Abbreviations: at–antenna, lr–labral rods, sb–sensillum basiconicum, Se–sensorium, st–stemmata, setae: *als*–anterolateral, *ams*–anteromedial, *cls*–clypeal, *des*–dorsal epicranial, *dms*–dorsal malar, *fs*–frontal epicranial, *les*–lateral epicranial, *ligs*–ligular, *lrs*–labral, *mbs*–malar basiventral, *mds*–mandibular dorsal, *mpxs*–maxillary palp, *pes*–postepicranial, *pfs*–palpiferal, *pms*–postmental, *prms*–premental, *stps*–stipital, *ves*–ventral, *vms*–ventral malar.

**Figure 38.**
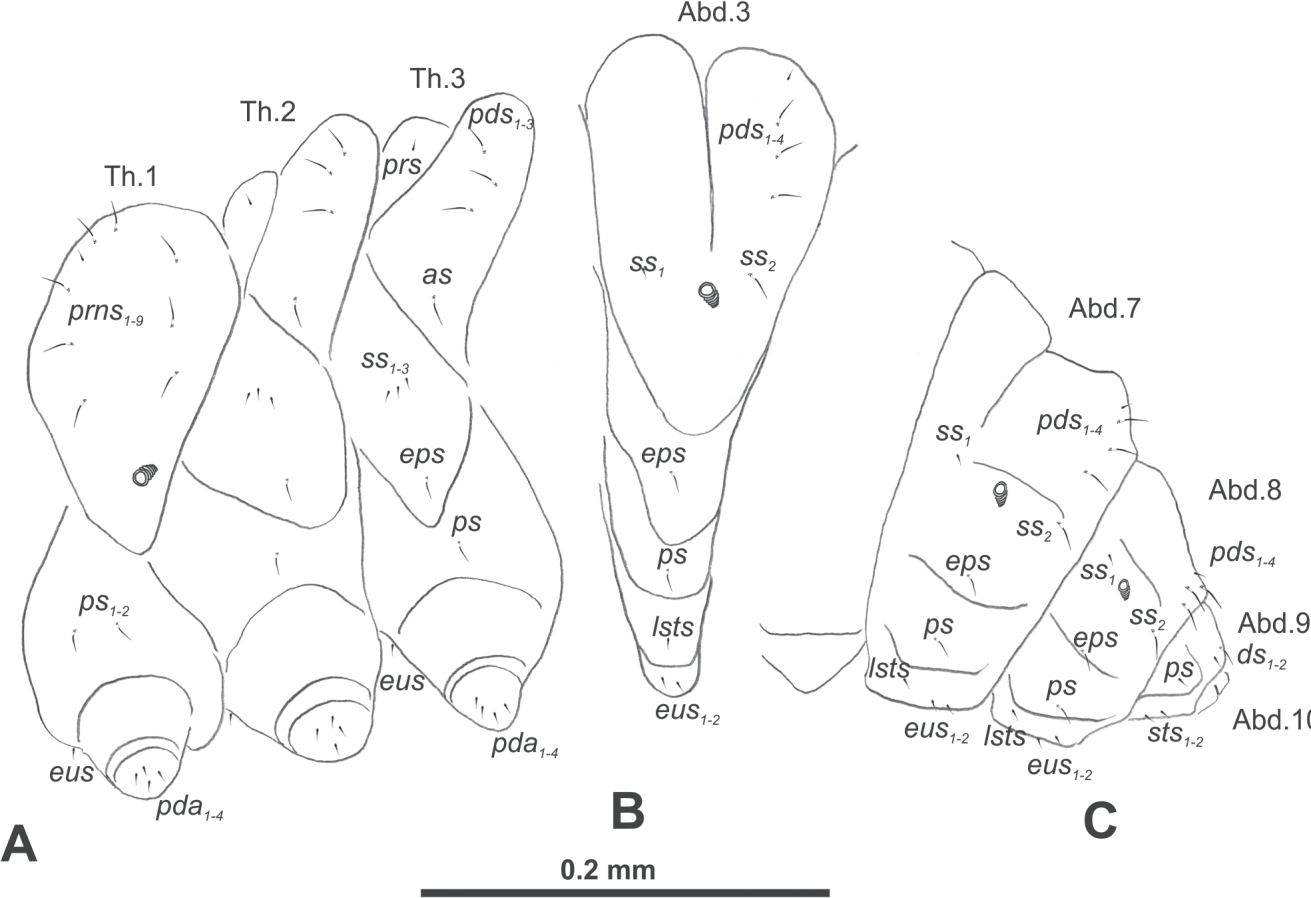
*Rhinusaherbarum* (H. Brisout de Barneville, 1862) mature larva, habitus **A** lateral view of thoracic segments **B** lateral view of abdominal segment I **C** lateral view of abdominal segments VII–X (schemes). Abbreviations: Th. 1–3–number of thoracic segments, Abd. 1–10–number of abdominal seg, setae: *as*–alar, *ds*–dorsal, *eps*–epipleural, *eus*–eusternal, *lsts*–laterosternal, *pda*–pedal, *pds*–postdorsal, *prns*–pronotal, *prs*–prodorsal, *ss*–spiracular, *ps*–pleural, *sts*–sternal.

**(Figs 36A, B, 37A–E, 38A–C). *Measurements*** (in mm). Body length: 2.50–4.00 (avg. 3.40). The widest place in the body (meso- and metathorax) measures up to 1.50. Head width: 0.50–0.55 (avg. 0.55).

***General*.** Body elongate, slender, strongly curved, rounded in cross section (Fig. 36A). All thoracic segments equal in size. Meso- and metathorax each divided dorsally into two folds (prodorsal fold vestigial, postdorsal fold prominent). Pedal folds of thoracic segments well isolated. Abdominal segments I–V of similar size, next segments tapering towards posterior body end. Abdominal segments I–VII each divided dorsally into two folds almost identical in size. Segments VIII and IX dorsally undivided. Epipleural folds of segments I–VIII conical well developed. Laterosternal and eusternal folds of segments I–VIII conical, well isolated. Abdominal segment X divided into four folds of equal size. Anus situated ventrally, almost completely hidden in the ninth segment.

Thoracic and abdominal spiracles unicameral; thoracic spiracles (Fig. 36A) placed laterally close to mesothorax; abdominal spiracles (Fig. 36A) placed medially on segments I–VIII.

***Colouration*.** Almost white to light yellow head (Fig. 36B). All thoracic and abdominal segments whitish (Fig. 36A). Cuticle covered with asperities.

***Vestiture*.** Setae on body thin, transparent, different in length (minute to medium).

***Head capsule*** (Figs 36B, 37A). Head suboval, endocarinal line present, reaching to 2/3 length of frons. Frontal sutures on head indistinct, very wide. Single pair of stemmata in the form of small black spots (st) placed laterally, close to the end of the frontal suture. *Des_1_* short, located in middle part of epicranium; long *des_2_* located anteriorly; long *des_3_* placed almost on the border of the frontal suture; minute *des_4_*, located laterally; and long *des_5_* placed anterolaterally above stemma (Fig. 37A). *Fs_1_* absent; *fs_2_* short, located posterolaterally; *fs_3_* absent; *fs_4_* long, located anteriorly; and long *fs_5_* located anterolaterally, close to antenna (Fig. 37A). *Les_1_* and *les_2_* medium; two short *ves*. Epicranial area with five *pes*.

***Antennae*** placed distally of the frontal suture, on the inside; membranous and distinctly convex basal article bearing one conical elongate sensorium, plus three sensilla basiconica (Fig. 37B).

***Clypeus*** (Fig. 37C) trapezoidal, ~ 2.5 × as wide as long with two short *cls*, localised posterolaterally, with one sensillum between them; anterior part distinctly less sclerotised than the basal part and slightly rounded towards the inside.

***Mouth parts*.** Labrum (Fig. 37C) ~ 2.2 × as wide as long, with three piliform *lrs*, various long; *lrs_1_* and *lrs_2_* elongated, located medially on small protuberances, and *lrs_3_* medium, located anterolaterally; anterior border bi-sinuate. Epipharynx (Fig. 37C) with two elongated finger-like *als*, identical in length and three piliform *ams* variable in length; labral rods (lr) distinct, rounded. Mandibles (Fig. 37D) bifid, cutting edge straight; two medium piliform and short *mds*, both located close to lateral border. Maxillolabial complex: maxilla brownish sclerotised (Fig. 37E), stipes with one *stps*, two *pfs* and one *mbs*, *stps* and both *pfs_1–2_* elongated; mala with five finger-like *dms* variable in length; four medium piliform *vms*. Maxillary palpi two-segmented; basal palpomere distinctly wider than distal one, with short *mpxs* and two sensilla, distal palpomere with a group of two apical sensilla in terminal receptive area. Prementum (Fig. 37E) close to oval-shaped, with two *prms* variable in length; ligula with slightly sinuate margin and two short *ligs*; premental sclerite broad, sclerotised, cup-shaped, posterior extension medium, with thick apex. Labial palpi one-segmented; palpi with a single pore, and single, apical sensilla in terminal receptive area; surface of labium smooth. Postmentum (Fig. 37E) with three *pms*, short *pms_1_* located posteromedially, long *pms_2_* located mediolaterally, and short *pms_3_* located anterolaterally; membranous area partially covered with knobby asperities.

***Thorax*.** Prothorax (Fig. 38A) with nine medium to short *prns*; two medium *ps*; and single short *eus*. Mesothorax (Fig. 38A) with a single minute *prs*; three medium *pds*; one medium *as*; three minute *ss*; one medium *eps*; one medium *ps*; and single minute *eus*. Chaetotaxy of metathorax (Fig. 38A) almost identical to that of mesothorax. Each pedal area of thoracic segments with four short to minute *pda*.

***Abdomen*.** Segments I–VIII (Fig. 38B, C) without *prs*; with four *pds* of various length; one minute and one medium *ss*; single, medium *eps*; one medium *ps*; one minute *lsts*; and two minute *eus*. Abdominal segment IX (Fig. 38C) with one medium and one minute *ds*; one minute *ps*; and two minute *sts*.

#### Description of pupa

**Figure 39.**
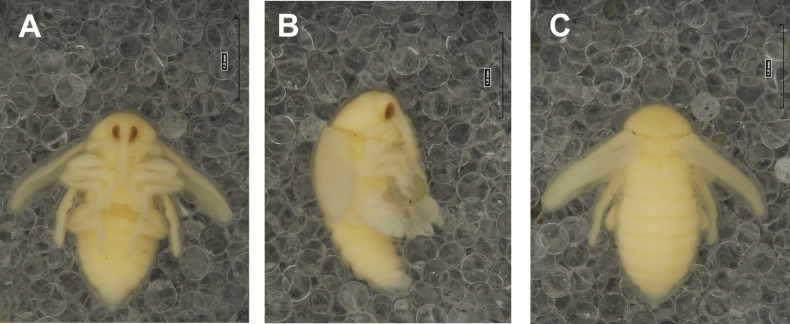
*Rhinusaherbarum* (H. Brisout de Barneville, 1862) pupa habitus **A** ventral view **B** lateral view **C** dorsal view.

**Figure 40.**
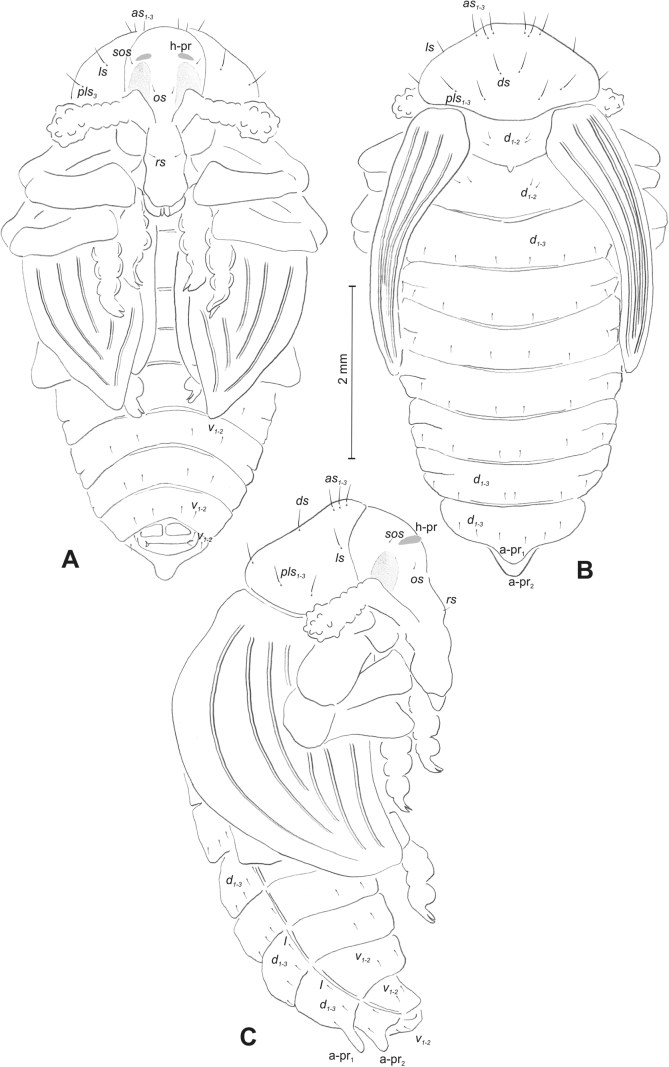
*Rhinusaherbarum* (H. Brisout de Barneville, 1862) pupa habitus **A** ventral view **B** dorsal view **C** lateral view (schemes). Abbreviations: a–pr–abdominal protuberances, h–pr–head protuberances, setae: *as*–apical, *d*–dorsal, *ds*–discal, *l*, *ls*–lateral, *os*–orbital, *pls*–posterolateral, *rs*–rostral, *sos*– supraorbital, *v*–ventral.

**(Figs 39A–C, 40A–C). *Measurements*** (in mm). Body length: 2.25–3.10; body width: 1.50–1.80; thorax width: 0.70–1.05; rostrum length: up to 0.70 ♂ and 1.20 ♀.

***Body*.** Integument white moderately elongated. Head and pronotum without protuberances. Rostrum rather short, reaching to mesocoxae; in both sexes 2.2 × as long as wide. Clubs covered with knobby protuberances. Pronotum trapezoidal, 2 × as wide as long. Meso- and metanotum similar in size. Abdominal segments I–IV almost identical in size; segments VI and VI tapering gradually, VII semicircular; segment VIII narrow; segment IX reduced. Abdominal segments VII and VIII each with semicircular, weakly sclerotised abdominal protuberances (a–pr_1, 2_). Urogomphi absent (Fig. 39A–C).

***Chaetotaxy*.** Well-developed setae, elongated to short, transparent. Head with a single short *sos*, single short *os*. Rostrum with a single short *rs* (Fig. 40A). Pronotum with three *as*, single *ls*, single *ds* and three *pls*; all pronotal setae elongated, equal in length. Dorsal parts of meso- and metathorax with two medium setae, placed medially (Fig. 40A–C). Abdominal segments I–VIII dorsally with three short setae dorsally, placed close to posterior margin of the segments. Each lateral part of abdominal segments I–VIII with a single short seta. Ventral parts of abdominal segments I–VIII with two short setae. Abdominal segment IX with two short setae ventrally (Fig. 40A–C).

#### Remarks and comparative notes.

This species is distributed in Central and Southern Europe and in North Africa (Alonso-Zarazaga et al. 2023). The shape of the rostrum in adults is similar in lateral view to that of some species of the *R.antirrhini* group but not in dorsal view, where the basal half in cross section is normally rectangular and not trapezoidal, and the scrobes are only slightly visible (Caldara and Toševski 2019).

#### Biological notes.

The host plants are *Kickxiaelatine* and *K.spuria* (L.) Dumort. The females oviposit in the seed capsules of the host plant during the summer, following the phenology of plant flowering. Larvae complete their development and pupate inside seed capsules. The new adults emerge in late summer (Caldara and Toševski 2019).

##### ﻿*Rhinusaneta* group

**Adult diagnosis.** Femora with a sharp tooth, which is more robust on metafemora; both body and apophysis of penis markedly long, taken together corresponding to length of whole abdomen; endophallus lacking inside body of penis and beginning from its base, with a long straight flagellum.

### ﻿9) Rhinusa collina

Taxon classificationAnimaliaColeopteraCurculionidae

(Gyllenhal, 1813)

E02A0F49-E3BF-5B39-81BF-76E37975D59F

#### Material examined.

8 mature larvae; 3 ♂ and 3 ♀ pupae. Serbia, Knjaževac, ex *Linariavulgaris* inside *R.linariae* galls, 05.07.2017, leg., det. I. Toševski.

#### Description of mature larva

**Figure 41.**
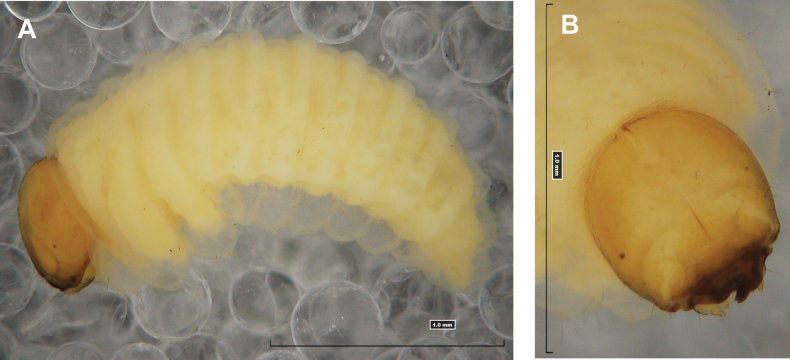
*Rhinusacollina* (Gyllenhal, 1813) mature larva **A** habitus **B** head, frontal view.

**Figure 42.**
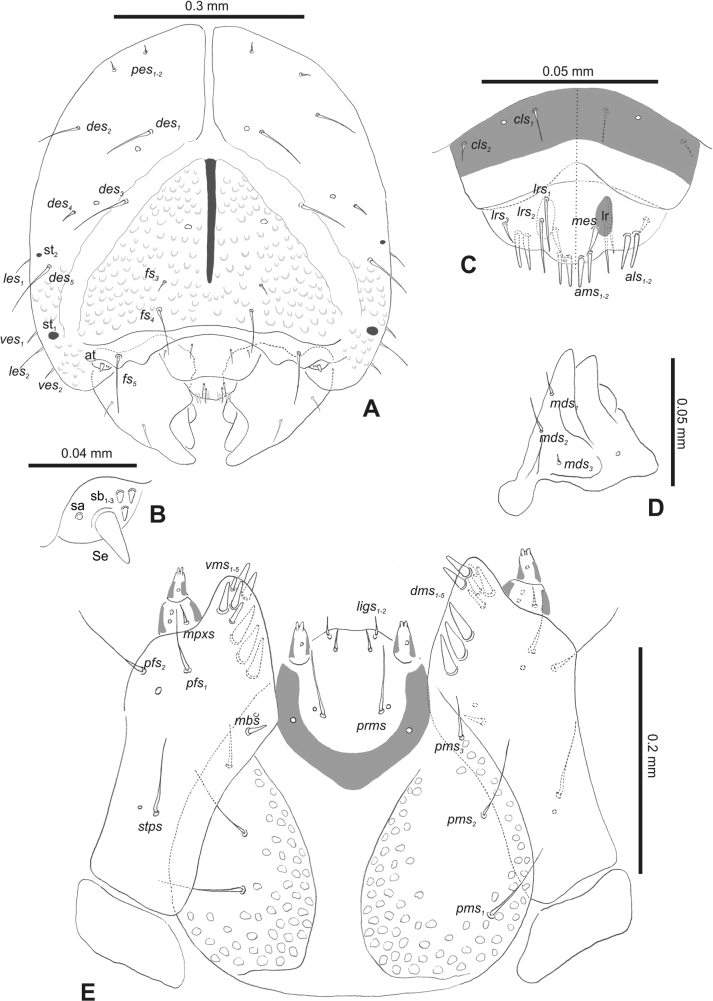
*Rhinusacollina* (Gyllenhal, 1813) mature larva, head and mouth parts **A** head **B** antenna **C** clypeus and labrum (left side), epipharynx (right side) **D** left mandible **E** maxillolabial complex (schemes). Abbreviations: at–antenna, lr–labral rods, sa–sensillum ampullaceum, sb–sensillum basiconicum, Se–sensorium, st–stemmata, setae: *als*–anterolateral, *ams*–anteromedial, *cls*–clypeal, *des*–dorsal epicranial, *dms*–dorsal malar, *fs*–frontal epicranial, *les*–lateral epicranial, *ligs*–ligular, *lrs*–labral, *mbs*–malar basiventral, *mds*–mandibular dorsal, *mes*–medial, *mpxs*–maxillary palp, *pes*–postepicranial, *pfs*–palpiferal, *pms*–postmental, *prms*–premental, *stps*–stipital, *ves*–ventral, *vms*–ventral malar.

**Figure 43.**
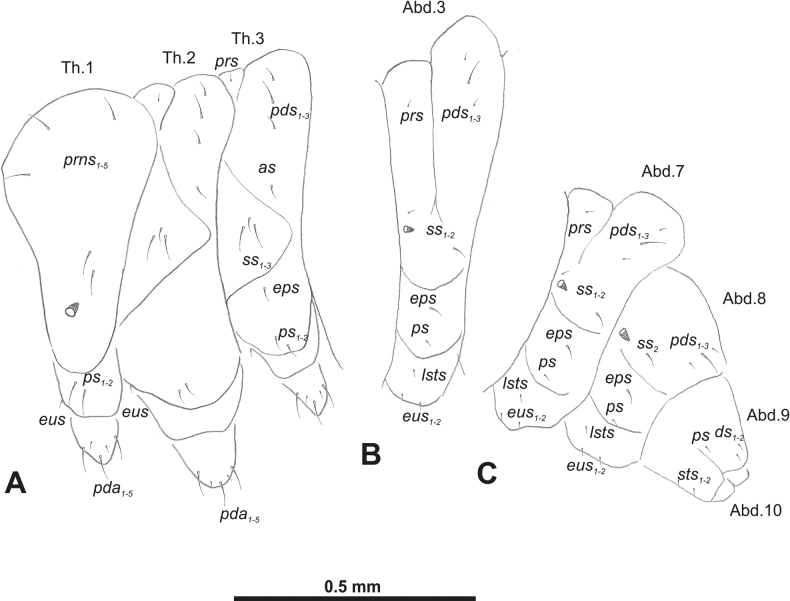
*Rhinusacollina* (Gyllenhal, 1813) mature larva, habitus **A** lateral view of thoracic segments **B** lateral view of abdominal segment I **C** lateral view of abdominal segments VII–X (schemes). Abbreviations: Th. 1–3–number of thoracic segments, Abd. 1–10–number of abdominal seg, setae: *as*–alar, *ds*–dorsal, *eps*–epipleural, *eus*–eusternal, *lsts*–laterosternal, *pda*–pedal, *pds*–postdorsal, *prns*–pronotal, *prs*–prodorsal, *ss*–spiracular, *ps*–pleural, *sts*–sternal.

**(Figs 41A, B, 42A–E, 43A–C). *Measurements*** (in mm). Body length: 2.60–3.00 (avg. 2.75). The widest place in the body (meso- and metathorax) measures up to 0.90. Head width: 0.55–0.65 (avg. 0.60).

***General*.** Body elongate, slender, distinctly curved, rounded in cross section (Fig. 41A). Prothorax prominent, pronotal shield not pigmented. Meso- and metathorax equal in size, smaller than prothorax; each divided dorsally into two folds (prodorsal fold distinctly smaller than postdorsal fold). Pedal folds of thoracic segments isolated, conical, prominent. Abdominal segments I–VI of similar size, next segments tapering towards posterior body end. Abdominal segments I–VII each divided dorsally into two various in size folds; postdorsal folds much higher than prodorsal folds. Segments VIII and IX dorsally undivided. Epipleural folds of segments I–VII conical. Laterosternal and eusternal folds of segments I–VIII conical, weakly isolated. Abdominal segment X divided into four folds of equal size. Anus situated ventrally, almost completely hidden in previous segment.

Thoracic and all abdominal spiracles unicameral; thoracic spiracles (Fig. 41A) placed ventrolaterally; abdominal spiracles (Fig. 41A) placed anteromedially on segments I–VIII.

***Colouration*.** Dark yellow to light brown head, medial parts of epicranium less sclerotised (Fig. 41B). Frons and ventrolateral parts of the head covered with knobby asperities. All thoracic and abdominal segments whitish. Cuticle covered with fine asperities (Fig. 41A).

***Vestiture*.** Setae on body thin, yellowish, different in length (minute to medium).

***Head capsule*** (Figs 41B, 42A). Head suboval, endocarinal line present, reaching to 3/4 length of frons. Frontal sutures on head distinct, very wide. Two pairs of stemmata various in size in the form of small black spots (st); first medium size located close to end of frontal suture, second small placed mediolaterally. *Des_1_* long, located in middle part of epicranium; long *des_2_*; long *des_3_* located anteriorly on epicranium close to the border with the frontal suture; *des_4_* very short; and *des_5_* elongated, located anterolaterally above stemma (Fig. 42A). *Fs_1_* and *fs_2_* absent; *fs_3_* minute, located medially; *fs_4_* long, located anteriorly; and long *fs_5_* located anterolaterally, close to antenna (Fig. 42A). *Les_1_* and *les_2_* medium; and two minute *ves*. Epicranial area with two *pes*.

***Antennae*** placed distally of the frontal suture, on the inside; membranous and distinctly convex basal article bearing one conical elongate sensorium, plus three sensilla basiconica and single ampullacea (Fig. 42B).

***Clypeus*** (Fig. 42C) trapezoidal, ~ 2.7 × as wide as long with two *cls*: *cls_1_* relatively long, *cls_2_* medium, both localised posterolaterally, with one sensillum between them; basal part distinctly more sclerotised than the apical part; anterior border slightly curved towards the inside.

***Mouth parts*.** Labrum (Fig. 42C) ~ 2 × as wide as long, with three piliform *lrs*, various length; *lrs_1_* elongated, located posteromedially, *lrs_2_* elongated, located medially, and *lrs_3_* short, located anterolaterally; anterior border bi-sinuate. Epipharynx (Fig. 42C) with two elongated finger-like *als*, almost identical in length; two piliform *ams* equally in length; and single finger-like *mes*; labral rods (lr) distinct, close to kidney shape. Mandibles (Fig. 42D) bifid, cutting edge with a single, blunt protuberance; two medium piliform and single minute *mds*, all located close to lateral border. Maxillolabial complex: maxilla dark sclerotised (Fig. 42E), stipes with one *stps*, two *pfs* and one *mbs* and one sensillum, *stps* and both *pfs_1–2_* relatively long; mala with five finger-like *dms* variable in length (divided into two groups); four piliform *vms*, medium to short in length. Maxillary palpi two-segmented; basal palpomere distinctly wider than distal one; length ratio of basal and distal palpomeres almost 1:1; basal palpomere with short *mpxs* and two sensilla, distal palpomere with a group of three or four apical sensilla in terminal receptive area. Prementum (Fig. 42E) oval-shaped, with one long *prms*; ligula with slightly sinuate margin and two short *ligs*; premental sclerite broad, sclerotised, U-shaped. Labial palpi one-segmented; palpi with a single pore, and a group of two or three apical sensilla (ampullacea) on terminal receptive area; surface of labium smooth. Postmentum (Fig. 42E) with three *pms*, elongated *pms_1_* located posteromedially, elongated *pms_2_* located laterally, and medium *pms_3_* located anterolaterally; lateral parts of membranous area covered with distinct knobby asperities.

***Thorax*.** Prothorax (Fig. 43A) with five medium *prns*: three placed apically, next two above stigma; two medium *ps*; and single short *eus*. Mesothorax (Fig. 43A) with one minute *prs*, two short and one medium *pds* (ordered: short, medium, short); one medium *as*; three *ss* (two medium and one minute); one medium *eps*; two medium *ps*; and single minute *eus*. Chaetotaxy of metathorax (Fig. 43A) almost identical to that of mesothorax. Each pedal area of thoracic segments with three medium and two minute *pda*.

***Abdomen*.** Segments I–VIII (Fig. 43B, C) with one minute *prs* (segment VIII without); two minute and one medium *pds*; one minute and one medium *ss*; one medium *eps*; one short *ps*; one minute *lsts*; and two minute *eus*. Abdominal segment IX (Fig. 43C) with two minute *ds*; one minute *ps*; and two minute *sts*.

#### Description of pupa

**Figure 44.**
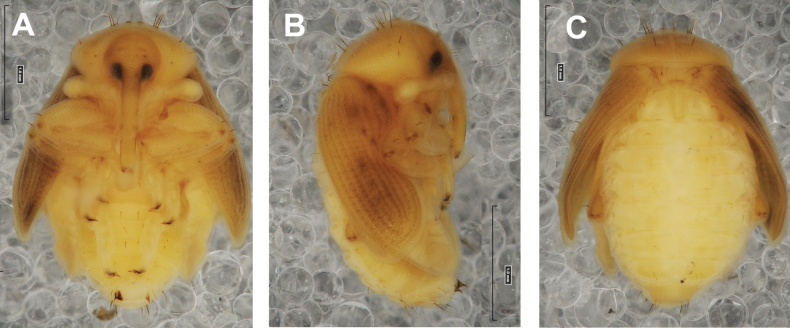
*Rhinusacollina* (Gyllenhal, 1813) pupa habitus **A** ventral view **B** lateral view **C** dorsal view.

**Figure 45.**
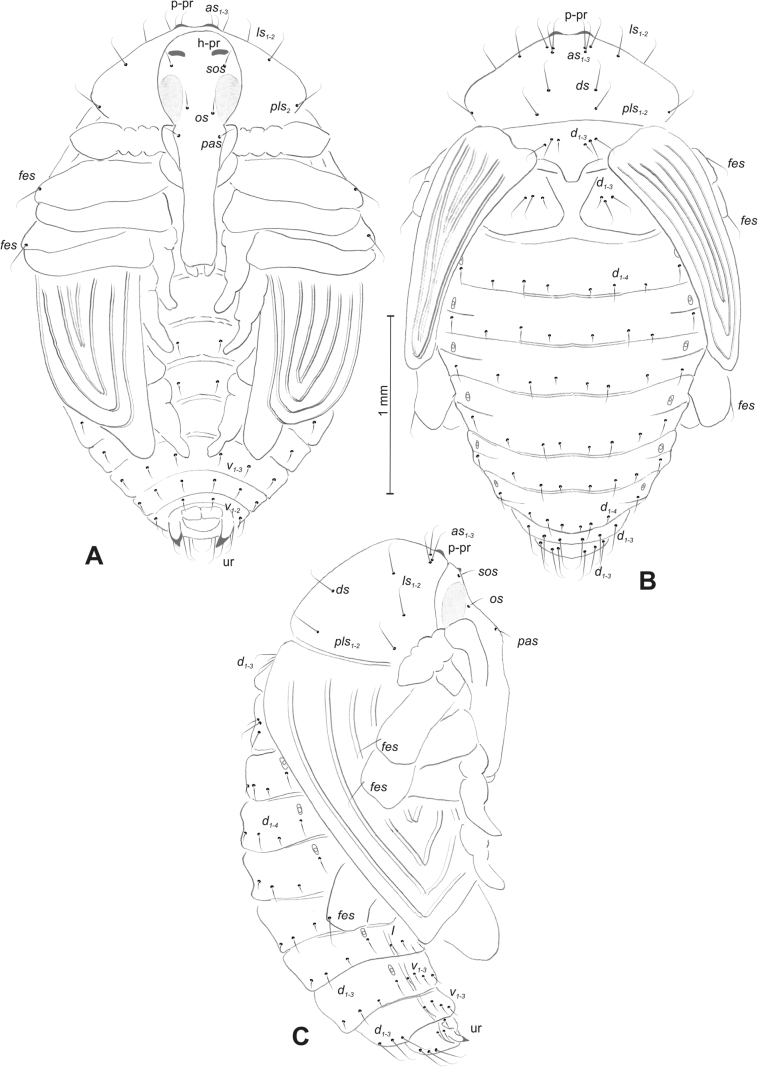
*Rhinusacollina* (Gyllenhal, 1813) pupa habitus **A** ventral view **B** dorsal view **C** lateral view (schemes). Abbreviations: a–pr–abdominal protuberances, p–pr–pronotal protuberances, ur–urogomphi, setae: *as*–apical, *d*–dorsal, *ds*–discal, *fes*–femoral, *l*, *ls*–lateral, *os*–orbital, *pas*–postantennal, *pls*–posterolateral, *sos*– supraorbital, *v*–ventral.

**(Figs 44A–C, 45A–C). *Measurements*** (in mm). Body length: 2.70–2.90 (avg. 2.75); body width: 1.75–1.80 (avg. 1.75); thorax width: 1.10–1.25 (avg. 1.15); rostrum length: up to 0.60 ♂ and 0.85 ♀.

***Body*.** Integument white, with some parts dark sclerotised; moderately stout, curved. Head with small head protuberances (h–pr) above eyes. Rostrum elongated, on both sexes almost 4 × as long as wide, distinctly protruding to mesocoxae. Pronotum trapezoidal 2.4 × as wide as long. Pronotal protuberances (p–pr) indistinct, flattened, sclerotised, separated at bases. Meso- and metanotum similar in size. Abdominal segments I–VI almost identical in size; segment VII semicircular; segment VIII narrow; segment IX reduced. Abdominal segment VIII dorsally with rounded, prominent, sclerotised abdominal protuberance (a–pr). Urogomphi (ur) medium, ending with sclerotised, sharp apexes (Fig. 44A–C).

***Chaetotaxy*.** Well developed, setae medium to elongated, transparent. Head with one medium *os* and one elongated *sos*. Rostrum with a single *pas* (Fig. 45A). Pronotum with three *as*, one *ds*, two *ls*, and two *pls* variable in length. Dorsal parts of meso- and metathorax with three setae of various length, placed medially. Apex of femora with a single long *fes* (Fig. 45A–C). Abdominal segments I–VI with four setae dorsally, variable in length: first and third minute, second and fourth medium, placed close to posterior margin of the segment. Abdominal segments VII and VIII with three elongated setae dorsally. Each lateral part of abdominal segments I–VII with a single short seta. Ventral parts of abdominal segments I–VIII with three medium setae. Abdominal segment IX with two short setae ventrally (Fig. 45A–C).

#### Remarks and comparative notes.

This species is widely distributed in Europe (Alonso-Zarazaga et al. 2023). In adults, the shape of the rostrum, together with the moderately elongated body, are useful characters that easily distinguish this species from the others in the group.

#### Biological notes.

*Rhinusacollina* is an inquiline weevil whose development is closely linked to root galls on *L.vulgaris* and *L.genistifolia* caused by *R.linariae*. The females oviposit eggs exclusively on well-developed galls that are not occupied by *R.linariae* larvae. For this reason, the competition of these inquiline weevils with gall inducers has never been observed (IT, pers. obs.).

### ﻿10) Rhinusa eversmanni

Taxon classificationAnimaliaColeopteraCurculionidae

(Rosenschoeld, 1838)

298AD9D9-9FDC-546E-A354-7EAA1DB4FB1F

#### Material examined.

9 mature larvae; 4 ♂ and 2 ♀ pupae. Serbia, Didići, ex *Linariavulgaris*, 05.07.2017, leg., det. I. Toševski.

#### Description of mature larva

**Figure 46.**
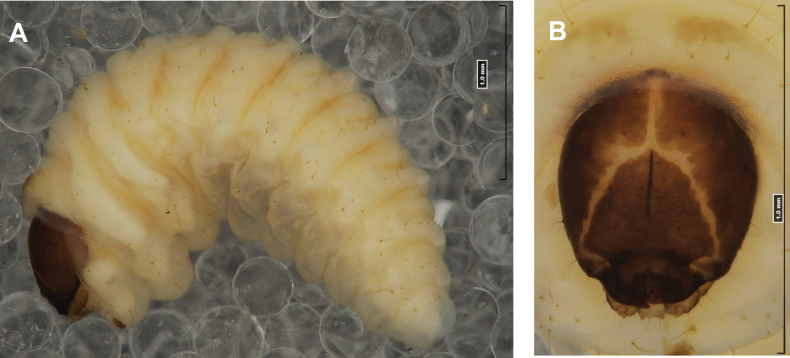
*Rhinusaeversmanni* (Rosenschoeld, 1838) mature larva **A** habitus **B** head, frontal view.

**Figure 47.**
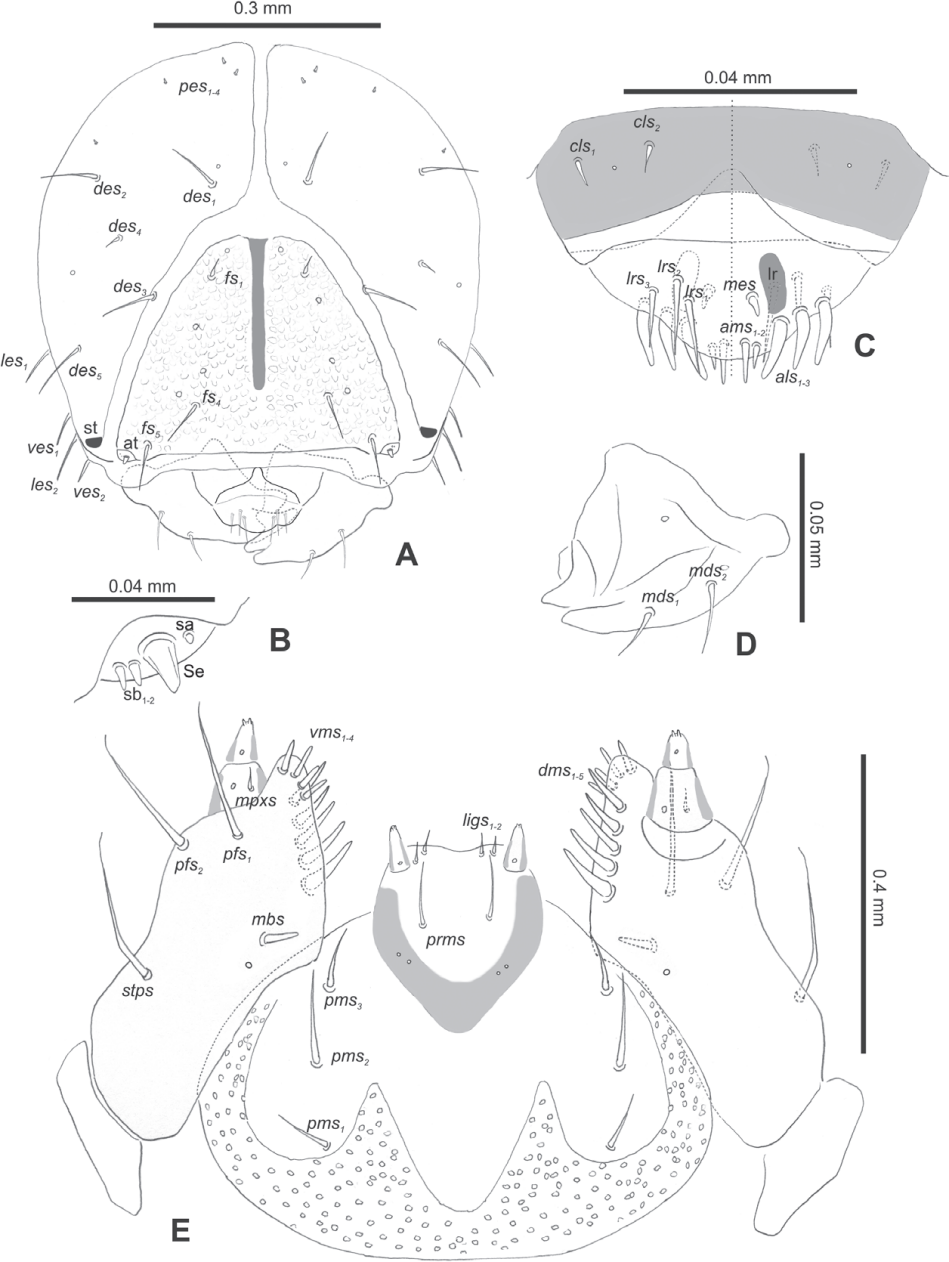
*Rhinusaeversmanni* (Rosenschoeld, 1838) mature larva, head and mouth parts **A** head **B** antenna **C** clypeus and labrum (left side), epipharynx (right side) **D** left mandible **E** maxillolabial complex (schemes). Abbreviations: at–antenna, lr–labral rods, sa–sensillum ampullaceum, sb–sensillum basiconicum, Se–sensorium, st–stemmata, setae: *als*–anterolateral, *ams*–anteromedial, *cls*–clypeal, *des*–dorsal epicranial, *dms*–dorsal malar, *fs*–frontal epicranial, *les*–lateral epicranial, *ligs*–ligular, *lrs*–labral, *mbs*–malar basiventral, *mds*–mandibular dorsal, *mes*–medial, *mpxs*–maxillary palp, *pes*–postepicranial, *pfs*–palpiferal, *pms*–postmental, *prms*–premental, *stps*–stipital, *ves*–ventral, *vms*–ventral malar.

**Figure 48.**
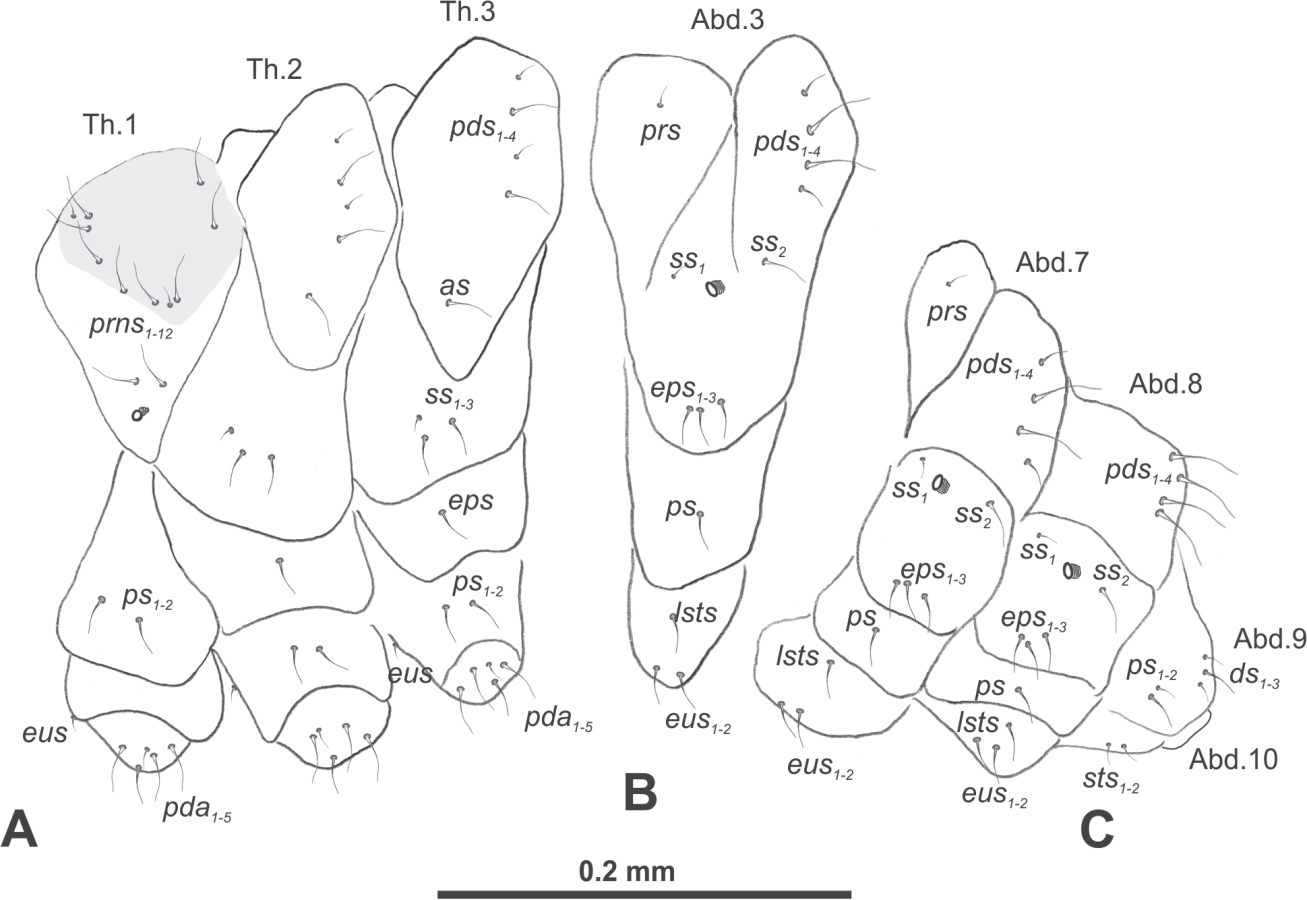
*Rhinusaeversmanni* (Rosenschoeld, 1838) mature larva, habitus **A** lateral view of thoracic segments **B** lateral view of abdominal segment I **C** lateral view of abdominal segments VII–X (schemes). Abbreviations: Th. 1–3–number of thoracic segments, Abd. 1–10–number of abdominal seg, setae: *as*–alar, *ds*–dorsal, *eps*–epipleural, *eus*–eusternal, *lsts*–laterosternal, *pda*–pedal, *pds*–postdorsal, *prns*–pronotal, *prs*–prodorsal, *ss*–spiracular, *ps*–pleural, *sts*–sternal.

**(Figs 46A, B, 47A–E, 48A–C). *Measurements*** (in mm). Body length: 2.70–4.10 (avg. 3.10). The widest place in the body (meso- and metathorax) measures up to 1.30. Head width: 0.60–065 (avg. 0.62).

***General*.** Body elongate, slender, strongly curved, rounded in cross section (Fig. 46A). All thoracic segments equal in size. Meso- and metathorax each divided dorsally into two folds (prodorsal fold vestigial, postdorsal fold prominent). Pedal folds of thoracic segments isolated, conical. Abdominal segments I–III of similar size, next segments tapering towards posterior body end. Abdominal segments I–VII each divided dorsally into two folds: postdorsal folds much higher than prodorsal folds. Segments VIII and IX dorsally undivided. Epipleural folds of segments I–VIII conical. Laterosternal and eusternal folds of segments I–VIII conical, well isolated. Abdominal segment X divided into four folds of equal size. Anus situated ventrally.

Thoracic and abdominal spiracles unicameral; thoracic spiracles (Fig. 46A) placed laterally close to mesothorax; abdominal spiracles (Fig. 46A) placed medially on segments I–VIII.

***Colouration*.** Dark brown to brown head, medial parts of epicranium less sclerotised (Fig. 46B). Prodorsal sclerite brownish. All thoracic and abdominal segments whitish (Fig. 46A). Cuticle densely covered with cuticular asperities. All setae of thorax and abdomen placed on dark brown spots.

***Vestiture*.** Setae on body thin, brown, different in length (minute to long).

***Head capsule*** (Figs 46B, 47A). Head suboval, endocarinal line present, reaching to the 3/4 of the length of frons. Frontal sutures on head distinct, very wide. Frons covered with knobby, dark asperities. Single pair of stemmata in the form of small black spots (st) placed laterally, close to the end of the frontal suture. *Des_1_* long, located in middle part of epicranium; long *des_2_* located anteriorly; long *des_3_* placed almost on the border of the frontal suture; minute *des_4_*, located laterally; and elongated *des_5_* placed anterolaterally above stemma (Fig. 47A). *Fs_1_* short, located posterolaterally; *fs_2_* and *fs_3_* absent; *fs_4_* long, located anteriorly; and long *fs_5_* located anterolaterally, close to antenna (Fig. 47A). *Les_1_* and *les_2_* medium; two short *ves*. Epicranial area with four *pes*.

***Antennae*** placed distally of the frontal suture, on the inside; membranous and distinctly convex basal article bearing one conical, moderately elongate sensorium, plus a single sensillum ampullaceum and two sensilla basiconica (Fig. 47B).

***Clypeus*** (Fig. 47C) trapezoidal, ~ 2.7 × as wide as long with two short *cls*, localised posterolaterally, with one sensillum between them; posterior part distinctly less sclerotised than the basal part; anterior border straight.

***Mouth parts*.** Labrum (Fig. 47C) ~ 2.4 × as wide as long, with three piliform *lrs*, various long; *lrs_1_* and *lrs_2_* elongated, located medially, *lrs_3_* medium, located anterolaterally; anterior border bi-sinuate. Epipharynx (Fig. 47C) with three elongated finger-like *als*, almost identical in length; two piliform *ams* variable in length; single finger-like *mes*; labral rods (lr) distinct, kidney shaped. Mandibles (Fig. 47D) bifid, cutting edge with additional, blunt tooth; two medium piliform and short *mds*, both located close to lateral border. Maxillolabial complex: maxilla brownish sclerotised (Fig. 47E), stipes with one *stps*, two *pfs* and one short *mbs* and one sensillum, *stps* and both *pfs_1–2_* elongated; mala with five finger-like *dms* variable in length; four piliform *vms*, medium to short in length. Maxillary palpi two-segmented; basal palpomere wider and longer than distal one; basal palpomere with short *mpxs* and single sensillum, distal palpomere with a group of two or three apical sensilla in terminal receptive area. Prementum (Fig. 47E) close to oval-shaped, with one long *prms*; ligula with slightly sinuate margin and two short *ligs*; premental sclerite well sclerotised, U-shaped. Labial palpi one-segmented; palpi with a single pore, and a pair of apical sensilla (ampullacea) on terminal receptive area; surface of labium smooth. Postmentum (Fig. 47E) with three *pms*, medium *pms_1_* located posteromedially, long *pms_2_* located mediolaterally, and medium *pms_3_* located anterolaterally; membranous area partially covered with knobby asperities.

***Thorax*.** Prothorax (Fig. 48A) with 12 elongated to short *prns*, ten of them placed on dorsal sclerite; two medium *ps*; and single short *eus*. Mesothorax (Fig. 48A) without *prs*; with two elongated and two short *pds* (ordered: short, long, short, long); one long *as*; two long and single minute *ss*; one long *eps*; two long *ps*; and single minute *eus*. Chaetotaxy of metathorax (Fig. 48A) almost identical to that of mesothorax. Each pedal area of thoracic segments with four relatively long and one minute *pda*.

***Abdomen*.** Segments I–VIII (Fig. 48B, C) with one minute *prs* (segments VII and VIII without); four *pds* of various length; one minute and one long *ss*; three long *eps*; one long *ps*; one medium *lsts*; and two short *eus*. Abdominal segment IX (Fig. 48C) with two minute and one medium *ds*; one medium and one minute *ps*; and two minute *sts*.

#### Description of pupa

**Figure 49.**
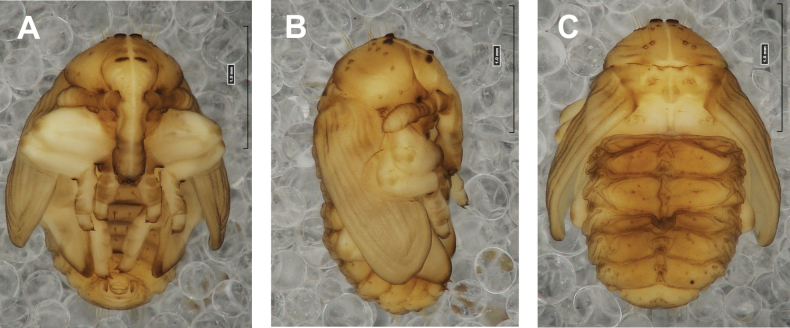
*Rhinusaeversmanni* (Rosenschoeld, 1838) pupa habitus **A** ventral view **B** lateral view **C** dorsal view.

**Figure 50.**
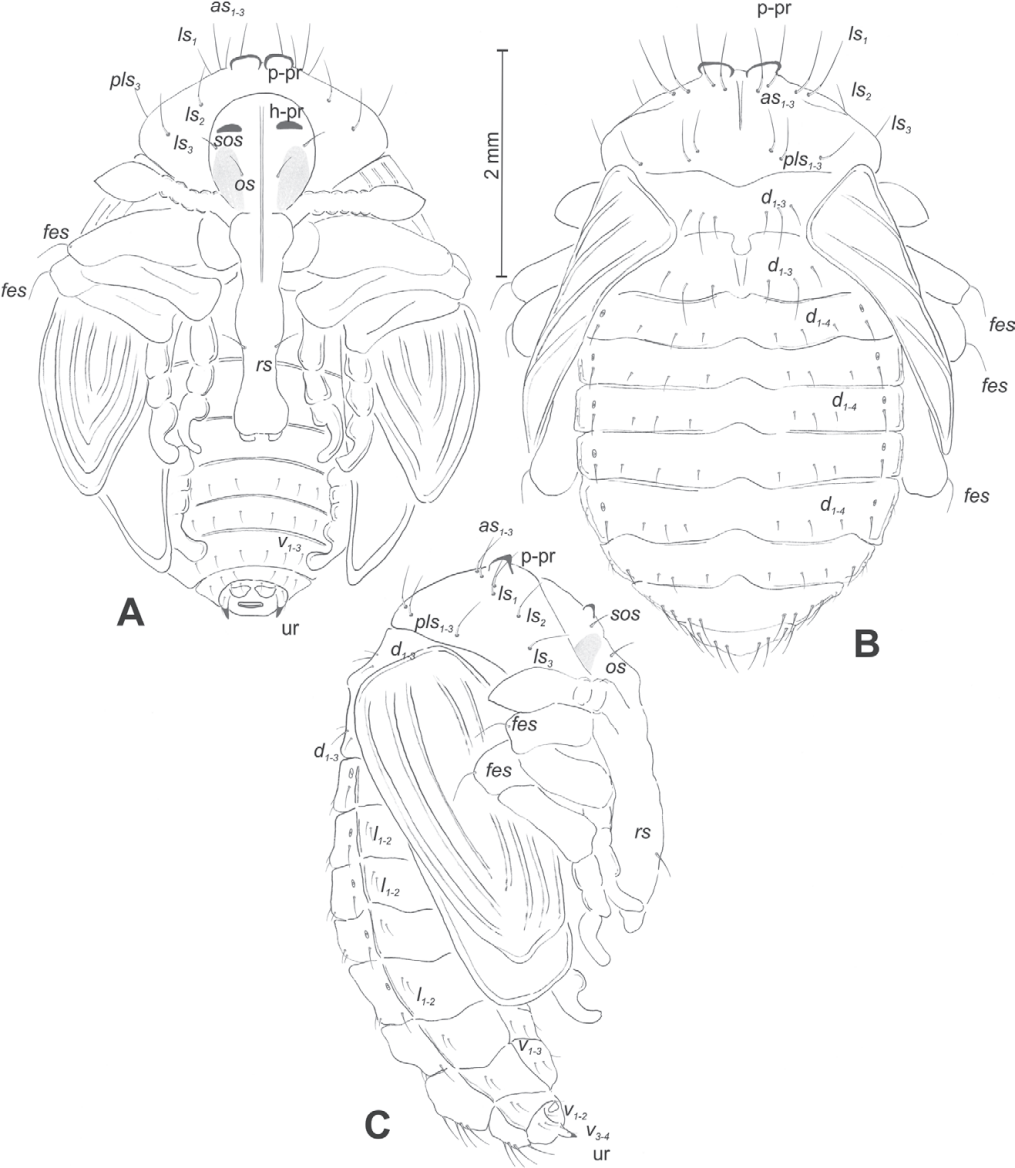
*Rhinusaeversmanni* (Rosenschoeld, 1838) pupa habitus **A** ventral view **B** lateral view **C** dorsal view (schemes). Abbreviations: a–pr–abdominal protuberances, p–pr–pronotal protuberances, ur–urogomphi, setae: *as*–apical, *d*–dorsal, *ds*–discal, *fes*–femoral, *l*, *ls*–lateral, *os*–orbital, *pas*–postantennal, *pls*–posterolateral, *rs*–rostral, *sos*– supraorbital, *v*–ventral.

**(Figs 49A–C, 50A–C). *Measurements*** (in mm). Body length: 2.36–2.76; body width: 1.73–2.00; thorax width: 1.03–1.20; rostrum length: up to 0.86 ♂ and 1.23 ♀.

***Body*.** Integument white, stout. Head elongated protuberances present on head above eyes (h–pr). Rostrum elongated, reaching over mesocoxae (almost 4.2 × as wide as long on both male and female). Pronotum trapezoidal 2.5 × as wide as long. Pronotal setae placed on dark brown spots. Pronotal protuberances (p–pr) conical, flattened, sclerotised, separated at bases. Meso- and metanotum similar in size. Abdominal segments I–IV almost identical in size; segments V and VI tapering gradually, VII semicircular; segment VIII narrow; segment IX reduced. Urogomphi (ur) short, ending with sclerotised, sharp apexes (Fig. 49A–C).

***Chaetotaxy*.** Well developed, setae elongated to short, transparent. Head with one medium *sos* and one medium *os*. Rostrum with a single *rs* (Fig. 50A). Pronotum with three *as*, three *ls*, and three *pls* all elongated, equal in length. Dorsal parts of meso- and metathorax with three setae of various length, placed medially. Apex of femora with a single long *fes* (Fig. 50A–C). Abdominal segments I–VII dorsally with four setae dorsally, variable in length: first and third minute, second, short, and fourth medium; setae first to third placed close to posterior margin of the segment, fourth placed below stigma (on segment VII all setae medium). Abdominal segments VII and VIII with four elongated setae dorsally. Each lateral part of abdominal segments I–VIII with one short seta. Ventral parts of abdominal segments I–VIII with three short setae. Abdominal segment IX with four short setae ventrally (Fig. 50A–C).

#### Remarks and comparative notes.

This is an uncommon species with a wide and fragmentary distribution: France, Italy, Germany, Czech Republic, Ukraine, Russia, Kazakhstan, Uzbekistan, and Tajikistan (Alonso-Zarazaga et al. 2023). At the adult stage, it is easily distinguishable from the other species of the group by the shape and length of the rostrum, especially in the female, which has a longer antennal club than all the other species of the genus.

#### Biological notes.

The stem galls caused by *R.pilosa* on *L.vulgaris* and by *R.rara* on *L.genistifolia* or *L.dalmatica* are niches for the development of *R.eversmanni*, which is another inquiline weevil. The females oviposit their eggs on fully developed galls, and hatched larvae bore holes towards the central portion of the gall, where larvae of the gall inducer are positioned. Larvae of *R.eversmanni* are very aggressive, killing all resident larvae inside galls, while competition between them inside galls is pronounced and cannibalism is commonly observed. Over 20 adults of *R.eversmanni* can develop in the larger gall induced by *R.pilosa* or *R.rara* (Toševski et al. 2015).

### ﻿11) Rhinusa incana

Taxon classificationAnimaliaColeopteraCurculionidae

(Kirsch, 1881)

7FE726F9-3AE0-57FC-8E3F-1B3D0F9F8014

#### Material examined.

5 mature larvae. Italia, Sicilia, San Cono, ex *Linariamulticaulis* (L.) Mill., 06.05.2017, leg. C. Baviera, det. R. Caldara.

#### Description of mature larva

**Figure 51.**
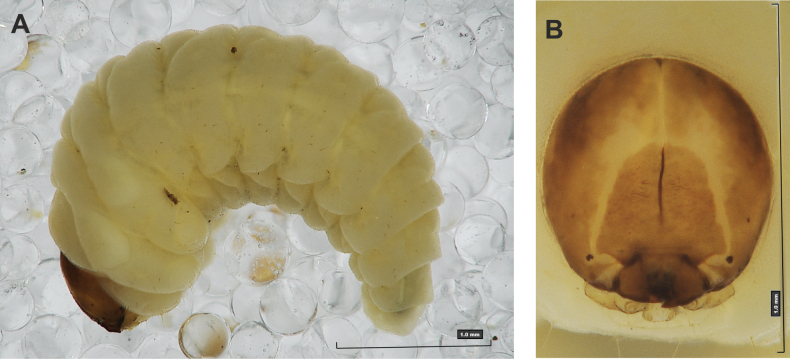
*Rhinusaincana* (Kirsch, 1881) mature larva **A** habitus **B** head, frontal view.

**Figure 52.**
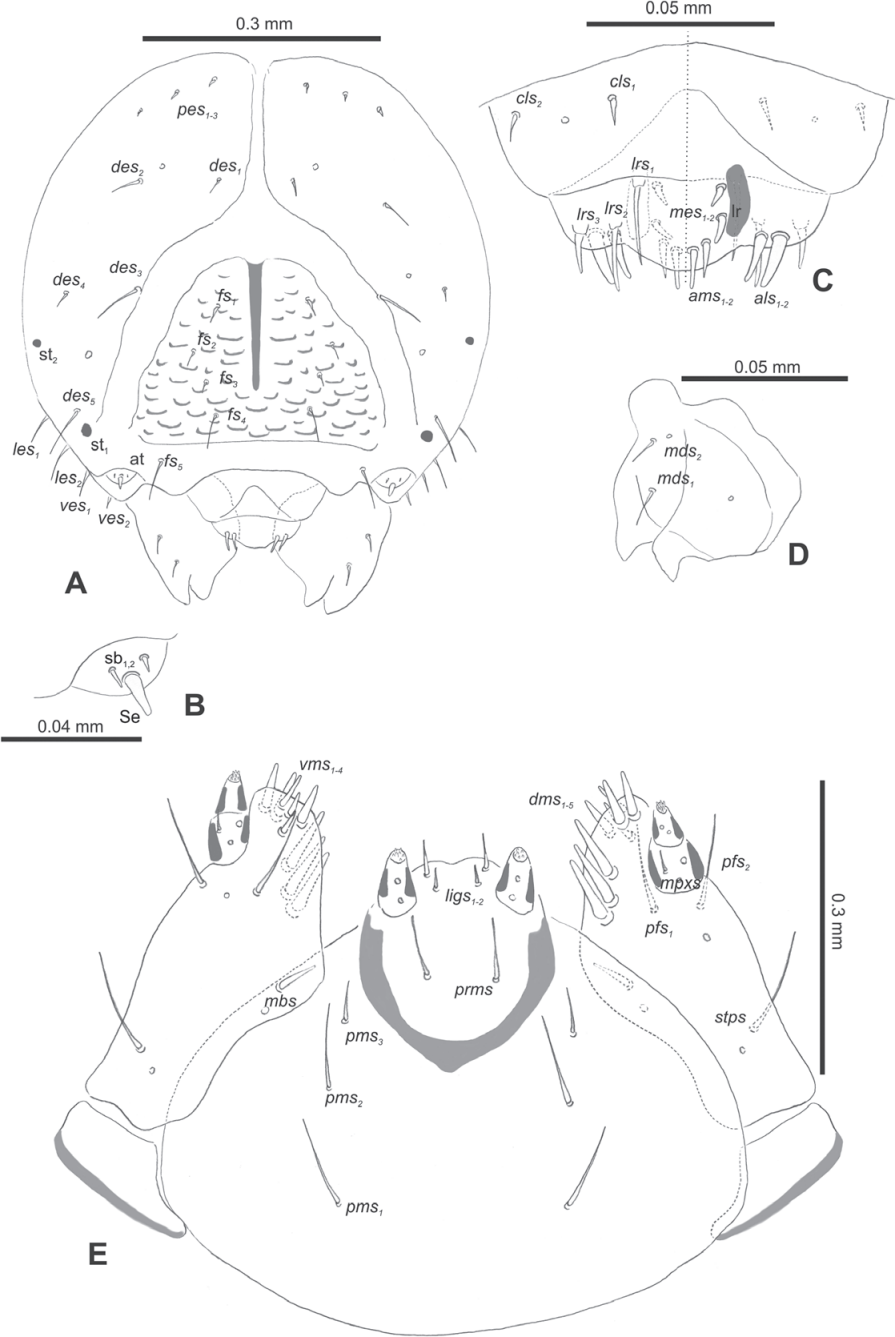
*Rhinusaincana* (Kirsch, 1881) mature larva, head and mouth parts **A** head **B** antenna **C** clypeus and labrum (left side), epipharynx (right side) **D** left mandible **E** maxillolabial complex (schemes). Abbreviations: at–antenna, lr–labral rods, sb–sensillum basiconicum, Se–sensorium, st–stemmata, setae: *als*–anterolateral, *ams*–anteromedial, *cls*–clypeal, *des*–dorsal epicranial, *dms*–dorsal malar, *fs*–frontal epicranial, *les*–lateral epicranial, *ligs*–ligular, *lrs*–labral, *mbs*–malar basiventral, *mds*–mandibular dorsal, *mes*–medial, *mpxs*–maxillary palp, *pes*–postepicranial, *pfs*–palpiferal, *pms*–postmental, *prms*–premental, *stps*–stipital, *ves*–ventral, *vms*–ventral malar.

**Figure 53.**
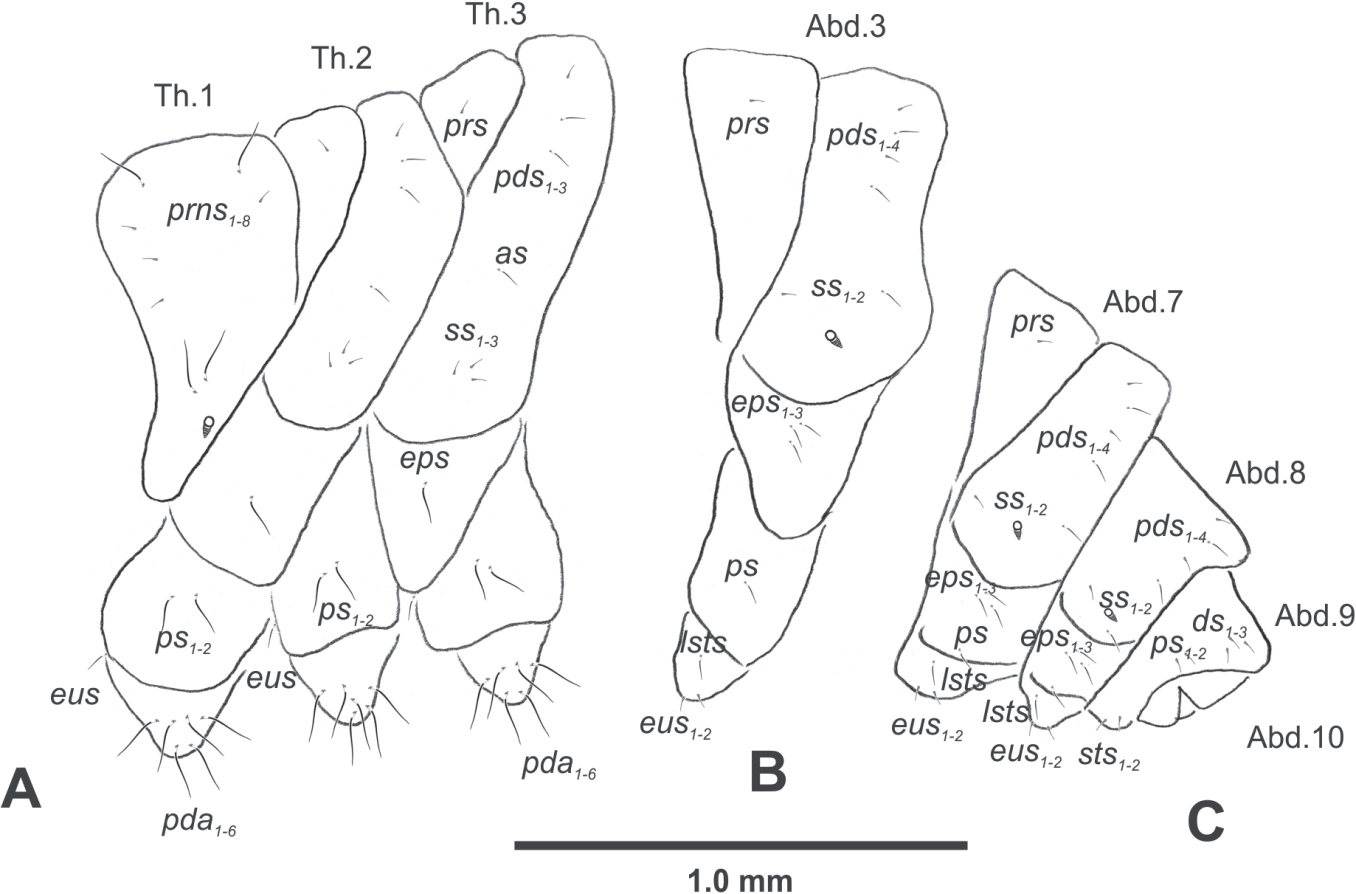
*Rhinusaincana* (Kirsch, 1881) mature larva, habitus **A** lateral view of thoracic segments **B** lateral view of abdominal segment I **C** lateral view of abdominal segments VII–X (schemes). Abbreviations: Th. 1–3–number of thoracic segments, Abd. 1–10–number of abdominal seg, setae: *as*–alar, *ds*–dorsal, *eps*–epipleural, *eus*–eusternal, *lsts*–laterosternal, *pda*–pedal, *pds*–postdorsal, *prns*–pronotal, *prs*–prodorsal, *ss*–spiracular, *ps*–pleural, *sts*–sternal.

**(Figs 51A, B, 52A–E, 53A–C). *Measurements*** (in mm). Body length: 2.25–3.00 (avg. 2.50). The widest place in the body (meso- and metathorax) measures up to 1.50. Head width: 0.60–070 (avg. 0.65).

***General*.** Body elongate, slender, slightly curved, rounded in cross section (Fig. 51A). All thoracic segments equal in size. Meso- and metathorax each divided dorsally into two folds (prodorsal fold smaller than postdorsal fold). Pedal folds of thoracic segments isolated, conical. Abdominal segments I–V of similar size, next segments tapering towards posterior body end. Abdominal segments I–VII each divided dorsally into two folds: postdorsal folds much higher than prodorsal folds. Segments VIII and IX dorsally undivided. Epipleural folds of segments I–VIII conical. Laterosternal and eusternal folds of segments I–VIII conical, well isolated. Abdominal segment X divided into four folds of equal size. Anus situated ventrally, hidden in ninth segment.

Thoracic and abdominal spiracles unicameral; thoracic spiracles (Fig. 51A) placed laterally close to mesothorax; abdominal spiracles (Fig. 51A) placed medially on segments I–VIII.

***Colouration*.** Yellow to brown head, medial parts of epicranium less sclerotised (Fig. 51B). All thoracic and abdominal segments whitish, covered with fine asperities (Fig. 51A).

***Vestiture*.** Setae on body thin, transparent, different in length (minute to long).

***Head capsule*** (Figs 51B, 52A). Head slightly narrowed bilaterally, endocarinal line present, reaching to 3/4 length of frons. Frontal sutures on head partially indistinct, very wide. Frons covered with knobby, dark asperities. Two pairs of stemmata in the form of small black spots (st) placed laterally. *Des_1_* very short, located in middle part of epicranium; medium *des_2_* located anteriorly; long *des_3_* placed almost on the border of the frontal suture; minute *des_4_*, located laterally; and elongated *des_5_* placed close to stemma (Fig. 52A). *Fs_1_* minute, located posterolaterally; *fs_2_* and *fs_3_* minute; *fs_4_* medium, located anteriorly; and long *fs_5_* located anterolaterally, close to antenna (Fig. 52A). *Les_1_* and *les_2_* medium; two short *ves*. Epicranial area with three *pes*.

***Antennae*** placed distally of the frontal suture, on the inside; membranous and distinctly convex basal article bearing one conical elongate sensorium, plus two sensilla basiconica (Fig. 52B).

***Clypeus*** (Fig. 52C) trapezoidal, ~ 2.7 × as wide as long with two medium *cls*, localised posterolaterally, with one sensillum between them; anterior border straight.

***Mouth parts*.** Labrum (Fig. 52C) ~ 2.4 × as wide as long, with three piliform *lrs*, various long; *lrs_1_* elongated, placed posteromedially, *lrs_2_* elongated, located anteromedially, *lrs_3_* medium, located anterolaterally; anterior border bi-sinuate. Epipharynx (Fig. 52C) with two elongated finger-like *als*, almost identical in length, two piliform *ams* variable in length and two finger-like *mes* variable in length; labral rods (lr) distinct, elongated. Mandibles (Fig. 52D) bifid, with two medium piliform *mds*, both located close to lateral border. Maxillolabial complex: maxilla brownish sclerotised (Fig. 52E) stipes with one *stps*, two *pfs* and one relatively long *mbs* and one sensillum, *stps* and both *pfs_1–2_* elongated; mala with five finger-like *dms* variable in length, placed in two groups (three + two); four piliform, *vms*, medium to short in length. Maxillary palpi two-segmented; basal palpomere slightly wider than distal one; basal palpomere with short *mpxs* and single sensillum, terminal receptive area of distal palpomere with a group of five or six apical sensilla; basal and distal palpomeres almost of the same length. Prementum (Fig. 52E) close to cup-shaped, with one long *prms*; ligula with distinctly sinuate margin and two *ligs* variable in length; premental sclerite well sclerotised, U-shaped. Labial palpi one-segmented; palpi with single pore; terminal receptive area with four or five apical sensilla (ampullacea); surface of labium smooth. Postmentum (Fig. 52E) with three *pms*, elongated *pms_1_* located posteromedially, long *pms_2_* located mediolaterally, and medium *pms_3_* located anterolaterally; membranous area smooth.

***Thorax*.** Prothorax (Fig. 53A) with eight *prns* (four relatively long and four short); two elongated *ps*; and single short *eus*. Mesothorax (Fig. 53A) with minute *prs*, single short and two medium *pds*; one minute *as*; three minute *ss*; two long *eps*; single long *ps*; and single minute *eus*. Chaetotaxy of metathorax (Fig. 53A) almost identical to that of mesothorax. Each pedal area of thoracic segments with six relatively long *pda*.

***Abdomen*.** Segments I–VIII (Fig. 53B, C) with one minute *prs* (segment VIII without); four medium *pds*; one minute and one medium *ss*; three medium *eps*; one medium *ps*; one medium *lsts*; and two minute *eus*. Abdominal segment IX (Fig. 53C) with two short and one minute *ds*; one medium and one minute *ps*; and two minute *sts*.

#### Remarks and comparative notes.

This species is distributed in the Iberian Peninsula, in southern Italy and Sicily, and in the western part of North Africa (Alonso-Zarazaga et al. 2023). It is very closely related to *R.neta*, from which it can be separated at adult stage by a few but constant characters (shape of the female rostrum, antennae inserted slightly more towards the base of the rostrum in both sexes, and scales of the dorsal vestiture slightly thinner).

#### Biological notes.

This species was collected in Sicily inside the seed capsules of Linariamulticaulissubsp.aetnensis Giardina and Zizza, L.multicaulissubsp.humilis (Guss.) De Leon. (Baviera and Caldara 2020) and *L.striata* (Lam.) DC. (Goggi 2004). In Algeria, its development occurs inside the seed capsules of *L.baborensis* Batt. and *L.heterophylla* Desf. (de Peyerimhoff 1911).

### ﻿12) Rhinusa neta

Taxon classificationAnimaliaColeopteraCurculionidae

(Germar, 1821)

AC465478-41D1-5C0E-9C8F-AA6D6977706D

#### Material examined.

21 mature larvae; 7 ♂ and 12 ♀ pupae. Serbia, Zemun, ex *Linariavulgaris*, 15.08.2017, leg., det. I. Toševski.

#### Redescription of mature larva

**Figure 54.**
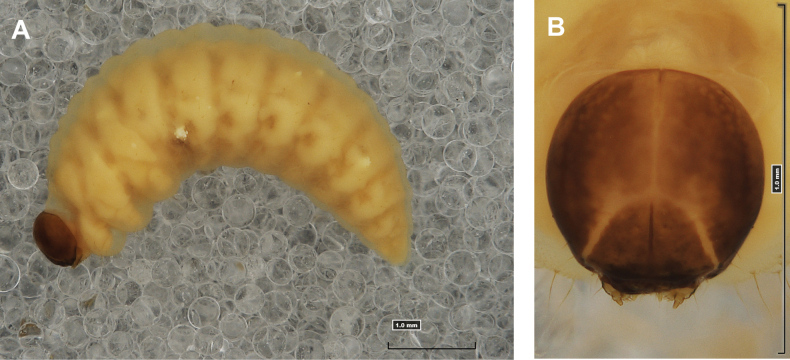
*Rhinusaneta* (Germar, 1821) mature larva **A** habitus **B** head, frontal view.

**Figure 55.**
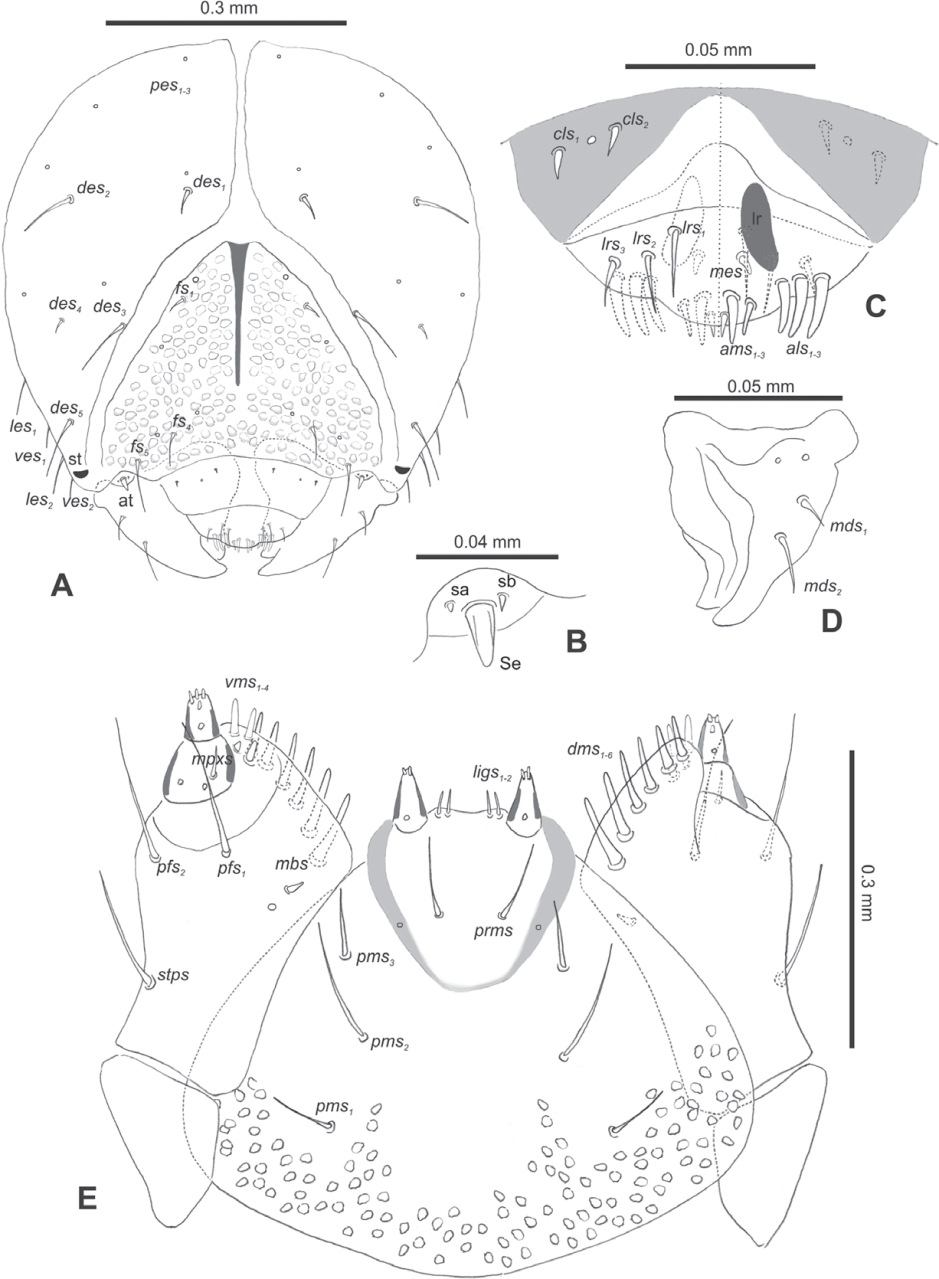
*Rhinusaneta* (Germar, 1821) mature larva, head and mouth parts **A** head **B** antenna **C** clypeus and labrum (left side), epipharynx (right side) **D** left mandible **E** maxillolabial complex (schemes). Abbreviations: at–antenna, lr–labral rods, sa–sensillum ampullaceum, sb–sensillum basiconicum, Se–sensorium, st–stemmata, setae: *als*–anterolateral, *ams*–anteromedial, *cls*–clypeal, *des*–dorsal epicranial, *dms*–dorsal malar, *fs*–frontal epicranial, *les*–lateral epicranial, *ligs*–ligular, *lrs*–labral, *mes*–medial, *mbs*–malar basiventral, *mds*–mandibular dorsal, *mpxs*–maxillary palp, *pes*–postepicranial, *pfs*–palpiferal, *pms*–postmental, *prms*–premental, *stps*–stipital, *ves*–ventral, *vms*–ventral malar.

**Figure 56.**
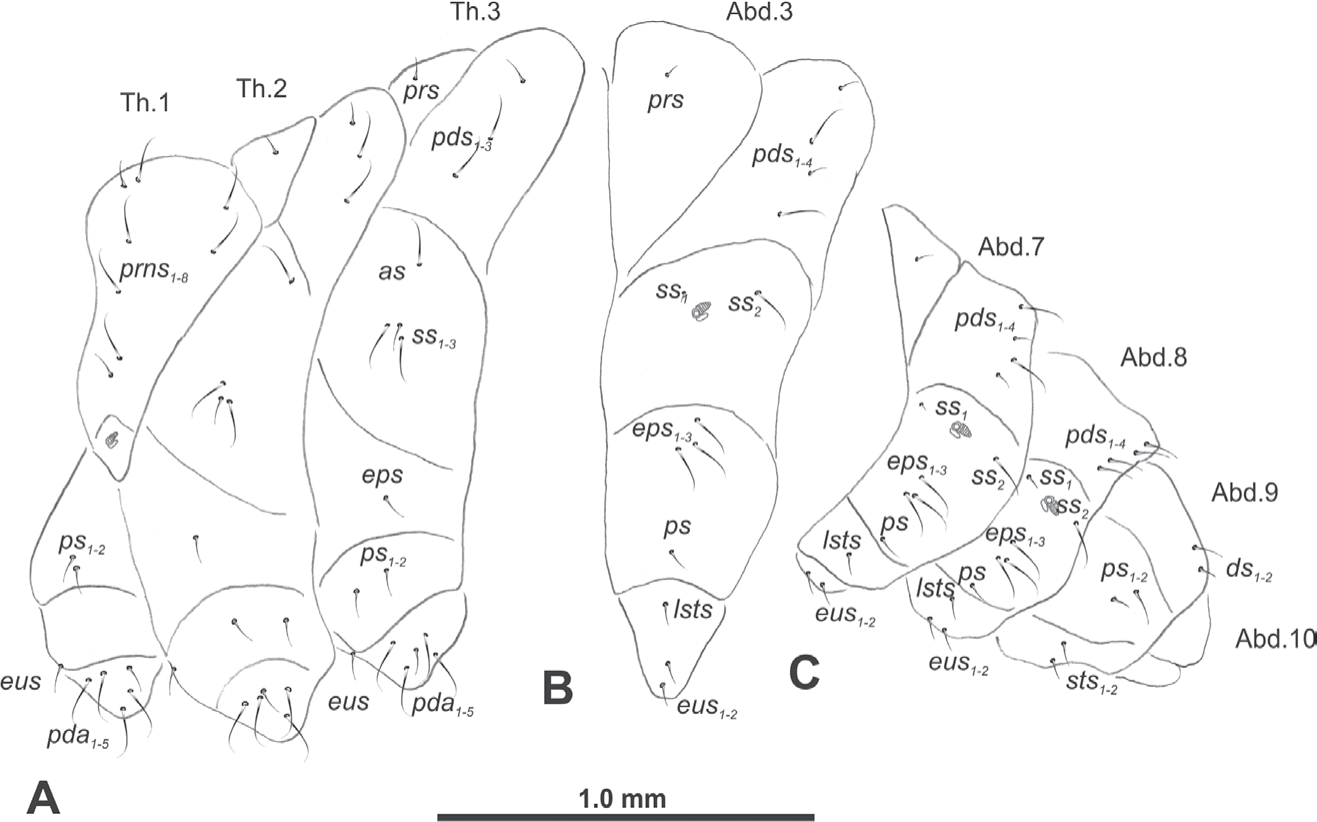
*Rhinusaneta* (Germar, 1821) mature larva, habitus **A** lateral view of thoracic segments **B** lateral view of abdominal segment I **C** lateral view of abdominal segments VII–X (schemes). Abbreviations: Th. 1–3–number of thoracic segments, Abd. 1–10–number of abdominal seg, setae: *as*–alar, *ds*–dorsal, *eps*–epipleural, *eus*–eusternal, *lsts*–laterosternal, *pda*–pedal, *pds*–postdorsal, *prns*–pronotal, *prs*–prodorsal, *ss*–spiracular, *ps*–pleural, *sts*–sternal.

**(Figs 54A, B, 55A–E, 56A–C). *Measurements*** (in mm). Body length: 4.60–7.00 (avg. 5.20). The widest place in the body (meso- and metathorax) measures up to 1.50. Head width: 0.62–0.75 (avg. 0.70).

***General*.** Body elongate, moderately slender, curved, rounded in cross section (Fig. 54A). All thoracic segments equal in size. Meso- and metathorax each divided dorsally into two folds (prodorsal fold distinctly smaller than postdorsal fold). Pedal folds of thoracic segments isolated, conical, and prominent. Abdominal segments I–III of similar size, next segments tapering towards posterior body end. Abdominal segments I–VII each divided dorsally into two almost equal in size folds; postdorsal folds only slightly higher than prodorsal folds. Segments VIII and IX dorsally undivided. Epipleural folds of segments I–VIII conical. Laterosternal and eusternal folds of segments I–VIII conical, weakly isolated. Abdominal segment X divided into four folds of equal size, lateral folds each with a single minute seta. Anus situated ventrally, almost completely covered by ninth segment.

Thoracic and abdominal spiracles unicameral; thoracic spiracles (Fig. 54A) placed laterally close to mesothorax; abdominal spiracles (Fig. 54A) placed medially on segments I–VIII.

***Colouration*.** Dark yellow to brown head, medial parts of epicranium less sclerotised (Fig. 54B). All thoracic and abdominal segments whitish, premental shield only slightly darker than the rest of prodorsum (Fig. 54A). Cuticle covered with asperities.

***Vestiture*.** Setae on body thin, yellowish, different in length (very short or medium).

***Head capsule*** (Figs 54B, 55A). Head suboval, endocarinal line present, reaching more than the 2/3 of the length of frons. Frontal sutures on head partially indistinct, wide. Frons covered with knobby, dark asperities. Single pair of stemmata in the form of small black spots (st) placed laterally, close to the end of the frontal suture. *Des_1_* short, located in middle part of epicranium; long *des_2_*; long *des_3_* located anteriorly, almost on the border of the frontal suture; minute *des_4_* located laterally; and long *des_5_* placed anterolaterally above stemma (Fig. 55A). *Fs_1_* short, located posterolaterally; *fs_2_* and *fs_3_* absent; *fs_4_* medium, located anteriorly; and long *fs_5_* located anterolaterally, close to antenna (Fig. 55A). *Les_1_* and *les_2_* medium; two short *ves*. Epicranial area with three *pes*.

***Antennae*** placed distally of the frontal suture, on the inside; membranous and distinctly convex basal article bearing one conical elongate sensorium, plus a single sensillum basiconicum and single sensillum ampullaceum (Fig. 55B).

***Clypeus*** (Fig. 55C) trapezoidal, ~ 3.5 × as wide as long with two medium *cls*, localised posterolaterally, with one sensillum between them; basolateral parts distinctly more sclerotised than the middle part; anterior border slightly curved towards the inside.

***Mouth parts*.** Labrum (Fig. 55C) ~ 2.5 × as wide as long, with three piliform *lrs*, variable in length; *lrs_1_* and *lrs_2_* elongated, located medially, *lrs_3_* short, located anterolaterally; anterior border bi-sinuate. Epipharynx (Fig. 55C) with three relatively elongated finger-like *als*, identical in length, three piliform *ams* variable in length and single short finger-like *mes*; labral rods (lr) elongated, close to kidney shaped. Mandibles (Fig. 55D) bifid, cutting edge smooth; two medium piliform and short *mds*, both located close to lateral border. Maxillolabial complex: maxilla brownish sclerotised (Fig. 55E), stipes with one *stps*, two *pfs* and one very short *mbs* and one sensillum, *stps* and both *pfs_1–2_* relatively long; mala with six finger-like *dms* variable in length; four piliform *vms*, medium to short in length. Maxillary palpi two-segmented; basal palpomere distinctly wider and slightly longer than distal one; basal palpomere with short *mpxs* and two sensilla, distal palpomere with a group of four apical sensilla in terminal receptive area. Prementum (Fig. 55E) close to oval-shaped, with one long *prms*; ligula with slightly sinuate margin and two medium *ligs*; premental sclerite weakly sclerotised, only lateral parts well visible. Labial palpi one-segmented; each palp with a single pore, and a group of three apical sensilla basiconica on terminal receptive area; surface of labium smooth. Postmentum (Fig. 55E) with three *pms*, medium *pms_1_* located anteromedially, long *pms_2_* located laterally, and medium *pms_3_* located anterolaterally; membranous area covered with knobby asperities.

***Thorax*.** Prothorax (Fig. 56A) with eight medium to short *prns*; two medium *ps*; and single short *eus*. Mesothorax (Fig. 56A) with one minute *prs*, two medium and one minute *pds* (ordered: minute, medium, medium); one medium *as*; two medium and single minute *ss*; one medium *eps*; two medium *ps*; and single minute *eus*. Chaetotaxy of metathorax (Fig. 56A) almost identical to that of mesothorax. Each pedal area of thoracic segments with four relatively long and one minute *pda*.

***Abdomen*.** Segments I–VIII (Fig. 56B, C) with one minute *prs* (segment VIII without); four *pds* of various length; one minute and one medium *ss*; three medium *eps*; one medium *ps*; one medium *lsts*; and two short *eus*. Abdominal segment IX (Fig. 56C) with one minute and one medium *ds*; one medium and one minute *ps*; and two minute *sts*.

#### Description of pupa

**Figure 57.**
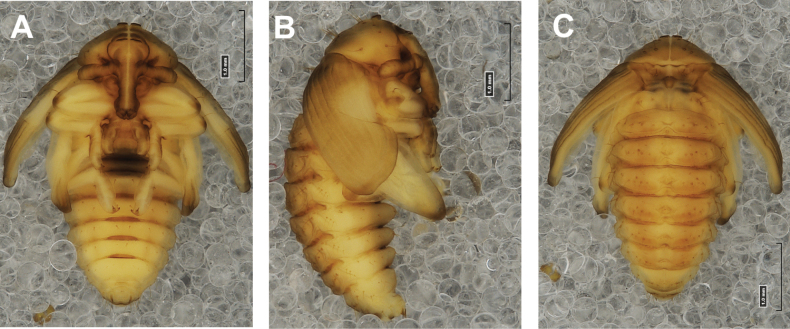
*Rhinusaneta* (Germar, 1821) pupa habitus **A** ventral view **B** lateral view **C** dorsal view.

**Figure 58.**
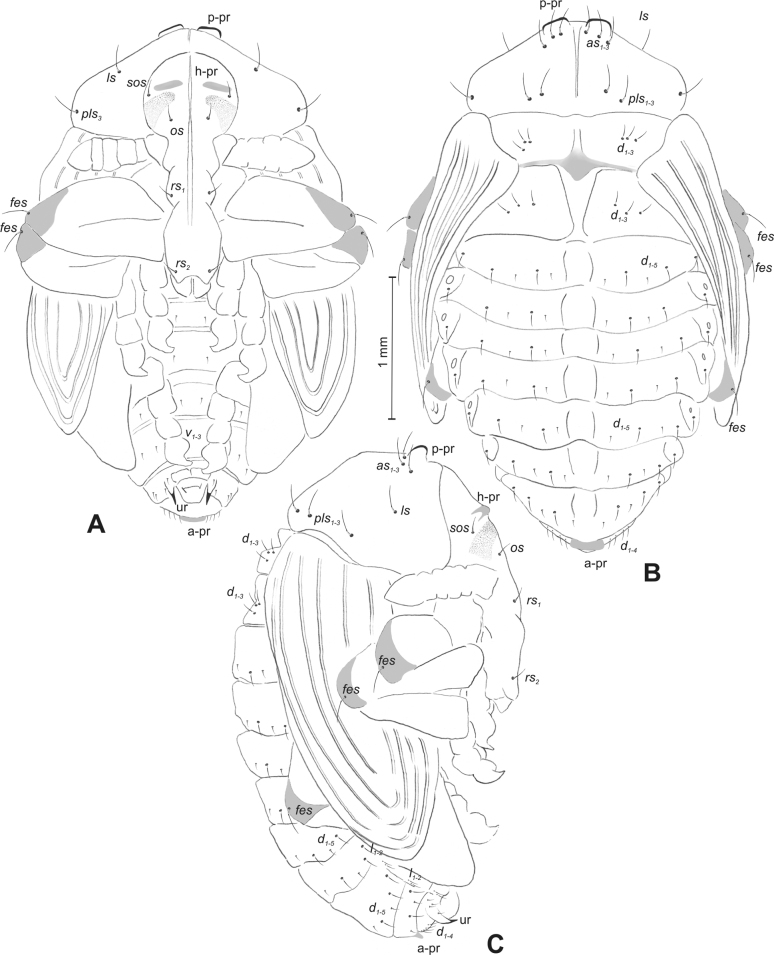
*Rhinusaneta* (Germar, 1821) pupa habitus **A** ventral view **B** dorsal view **C** lateral view (schemes). Abbreviations: a–pr–abdominal protuberances, h–pr–head protuberances, p–pr–pronotal protuberances, ur–urogomphi, setae: *as*–apical, *d*–dorsal, *ds*–discal, *fes*–femoral, *l*, *ls*–lateral, *os*–orbital, *pls*–posterolateral, *rs*–rostral, *sos*– supraorbital, *v*–ventral.

**(Figs 57A–C, 58A–C). *Measurements*** (in mm). Body length: 2.70–4.20 (avg. 3.25); body width: 1.53–2.26 (avg. 2.10); thorax width: 1.00–1.53 (avg. 1.40); rostrum length: up to 0.73 ♂ and 0.83 ♀.

***Body*.** Integument white, with some parts dark sclerotised; moderately stout, curved. All setae placed on dark brown spots. Head elongated protuberances present (h–pr) on head above eyes. Rostrum elongated, on both sexes almost 4 × as long as wide, reaching over mesocoxae. Pronotum trapezoidal 2.2 × as wide as long. Pronotal protuberances (p–pr) conical, flattened, sclerotised, separated at bases. Meso- and metanotum similar in size. Abdominal segments I–IV almost identical in size; segments V and VI tapering gradually, VII semicircular; segment VIII narrow; segment IX reduced. Abdominal segment VIII dorsally with flattened, weakly sclerotised abdominal protuberance (a–pr). Urogomphi (ur) short, ending with sclerotised, sharp apexes (Fig. 57A–C).

***Chaetotaxy*.** Well developed, setae medium to elongated, transparent. Head with one medium *sos* and one medium *os*. Rostrum with two *rs* (Fig. 58A). Pronotum with three *as*, single *ls*, and three *pls* all equal in length. Dorsal parts of meso- and metathorax with three setae of various length, placed medially. Apex of femora with a single long *fes* (Fig. 58A–C). Abdominal segments I–VII dorsally with five setae, variable in length: first and third minute, second, fourth, and fifth medium; setae first to fourth placed close to posterior margin of the segment, fifth placed below stigma (on segments VI and VII all setae medium in size). Abdominal segment VIII dorsally with four elongated setae dorsally. Each lateral part of abdominal segments I–VIII with two short setae. Ventral parts of abdominal segments I–VIII with three medium setae. Abdominal segment IX with three short setae ventrally (Fig. 58A–C).

#### Remarks and comparative notes.

This species is very common and has a wide distribution (western, central, and southern Europe, Caucasus, Iran, central Asia; Alonso-Zarazaga et al. 2023). It was accidentally introduced in North America (Buchanan 1937) but subsequently used as a biological control agent against the dalmatian toadflax (*Linariadalmatica*) and yellow toadflax (*Linariavulgaris*) (Sing et al. 2016). There are no noteworthy morphological differences between various populations living on different species of *Linaria*. The shape of the rostrum is the most useful character, which allows easy separation at adult stage of *R.neta* from the other species of the genus with short elytra, except for the western Mediterranean *R.incana*, from which it can be distinguished only by the shape of the female rostrum, the antennal insertion being located slightly more towards the apex of the rostrum in both sexes, and the scales of the dorsal vestiture being slightly stouter.

The descriptions of the larva and pupa of *R.neta* given by Ścibior and Łętowski (2018) are generally similar to ours, with some differences probably due to the nomenclature used by these authors. In our opinion, some setae were incorrectly identified in the larva, e.g., *as_1_* instead of *ls_1_*. There are also some discrepancies in the interpretation of pupal characters, such as a lack of *rs_2_* or *sos* instead of *os* and four abdominal dorsal setae instead of five.

#### Biological notes.

This is an oligophagous species whose larvae develop on different toadflaxes (*Linaria* spp.). The females oviposit eggs from mid-June to the end of September in an already developed seed capsule. The larvae are typical seed feeders. Larval development was recorded on many *Linaria* species: *L.vulgaris*, *L.genistifolia*, *L.dalmatica*, *L.grandiflora* Desf., *L.angustissima*, *L.arvensis* (L.) Desf., *L.corifolia* Desf., *L.peloponnesiaca* Boiss. and Heldr., *L.repens* (L.) Mill., *L.rubioides* Vis. & Pancic, *L.spartea* (L.) Chaz., *L.supina* (L.) Chaz., and *L.vulgaris*. Oviposition of eggs and larval feeding do not cause swelling of seeds, but larvae may consume a large proportion of seeds within a capsule, decreasing the seed output but not killing the host plant (Sing et al. 2016).

##### ﻿*Rhinusavestita* group

**Adult diagnosis.** Rostrum short and stout in both sexes; elytra subquadrate, distinctly flattened on disc; penis with short endophallus beginning just in front of basal third of its body.

### ﻿13) Rhinusa vestita

Taxon classificationAnimaliaColeopteraCurculionidae

(Germar, 1821)

E181D1E7-3113-5DA5-B15E-4D466CB7CAA8

#### Material examined.

21 mature and 7 premature larvae; 7 ♂ and 11 ♀ pupae. France, Alpes-Maritimes, La Turbie, ex *Antirrhinummajus* L., 01.06.2014, leg., det. R. Caldara.

#### Description of mature larva

**Figure 59.**
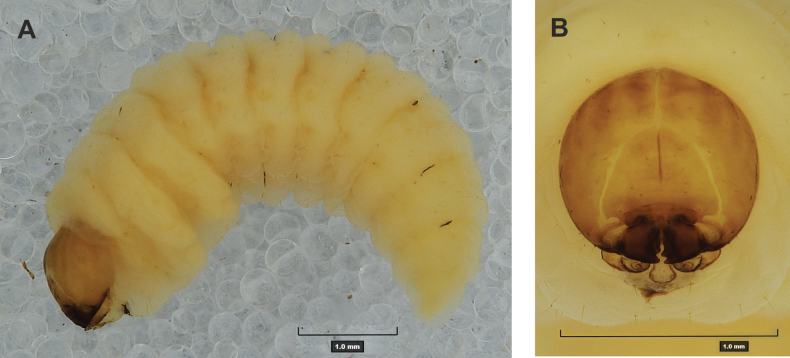
*Rhinusavestita* (Germar, 1821) mature larva **A** habitus **B** head, frontal view.

**Figure 60.**
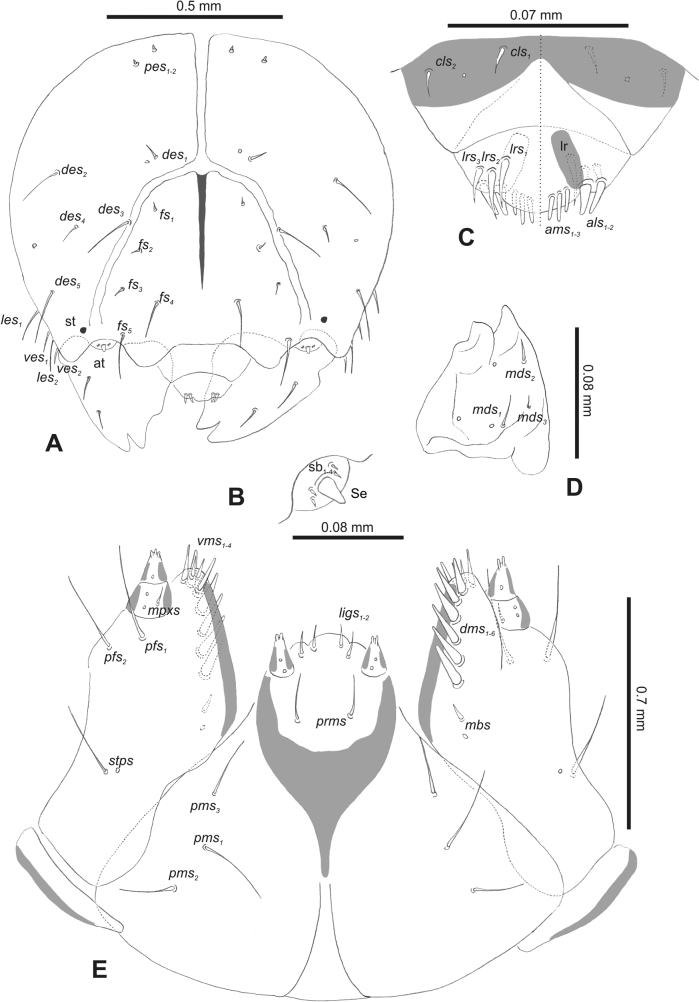
*Rhinusavestita* (Germar, 1821) mature larva, head and mouth parts **A** head **B** antenna **C** clypeus and labrum (left side), epipharynx (right side) **D** left mandible **E** maxillolabial complex (schemes). Abbreviations: at–antenna, lr–labral rods, sb–sensillum basiconicum, Se–sensorium, st–stemmata, setae: *als*–anterolateral, *ams*–anteromedial, *cls*–clypeal, *des*–dorsal epicranial, *dms*–dorsal malar, *fs*–frontal epicranial, *les*–lateral epicranial, *ligs*–ligular, *lrs*–labral, *mbs*–malar basiventral, *mds*–mandibular dorsal, *mpxs*–maxillary palp, *pes*–postepicranial, *pfs*–palpiferal, *pms*–postmental, *prms*–premental, *stps*–stipital, *ves*–ventral, *vms*–ventral malar.

**Figure 61.**
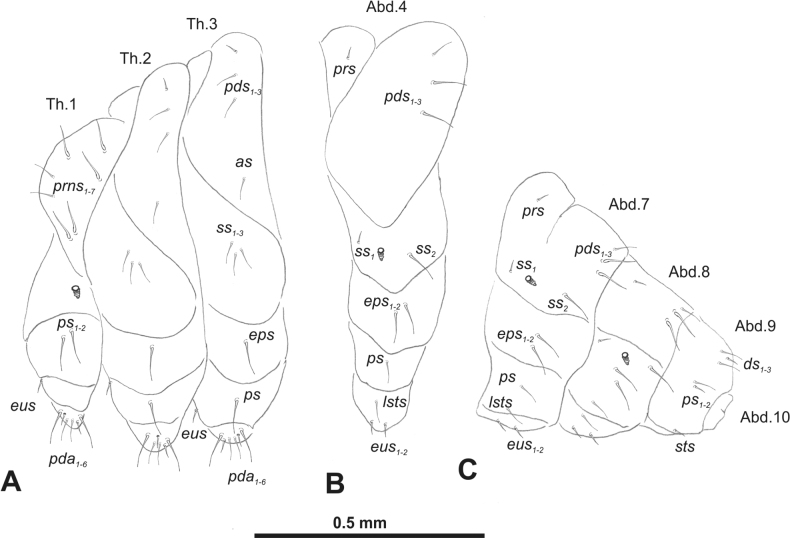
*Rhinusavestita* (Germar, 1821) mature larva, habitus **A** lateral view of thoracic segments **B** lateral view of abdominal segment I **C** lateral view of abdominal segments VII–X (schemes). Abbreviations: Th. 1–3–number of thoracic segments, Abd. 1–10–number of abdominal seg, setae: *as*–alar, *ds*–dorsal, *eps*–epipleural, *eus*–eusternal, *lsts*–laterosternal, *pda*–pedal, *pds*–postdorsal, *prns*–pronotal, *prs*–prodorsal, *ss*–spiracular, *ps*–pleural, *sts*–sternal.

**(Figs 59A, B, 60A–E, 61A–C). *Measurements*** (in mm). Body length: 6.50–8.25 (avg. 7.50). The widest place in the body (meso- and metathorax) measures up to 2.50. Head width: 0.90–1.05 (avg. 1.00).

***General*.** Body elongate, moderately slender, slightly curved, rounded in cross section (Fig. 59A). Prothorax relatively small, pronotal shield not pigmented; meso- and metathorax equal in size, distinctly wider than prothorax. Meso- and metathorax each divided dorsally into two folds (prodorsal fold vestigial, postdorsal fold prominent). Pedal folds of thoracic segments isolated, conical. Abdominal segments I–III of similar size, as large as metathorax; segment IV the widest; next segments tapering towards posterior body end. Abdominal segments I–VII each divided dorsally into two folds various in size; postdorsal folds larger than prodorsal folds. Segments VIII and IX dorsally undivided. Epipleural folds of segments I–VII conical. Laterosternal and eusternal folds of segments I–VIII conical, well isolated. Abdominal segment X divided into four folds of equal size. Anus situated ventrally, almost completely hidden in previous segment.

All spiracles unicameral, thoracic spiracles (Fig. 59A) placed ventrolaterally; abdominal spiracles (Fig. 59A) placed anteromedially on segments I–VIII.

***Colouration*.** Dark yellow to light brown head, medial parts of epicranium less sclerotised (Fig. 59B). All thoracic and abdominal segments white (Fig. 59A). Cuticle densely covered with cuticular asperities.

***Vestiture*.** Setae on body well developed, yellowish, different in length (minute to medium).

***Head capsule*** (Figs 59B, 60A). Head wide, endocarinal line present, reaching to the 3/4 of the length of frons. Frontal sutures on head distinct, very wide. Single pair of stemmata in the form of small black spots (st) close to the end of the frontal suture. *Des_1_* short, located in middle part of epicranium; long *des_2_*; long *des_3_* located anteriorly on epicranium, close to border with frontal suture; *des_4_* short; *des_5_* elongated, located anterolaterally above stemma (Fig. 60A). *Fs_1_*, *fs_2_* and *fs_3_* minute, located along frontal suture; *fs_4_* long, located anteriorly; and long *fs_5_* located anterolaterally, close to antenna (Fig. 60A). *Les_1_* and *les_2_* medium; two minute *ves*. Epicranial area with two *pes*.

***Antennae*** placed distally of the frontal suture, on the inside; membranous and distinctly convex basal article bearing one conical, medium in length sensorium, plus four sensilla ampullacea (Fig. 60B).

***Clypeus*** (Fig. 60C) trapezoidal, ~ 2.7 × as wide as long with two *cls*: *cls_1_* relatively long, *cls_2_* medium, both localised posterolaterally, with one sensillum between them; basal part distinctly more sclerotised than the apical part; anterior border slightly curved towards the inside.

***Mouth parts*.** Labrum (Fig. 60C) ~ 2 × as wide as long, with three piliform *lrs*, various long; *lrs_1_* elongated, located posteromedially, *lrs_2_* elongated, located medially, and *lrs_3_* short, located anterolaterally; anterior border bisinuate. Epipharynx (Fig. 60C) with two elongated finger-like *als*, almost identical in length and three piliform *ams* equally in length; labral rods (lr) distinct, elongated, converging posteriorly. Mandibles (Fig. 60D) bifid, cutting edge with a single, blunt protuberance; two medium piliform and single minute *mds*, all located close to lateral border. Maxillolabial complex: maxilla dark sclerotised (Fig. 60E) stipes with one *stps*, two *pfs* and one short *mbs* and one sensillum, *stps* and both *pfs_1–2_* elongated; mala with six finger-like *dms* variable in length; four piliform *vms*, medium to short in length. Maxillary palpi two-segmented; basal palpomere distinctly wider than distal one; length ratio of basal and distal palpomeres almost 1:1; basal palpomere with short *mpxs* and two sensilla, distal palpomere with a group of three or four apical sensilla in terminal receptive area. Prementum (Fig. 60E) cup-shaped, with one medium *prms*; ligula with sinuate margin and two medium *ligs*; premental sclerite broad, highly sclerotised, Y-shaped, posterior extension with elongated apex. Labial palpi one-segmented; palpi with two pores, and a group of three or four apical sensilla (ampullacea) on terminal receptive area; surface of labium smooth. Postmentum (Fig. 60E) with three *pms*, medium *pms_1_* located posteromedially, elongated *pms_2_* located laterally, and medium *pms_3_* located anterolaterally; membranous area smooth.

***Thorax*.** Prothorax (Fig. 61A) with five elongated and two medium *prns*: five placed apically, next two laterally; two elongated *ps*; and single short *eus*. Mesothorax (Fig. 61A) with one short and two medium *pds* (ordered: short, medium, medium); one medium *as*; three medium *ss*; one elongated *eps*; one elongated *ps*; and single minute *eus*. Chaetotaxy of metathorax (Fig. 61A) almost identical to that of mesothorax. Each pedal area of thoracic segments with two elongated, three medium and one minute *pda*.

***Abdomen*.** Segments I–VIII (Fig. 61B, C) with one minute *prs*; one medium and two elongated *pds*; one minute and one elongated *ss*; two elongated *eps*; one medium *ps*; one medium *lsts*; and two minute *eus*. Abdominal segment IX (Fig. 61C) with two medium and single minute *ds*; one medium and one minute *ps*; and single minute *sts*.

#### Description of pupa

**Figure 62.**
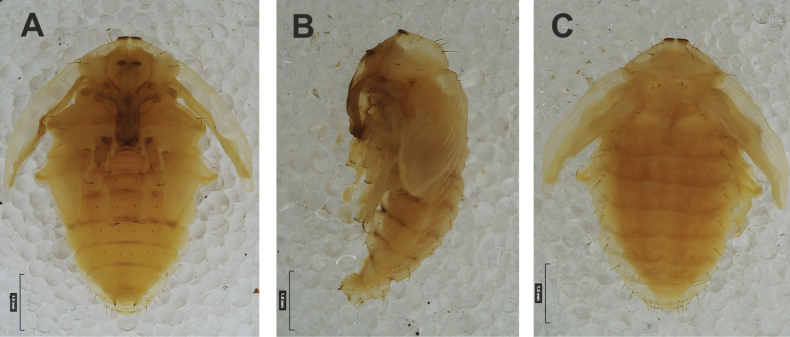
*Rhinusavestita* (Germar, 1821) pupa habitus **A** ventral view **B** lateral view **C** dorsal view.

**Figure 63.**
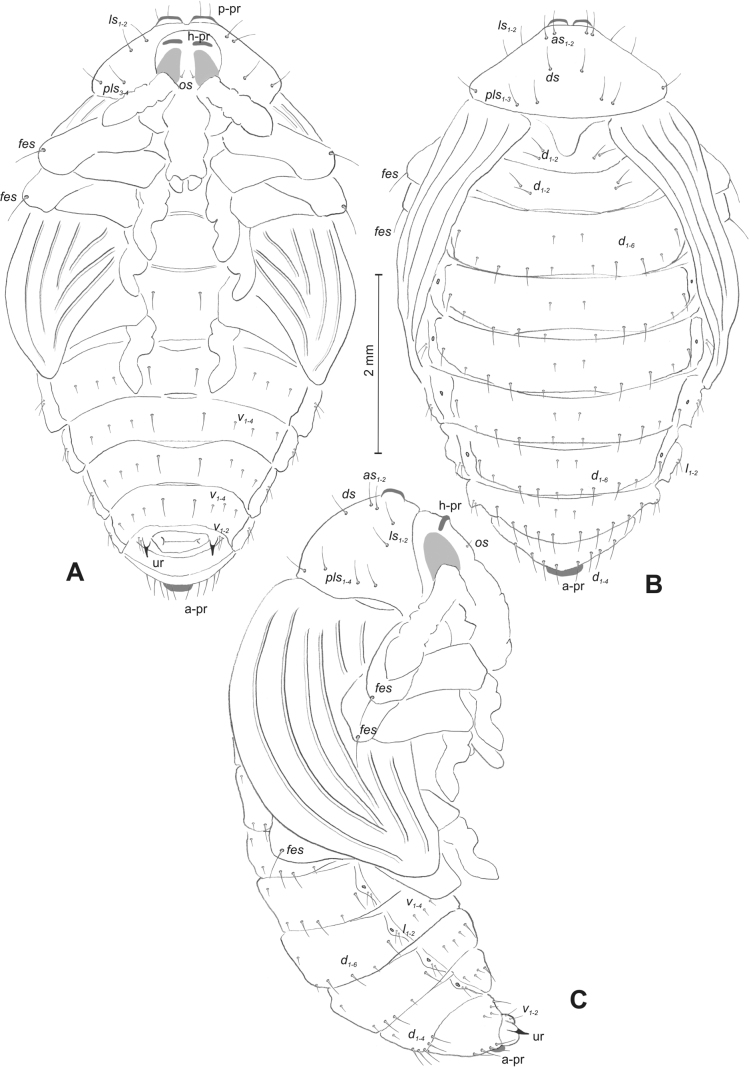
*Rhinusavestita* (Germar, 1821) pupa habitus **A** ventral view **B** dorsal view **C** lateral view (schemes). Abbreviations: p–pr–pronotal protuberances, ur–urogomphi, setae: *as*–apical, *d*–dorsal, *ds*–discal, *fes*–femoral, *l*, *ls*–lateral, *os*–orbital, *pls*–posterolateral, *v*–ventral.

**(Figs 62A–C, 63A–C). *Measurements*** (in mm). Body length: 4.75–6.50 (avg. 5.25); body width: 2.75–3.50 (avg. 3.15); thorax width: 1.60–2.00 (avg. 1.90); rostrum length: up to 1.25 ♂ and ♀.

***Body*.** Integument white or light yellow, moderately elongated, slightly curved. Head with a pair of small head protuberances (h–pr) above eyes. Rostrum moderately stout, almost 2.5 × as long as wide, reaching mesocoxae, on both sexes. Pronotum trapezoidal 2.0 × as wide as long. Pronotum with a pair of conical, sclerotised, protuberances (p–pr) separated at bases. Meso- and metanotum similar in size. Abdominal segments I–VI almost identical in size; segment VII semicircular; segment VIII narrow with broad protuberances (a–pr); segment IX reduced. Urogomphi (ur) small, ending with sclerotised, sharp apexes (Fig. 62A–C).

***Chaetotaxy*.** Well developed, setae medium to elongated, transparent. Head with one small *os* (Fig. 63A). Pronotum with two *as*, one *ds*, two *ls*, and four *pls* all medium in length. Dorsal parts of meso- and metathorax with two equal in length setae, placed medially. Apex of femora with a single elongated *fes* (Fig. 63A–C). Abdominal segments I–V with six setae dorsally, variable in length: first, second and fifth minute, third, fourth, and sixth medium, first placed anteromedially, the rest placed close to posterior margin of the segment. Abdominal segment VI with six setae dorsally, variable in length: first minute, second to sixth elongated, first placed anteromedially, second to sixth placed close to posterior margin of the segment. Abdominal segment VII with four elongated setae dorsally. Each lateral part of abdominal segments I–VII with two medium setae. Ventral parts of abdominal segments I–VIII with four setae (median pair robust, second to fourth short). Abdominal segment IX with two short setae ventrally (Fig. 63A–C).

#### Remarks and comparative notes.

This species is known from Spain, Portugal, southern France, Switzerland, and northwestern and central Italy (Alonso-Zarazaga et al. 2023). At the adult stage, due to the medium-large size and the broad subquadrate shape of the elytra, which are almost flattened on the disc, this species is similar to *R.depressa* (Rottenberg, 1872) and *R.fuentei* (Pic, 1906). From the former, with which it is also closely related phylogenetically, it can be mainly distinguished by the shape and the greater length of the rostrum and of the penis; from the latter, which belongs to the *R.antirrhini* group, it distinctly differs in the shape of the rostrum and obviously in the characters that easily distinguish the *R.vestita* group from the *R.antirrhini* group (femora strongly toothed, shape of the penis; Caldara et al. 2010).

#### Biological notes.

The female usually oviposits 1–3 eggs per seed capsule. Egg hatching occurs 7–11 days after deposition. Larvae feed on seeds within capsules of *Antirrhinummajus* and *A.latifolium* Mill., consuming the majority of them. Pupation occurs within the seed capsule, and the emergence of the adults occurs ~ 20 days later. Adults exit the seed capsule by chewing through the hardened pericarp (IT and RC, pers. obs.).

##### ﻿*Rhinusamelas* group

**Adult diagnosis.** Rostrum poorly sexually dimorphic and short in both sexes; elytra short; body of penis abruptly narrowing toward apex ending in a narrow acute point; flagellum distinctly sclerotised, sinuous in its apical section; spermatheca with distinct emargination at passage point between nodus and body.

### ﻿14) Rhinusa melas

Taxon classificationAnimaliaColeopteraCurculionidae

(Boheman, 1838)

3F2F15FB-87DE-52DA-ACAD-3674583ED251

#### Material examined.

11 mature and 5 premature larvae; 8 ♂ and 6 ♀ pupae. Serbia, Mokra Gora, ex *Chaenorhinumminus* (L.) Lange, 10.08.2017, leg., det. I. Toševski.

#### Description of mature larva

**Figure 64.**
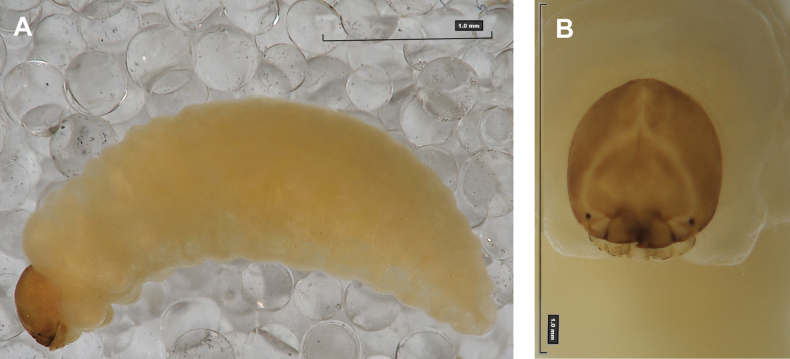
*Rhinusamelas* (Boheman, 1838) mature larva **A** habitus **B** head, frontal view.

**Figure 65.**
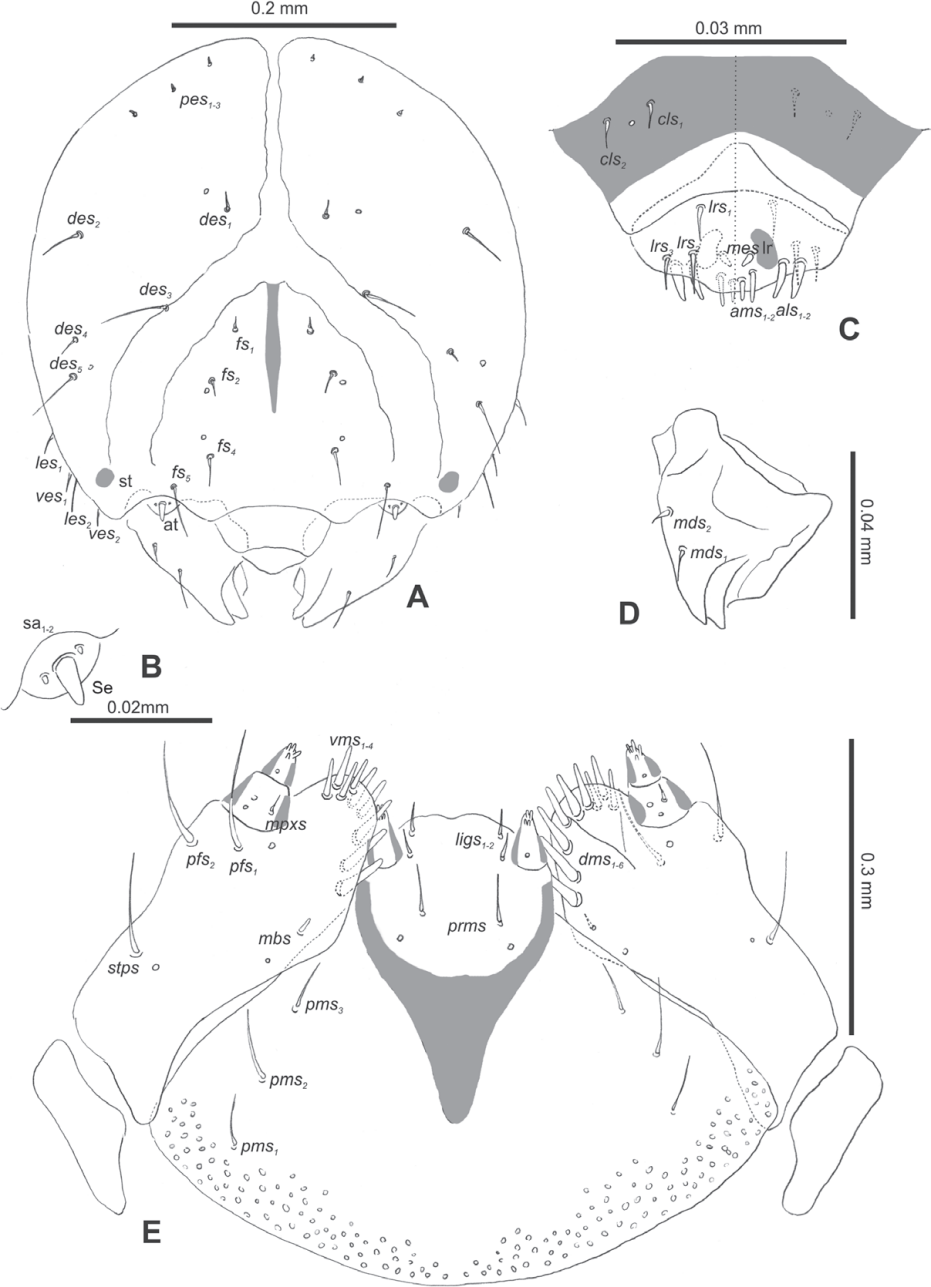
*Rhinusamelas* (Boheman, 1838) mature larva, head and mouth parts **A** head **B** antenna **C** clypeus and labrum (left side), epipharynx (right side) **D** left mandible **E** maxillolabial complex (schemes). Abbreviations: at–antenna, lr–labral rods, sa–sensillum ampullaceum, Se–sensorium, st–stemmata, setae: *als*–anterolateral, *ams*–anteromedial, *cls*–clypeal, *des*–dorsal epicranial, *dms*–dorsal malar, *fs*–frontal epicranial, *les*–lateral epicranial, *ligs*–ligular, *lrs*–labral, *mbs*–malar basiventral, *mds*–mandibular dorsal, *mes*–medial, *mpxs*–maxillary palp, *pes*–postepicranial, *pfs*–palpiferal, *pms*–postmental, *prms*–premental, *stps*–stipital, *ves*–ventral, *vms*–ventral malar.

**Figure 66.**
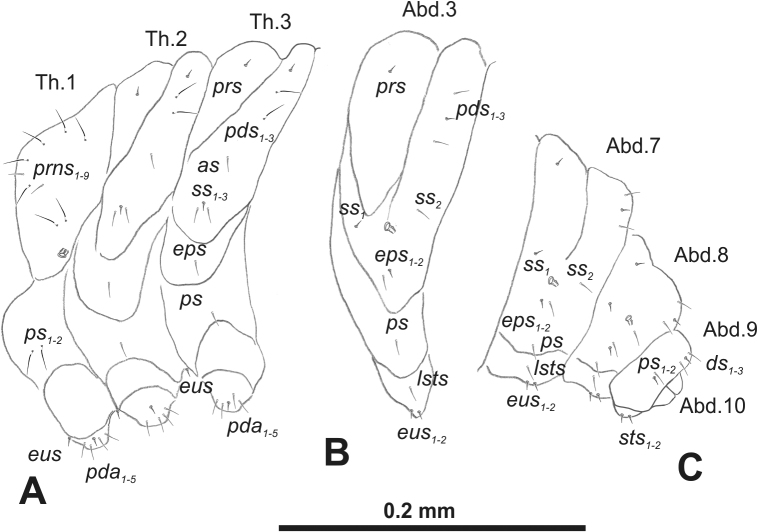
*Rhinusamelas* (Boheman, 1838) mature larva, habitus **A** lateral view of thoracic segments **B** lateral view of abdominal segment I **C** lateral view of abdominal segments VII–X (schemes). Abbreviations: Th. 1–3–number of thoracic segments, Abd. 1–10–number of abdominal seg, setae: *as*–alar, *ds*–dorsal, *eps*–epipleural, *eus*–eusternal, *lsts*–laterosternal, *pda*–pedal, *pds*–postdorsal, *prns*–pronotal, *prs*–prodorsal, *ss*–spiracular, *ps*–pleural, *sts*–sternal.

**(Figs 64A, B, 65A–E, 66A–C). *Measurements*** (in mm). Body length: 2.50–3.66 (avg. 2.83). The widest place in the body (meso- and metathorax) measures up to 1.16. Head width: 0.43–0.50 (avg. 0.46).

***General*.** Body elongate, slender, moderately curved, rounded in cross section (Fig. 64A). All thoracic segments equal in size. Meso- and metathorax each divided dorsally into two folds (prodorsal fold very small, postdorsal fold prominent). Pedal folds of thoracic segments conical. Abdominal segments I–V of similar size, next segments tapering towards posterior body end. Abdominal segments I–VII each divided dorsally into two folds almost identical in size. Segments VIII and IX dorsally undivided. Epipleural folds of segments I–VIII conical. Laterosternal and eusternal folds of segments I–VIII conical, poorly isolated. Abdominal segment X divided into four folds of equal size. Anus situated ventrally, almost completely hidden in segment IX.

Thoracic spiracle bicameral, all abdominal spiracles unicameral; thoracic spiracle (Fig. 64A) placed laterally close to mesothorax; abdominal spiracles (Fig. 64A) placed medially on segments I–VIII.

***Colouration*.** Light to dark yellow head (Fig. 64B). All thoracic and abdominal segments whitish (Fig. 64A). Cuticle covered with asperities.

***Vestiture*.** Setae on body thin, transparent, different in length (minute to medium).

***Head capsule*** (Figs 64B, 65A). Head suboval, endocarinal line present, reaching to 1/2 of the length of frons. Frontal sutures on head indistinct, very wide. Single pair of stemmata in the form of small dark spots (st) placed laterally, close to the end of the frontal suture. *Des_1_* short, located in middle part of epicranium; long *des_2_* located anteriorly; long *des_3_* placed almost on the border of the frontal suture; very short *des_4_* located laterally; very long *des_5_* placed anterolaterally above stemma (Fig. 65A). *Fs_1_* minute, placed posteriorly; *fs_2_* minute, located posterolaterally; *fs_3_* absent; *fs_4_* medium; and long *fs_5_* located anterolaterally, close to antenna (Fig. 65A). *Les_1_* medium and *les_2_* short; two short *ves*. Epicranial area with three *pes*.

***Antennae*** placed distally of the frontal suture, on the inside; membranous and distinctly convex basal article bearing one conical elongate sensorium, plus two sensilla ampullacea (Fig. 65B).

***Clypeus*** (Fig. 65C) trapezoidal, ~ 2.5 × as wide as long with two medium *cls*, localised posterolaterally, with one sensillum between them; anterior part distinctly less sclerotised than the basal part; anterior border slightly rounded towards the inside.

***Mouth parts*.** Labrum (Fig. 65C) ~ 2.2 × as wide as long, with three piliform *lrs*, various long; *lrs_1_* and *lrs_2_* elongated, located medially, and *lrs_3_* medium, located anterolaterally; anterior border bi-sinuate. Epipharynx (Fig. 65C) with two elongated finger-like *als*, identical in length; two piliform *ams* variable in length, and single finger-like *mes*; labral rods (lr) distinct, kidney-shaped. Mandibles (Fig. 65D) bifid, cutting edge with small additional teeth; two medium piliform and short *mds*, both located close to lateral border. Maxillolabial complex: maxilla brownish sclerotised (Fig. 65E) stipes with one *stps*, two *pfs* and one very short *mbs*, *stps* and both *pfs_1–2_* elongated; mala with six finger-like *dms* variable in length; four medium piliform *vms*. Maxillary palpi two-segmented; basal palpomere distinctly wider than distal one; basal palpomere with short *mpxs* and two sensilla, distal palpomere with a group of four apical sensilla in terminal receptive area. Prementum (Fig. 65E) close to cup-shaped, with a single, medium *prms*; ligula with slightly sinuate margin and two short *ligs*; premental sclerite broad, sclerotised, posterior extension with elongated apex. Labial palpi one-segmented; palpi with a single pore, and four or five apical sensilla in terminal receptive area; surface of labium smooth. Postmentum (Fig. 65E) with three *pms*, medium *pms_1_* located posteromedially, long *pms_2_* located mediolaterally, and medium *pms_3_* located anterolaterally; membranous area partially covered with knobby asperities.

***Thorax*.** Prothorax (Fig. 66A) with seven elongated and two medium *prns*; two medium *ps*; and single short *eus*. Mesothorax (Fig. 66A) with a single minute *prs*; three *pds* (ordered: minute, medium and medium); one medium *as*; two medium and one minute *ss*; one medium *eps*; one medium *ps*; and single minute *eus*. Chaetotaxy of metathorax (Fig. 66A) almost identical to that of mesothorax. Each pedal area of thoracic segments with four medium and single minute *pda*.

***Abdomen*.** Segments I–VIII (Fig. 66B, C) with a single minute *prs*; three *pds* of various length; one minute and one medium *ss*; one medium and one minute *eps*; one medium *ps*; one medium *lsts*; and two minute *eus*. Abdominal segment IX (Fig. 66C) with two medium and one minute *ds*; one medium and one minute *ps*; and two minute *sts*.

#### Description of pupa

**Figure 67.**
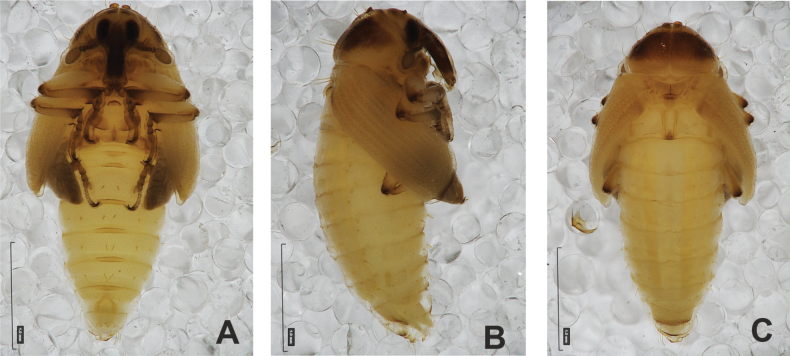
*Rhinusamelas* (Boheman, 1838) pupa habitus **A** ventral view **B** lateral view **C** dorsal view.

**Figure 68.**
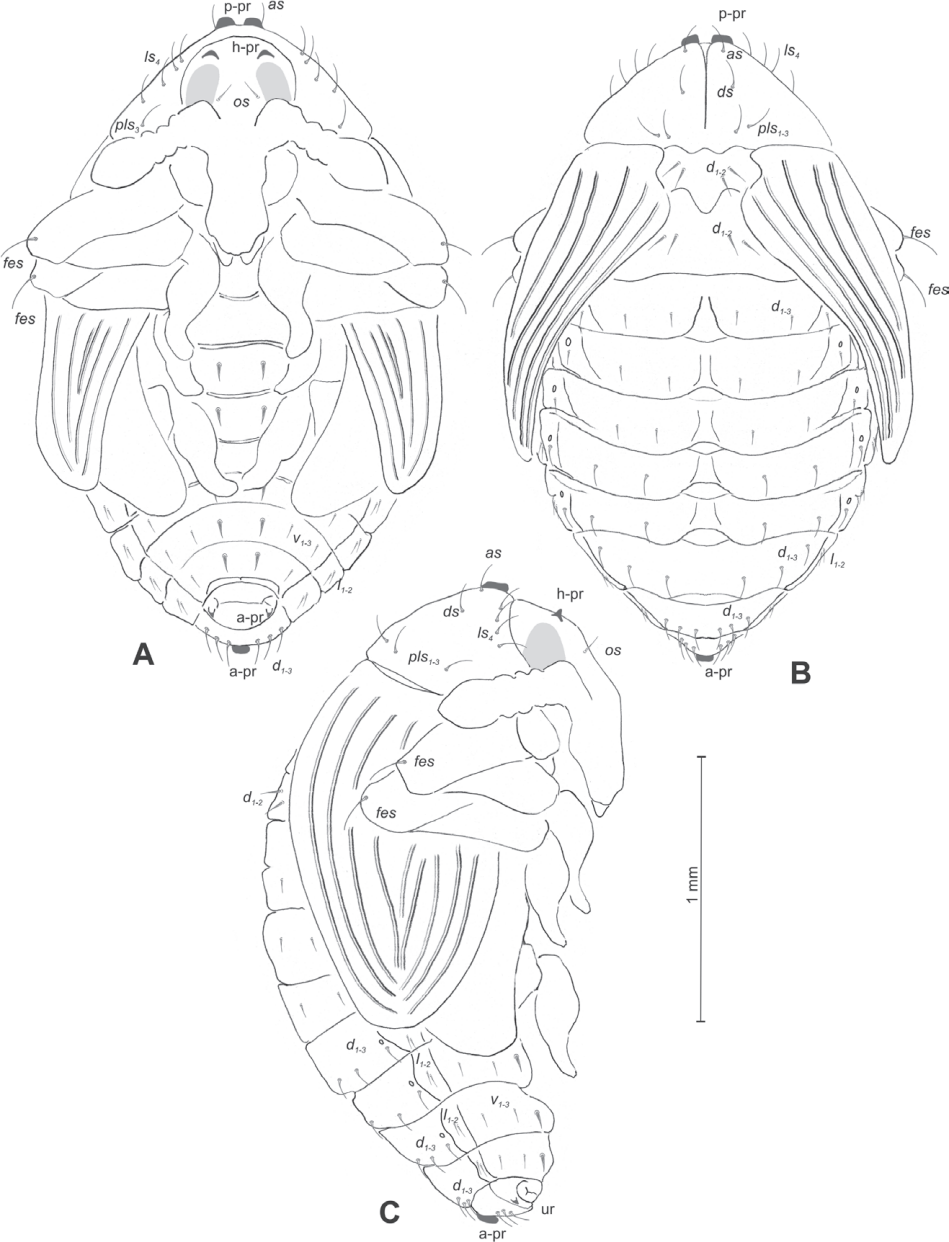
*Rhinusamelas* (Boheman, 1838) pupa habitus **A** ventral view **B** lateral view **C** dorsal view (schemes). Abbreviations: a–pr–abdominal protuberances, h–pr–head protuberances, p–pr–pronotal protuberances, ur–urogomphi, setae: *as*–apical, *d*–dorsal, *ds*–discal, *fes*–femoral, *l*, *ls*–lateral, *os*–orbital, *pls*–posterolateral, *v*–ventral.

**(Figs 67A–C, 68A–C). *Measurements*** (in mm). Body length: 2.30–2.90; body width: 0.80–1.75; thorax width: 0.90–1.05; rostrum length: up to 0.75 ♂ and ♀.

***Body*.** Integument white; moderately elongated. Head and pronotum with protuberances. Rostrum rather short, reaching to mesocoxae; on both sexes almost 2.2 × longer than wider. Pronotum trapezoidal 2 × as wide as long. Meso- and metanotum similar in size. Abdominal segments I–IV almost identical in size; segments V and VI tapering gradually, VII semicircular; segment VIII narrow; segment IX reduced. Abdominal segment VIII with, semicircular, weakly sclerotised abdominal protuberance (a–pr). Urogomphi vestigial, weakly sclerotised (Fig. 67A–C).

***Chaetotaxy*.** Well-developed setae, elongated to short, dark brown. Head with a single short *os*. Rostrum without seta (Fig. 68A). Pronotum with a single *as*, single *ds*, four *ls*, and three *pls*; all pronotal setae elongated, equal in length. Dorsal parts of meso- and metathorax with two elongated setae, placed medially (Fig. 68A–C). Abdominal segments I–VIII dorsally with three medium (on segments VII and VIII elongated) setae dorsally, placed close to posterior margin of the segments. Each lateral part of abdominal segments I–VIII with one short seta. Ventral parts of abdominal segments I–VIII with three short setae, of which medial are robust, almost thorn-like. Abdominal segment IX without seta ventrally (Fig. 68A–C).

#### Remarks and comparative notes.

This species is widely distributed, although uncommon, in southern, western, and central Europe (Alonso-Zarazaga et al. 2023). The apparent presence only in southern Spain and in the Pyrenees of adults of a form with a reddish elytral integument together with specimens with black elytra is unusual, although not unique in the genus *Rhinusa*. In fact, the same occurs in *R.bipustulata* (Rossi, 1792) and *R.tetra*, which live on *Scrophularia* and *Verbascum* and are unrelated to *R.melas*. It will surely be very interesting to confirm this distributional pattern through a molecular study.

#### Biological notes.

The host plant of *R.melas* is *Chaenorhinumminus*. Larvae develop in the seed capsules, where they pupate (Hoffmann 1958; Smreczyński 1976). Adults were also collected on *Linariavulgaris*, *L.repens* and *L.spartea*, but most likely as occasional visitors.

##### ﻿Key to mature larvae of selected *Rhinusa* species

The following key is for the larvae of 14 *Rhinusa* species treated in this paper plus one species (*R.bipustulata*) in a previously published paper (Gosik 2010).

**Table:** 

1	Pronotal spiracle bicameral (Figs 13A, 18A, 66A)	**2**
–	Pronotal spiracle unicameral (Figs 3A, 8A, 23A, 28A, 33A, 38A, 43A, 48A, 53A, 56A, 61A)	**4**
2	Endocarinal line reaching 1/2 of length of frons (Fig. 65A). Postmentum partially covered with knobby asperities (Fig. 65E). Premental sclerite broad, well sclerotised, its posterior extension elongated (Fig. 65E). Mala with 6 *dms* (Fig. 65E)	***R.melas***
–	Endocarinal line reaching < 2/3 of the length of the frons (Figs 12A, 17A). Postmentum smooth (Figs 12E, 17E). Premental sclerite weakly sclerotised, its posterior extension vestigial (Figs 12E, 17E). Mala with 5 *dms* (Figs 12E, 17E)	**3**
3	*Des_1_* present, *des_4_* absent (Fig. 17A). Antenna with 3 sensilla basiconica (Fig. 17B). *Cls* very long (Fig. 17C). Ligula with 3 *ligs* (Fig. 17E). Mala with 5 *vms* (Fig. 17E). Pronotum with 12 *prns* (Fig. 18A). Abdominal segments I–VIII with 2 *pds* (Fig. 18B, C)	***R.florum***
–	*Des_1_* absent, *des_4_* present (Fig. 12A). Antenna with 1 sensillum basiconicum and 4 sensilla ampullacea (Fig. 12B). *Cls* very short (Fig. 12C). Ligula with 2 *ligs* (Fig. 12E). Mala with 4 *vms* (Fig. 12E). Pronotum with 10 *prns* (Fig. 13A). Abdominal segments I–VIII with 3 *pds* (Fig. 12B, C)	***R.antirrhini***
4	Frons covered with knobby protuberances (Figs 42A, 47A, 52A, 55A)	**5**
–	Frons smooth (Figs 2A, 7A, 22A, 27A, 32A, 37A, 60A)	**8**
5	Pronotum with 5 *prns* (Fig. 43A). Abdominal segments I–VIII with 3 *pds* and single *eps* (Fig. 43B, C)	***R.collina***
–	Pronotum with 7 or more *prns* (Figs 48A, 53A, 56A). Abdominal segments I–VIII with 4 *pds* and 3 *eps* (Figs 48B, C, 53B, C, 56B, C)	**6**
6	Postmentum smooth (Fig. 52E). *Fs_2_* and *fs_3_* present (Fig. 52A)	***R.incana***
–	Postmentum partially covered with knobby asperities (Figs 47E, 55E). *Fs_2_* and *fs_3_* absent (Figs 47A, 55A)	**7**
7	*Des_1_* short (Fig. 55A). Epipharynx with 3 *ams* (Fig. 55C). Mala with 6 *dms* (Fig. 55E). Premental sclerite without sclerotisation (Fig. 55E). Pronotum with 7 *prns* (Fig. 56A). Meso- and metathoracic segments with 3 *pds* (Fig. 56A). Abdominal segment IX with 2 *ds* (Fig. 56B, C)	***R.neta***
–	*Des_1_* elongate (Fig. 47A). Epipharynx with 2 *ams* (Fig. 47C). Mala with 5 *dms* (Fig. 47E). Premental sclerite highly sclerotised (Fig. 47E). Pronotum with 12 *prns* (Fig. 48A). Meso- and metathoracic segments with 4 *pds* (Fig. 48A). Abdominal segment IX with 3 *ds* (Fig. 47B, C)	***R.eversmanni***
8	Posterior extension of premental sclerite very elongated (Fig. 60E). Frons with 5 *fs* (Fig. 60A)	***R.vestita***
–	Posterior extension of premental sclerite very short, vestigial or even absent (Figs 2E, 7E, 22E, 27E, 32E, 37E). Frons with 3 or 4 *fs* (Figs 2A, 7A, 22A, 27A, 32A, 37A)	**9**
9	Clypeus and labrum fused, anterior margin almost straight (Figs 22C, 27C, 32C). Premental sclerite weakly sclerotised, posterior extension absent (Figs 22E, 27E, 32E). Mala wit 4 *dms* (Figs 22E, 27E, 32E). Postdorsal folds of abdominal segments I–VI distinct, conical, much higher than prodorsal folds (Figs 23B, 28B, 33B)	**10**
–	Clypeus and labrum separated, anterior margin of labrum round or sinuate (Figs 2C, 7C, 37C). Premental sclerite well sclerotised, posterior extension present (Figs 2E, 7E, 37E). Mala with 5–6 *dms* (Figs 2E, 7E, 37E). Postdorsal folds of abdominal segments I–VI as high as prodorsal folds dorsally (or only slightly higher) (Figs 3B, 8B, 38B)	**12**
10	Labial palpi well developed and protruding past the outline of the prementum (Fig. 32E). Ligula with 3 *ligs* (Fig. 32E). Pronotum with 4 *prns* (Fig. 33A). Each pedal area with 1 elongated and 3 minute setae (Fig. 33A). Meso-, metathorax and abdominal segments I–VIII with 1 *prs* (Fig. 33A–C). All abdominal setae minute, weakly visible (Fig. 33B, C). Abdominal segments I–VIII with 3 *pds* (Fig. 33B, C)	***R.rara***
–	Labial palpi small or vestigial, not protruding past the outline of the prementum (Figs 22E, 27E). Ligula with 2 *ligs* (Figs 22E, 27E). Pronotum with more than 7 *prns* (Figs 23A, 28A). Each pedal area with 5 setae of various lengths (Figs 23A, 28A). Meso-, metathorax and abdominal segments I–VIII without *prs* (Figs 23A–C, 28A–C). All abdominal setae well visible, variable in length (Figs 23B, C, 28B, C). Abdominal segments I–VIII with 1 or 2 *pds* (Figs 23B, C, 28B, C)	**11**
11	Labial palpi vestigial, almost invisible (Fig. 22E). Ligula with 1 *ligs* (Fig. 22E). Postmentum partially covered with knobby asperities (Fig. 22E). Clypeus with 1 *cls* (Fig. 22C). *Ams* and *als* almost identical in size (Fig. 22C). Sensorium elongated (Fig. 22B). Thoracic and abdominal segments without *eps* (Fig. 23A–C). Meso- and metathorax with 1 *pds* (Fig. 23A). Abdominal segments I–VIII with 1 *ss* and 1 *eus* (Fig. 23B, C)	***R.linariae***
–	Labial palpi small, but still visible (Fig. 27E). Ligula with 2 *ligs* (Fig. 27E). Postmentum smooth (Fig. 27E). Clypeus with 2 *cls* (Fig. 27C). *Als* finger-like and distinctly larger than small and piliform *ams* (Fig. 27C). Sensorium rather stout (Fig. 27B). Thoracic and abdominal segments with 1 *eps* (Fig. 28A–C). Meso- and metathorax with 2 *pds* (Fig. 28A). Abdominal segments I–VIII with 2 *ss* and 2 *eus* (Fig. 28B, C)	***R.pilosa***
12	Prementum with 2 *prms* (Fig. 37E). Epipharynx without *mes* (Fig. 37C). Labral rods round (Fig. 37C). Endocarinal line reaching to 2/3 of the frons (Fig. 37A). Pronotum with 12 *prns* (Fig. 38A). Abdominal segments I–VIII without *prs* and with 4 *pds* (Fig. 38B, C)	***R.herbarum***
–	Prementum with 1 *prms* (Figs 2E, 7E). Epipharynx with 1 or 2 *mes* (Figs 2C, 7C). Labral rods elongated or kidney-shaped (Figs 2C, 7C). Endocarinal line reaching to 1/2 of frons (Figs 2A, 7A). Pronotum with 6–9 *prns* (Figs 3A, 8A). Abdominal segments I–VIII with 1–2 *prs* and with 1–3 *pds* (Figs 3B, C, 8B, C)	**13**
13	Epipharynx with 2 *als* (Fig. 7C). Postmentum partially covered with knobbly asperities (Fig. 7E)	***R.tetra***
–	Epipharynx with 3 *als* (Fig. 2C). Postmentum smooth (Fig. 2E)	**14**
14	Head with 2 pairs of stemmata (Fig. 2A). Antenna with 2 sensilla basiconica and 2 styloconica (Fig. 2B). Posterior extension of premental sclerite moderately elongated (Fig. 2E). Ligula with 3 *ligs* (Fig. 2E). Abdominal segment IX without *ds* (Fig. 3C)	***R.asellus***
–	Head with single pair of stemmata. Antenna with 7 sensilla styloconica. Posterior extension of premental sclerite short. Ligula with 2 *ligs*. Abdominal segment IX with 1 *ds*	***R.bipustulata***

##### ﻿Key to pupae of selected *Rhinusa* species

The following key is for the pupae described in this paper for 13 *Rhinusa* species plus the pupa of one species (*R.bipustulata*), described in a previously published paper (Gosik 2010).

**Table:** 

1	Pronotal and abdominal protuberances absent (Figs 30A–C, 35A–C)	**2**
–	Pronotal and/or abdominal protuberances present (Figs 5A–C, 10A–C, 15A–C, 20A–C, 25A–C, 40A–C, 45A–C, 50A–C, 58A–C, 63A–C, 68A–C)	**3**
2	Head and rostrum without seta (Fig. 35A, C). Pronotum with 3 *pls* and without *ls* (Fig. 35A–C). Abdominal segments I–VI with 4 setae dorsally (Fig. 35B)	***R.rara***
–	Head with 1 *os* and rostrum with 1 *pas* (Fig. 30A, C). Pronotum with 2 *pls* and 1 *ls* (Fig. 30A–C). Abdominal segments I–VI with 3 setae dorsally (Fig. 30B)	***R.pilosa***
3	All femora with 2 setae (Figs 5A–C, 10A–C)	**4**
–	All femora with 1 seta (Figs 15A–C, 20A–C, 25A–C, 40A–C, 45A–C, 50A–C, 58A–C, 63A–C, 68A–C)	**6**
4	Rostrum with 2 *pas*. Abdominal segments I–VII with 5 setae ventrally	***R.bipustulata***
–	Rostrum without or with 1 *pas* (Figs 5A,C, 10A,C). Abdominal segments I–VII with 4 setae ventrally (Figs 5A, 10A)	**5**
5	P–pr elongated, sharply ended (Fig. 5A, C). Rostrum up to 1.60 mm ♂ and 2.60 mm ♀. Rostrum with 1 *pas*, 2 *rs*, and 1 *es* (Fig. 5A, C). Pronotum with 2 *as* and 4 *pls* (Fig. 5A–C). Procoxae with 1 seta (Fig. 5A)	***R.asellus***
–	P–pr short, flattened (Fig. 10A, C). Rostrum up to 0.70 mm ♂ and ♀. Rostrum without *pas* and *es*, with 1 *rs* (Fig. 10A, C). Pronotum with 1 *as* and 3 *pls* (Fig. 10A–C). Procoxae without seta (Fig. 10A)	***R.tetra***
6	Each of abdominal segments I–VII with 3 or more setae dorsally (Figs 15B, 20B, 40B, 45B, 50B, 58B, 63B, 68B)	**7**
–	Each of abdominal segments I–VII with 2 setae dorsally (Fig. 25B)	***R.linariae***
7	A–pr well developed, prominent, protruding past the outline of the body (Figs 15A–C, 20A–C, 40A–C)	**8**
–	A–pr vestigial (almost invisible) or absent (Figs 45A–C, 50A–C, 58A–C, 63A–C, 68A–C)	**10**
8	P–pr well developed, prominent (Figs 15A–C, 20A–C). Clubs smooth (Figs 15A–C, 20A–C)	**9**
–	P–pr vestigial (almost invisible; Fig. 40A–C). Clubs covered with knobby protuberances (Fig. 40A–C)	***R.herbarum***
9	Pronotum with 2 *as*, without *ds*, 4 *ls*, and 3 *pls* (Fig. 15A–C). Rostrum without seta (Fig. 15A, C)	***R.antirrhini***
–	Pronotum with 2 *as*, 1 *ds*, 2 *ls*, and 4 *pls* (Fig. 20A–C). Rostrum with 1 *rs* (Fig. 20A, C)	***R.florum***
10	Medial abdominal ventral seta much longer (or robust, spike-like) than the remaining ventral setae (Figs 63A, 68A)	**11**
–	All abdominal ventral setae similar in size (Figs 45A, 50A, 58A)	**12**
11	Pronotum with 2 *as*, 1 *ds*, 2 *ls*, and 4 *pls* (Fig. 63A–C). Abdominal ventral setae robust, spike-like (Fig. 63A). Abdominal segments I–VII with 6 setae dorsally (Fig. 63B)	***R.vestita***
–	Pronotum with 1 *as*, 1 *ds*, 4 *ls*, and 3 *pls* (Fig. 68A–C). Abdominal ventral setae elongated, hair-like (Fig. 68A). Abdominal segments I–VII with 3 setae dorsally (Fig. 68B)	***R.melas***
12	Abdominal segments I–VII with 4 setae dorsally (Figs 45B, 50B)	**13**
–	Abdominal segments I–VII with 5 setae dorsally (Fig. 58A)	***R.neta***
13	Rostrum with 1 *pas*, without *rs* (Fig. 45A, C). Pronotum with 3 *as*, 1 *ds*, 2 *ls*, and 2 *pls* (Fig. 45A–C)	***R.collina***
–	Rostrum with 1 *rs*, without *pas* (Fig. 50A, C). Pronotum with 3 *as*, without *ds*, 3 *ls*, and 3 *pls* (Fig. 50A–C)	***R.eversmanni***

## ﻿Discussion

### ﻿Comparison of the immature stages of *Rhinusa*

The most characteristic and commonly shared attributes among *Rhinusa* larvae are as follows: (**1**) pronotal shield is indistinct and not pigmented (only in *R.eversmanni* pigmented slightly more than the rest of the segment); (**2**) thoracic prodorsal folds are always much smaller than postdorsal folds or even vestigial; (**3**) abdominal postdorsal folds (especially of segments III–VII) higher than prodorsal folds or even in the form of conical protuberances; (**4**) cuticle covered with fine, sharp asperities; (**5**) cuticle without dark spots or dark pigmentation; (**6**) head slightly narrowed bilaterally, seldom rounded; (**7**) labrum with 2 *als* (except in *R.asellus*, *R.bipustulata* and *R.neta*, which have 3 *als*); (**8**) *des_1_* short or absent, rarely elongated; and (**9**) *fs_1-2_* usually absent or minute. Other larval characters, such as the colour of the head, or some chaetotaxy as the counts of setae on the head (*pes* and *ves*) and mouth parts (*lrs*, *mes*, and *ams*) and finally the counts of thoracic and abdominal setae, show significant interspecific variability in *Rhinusa* larvae.

The identification of attributes diagnostic of genus *Rhinusa* seems to be much easier for the pupal than for the larval stage. All described pupae present large interspecific variability both in chaetotaxy and body shape. Among all pupal characters, the most commonly shared are (**1**) head protuberances always present; (**2**) head and rostrum with very limited numbers of setae (except in *R.asellus* and *R.bipustulata*); (**3**) pronotal protuberances (if present) separated at the base of the pronotum, flattened (only in *R.asellus* conical); (**4**) abdominal protuberance on abdominal segment VIII usually visible; (**5**) femora usually with a single *fes*; and (**6**) urogomphi short or vestigial. The other characters (mainly chaetotaxy) are highly variable between species.

Based on adult morphological characters and host plants, Caldara et al. (2010) proposed the division of *Rhinusa* into several groups of species. Except for *R.rara*, all currently studied species were investigated in the species group study by Caldara et al. (2010): namely, the *R.bipustulata* group (here with only the nominotypic species); the *R.tetra* group with two species (*R.tetra*, *R.asellus*); the *R.antirrhini* group with two species (*R.antirrhini*, *R.florum*); the *R.linariae* group with the nominotypic species; the *R.herbarum* group with the nominotypic species; the *R.neta* group with four species (*R.neta*, *R.collina*, *R.eversmanni*, *R.incana*); the *R.vestita* group with the nominotypic species; and finally the *R.melas* group with the nominotypic species. Our study confirms that some specific morphological characters are uniquely characteristic of each species group according to Caldara et al. (2010) and, in particular, shows again that all species considered can be identified by examining larvae and pupae based on at least one character state. However, it is worth stressing that immatures of species that belong to a particular group have some important similarities with each other. Unique morphological characters of larvae or pupae are listed here for groups with more than one described representative:

***R.tetra* group**: larvae (epicranial line reaching 1/2 of the frons (Figs 2A, 6A); premental sclerite well sclerotised, Y-shaped (Figs 2E, 6E); posterior extension of premental sclerite present (Figs 2E, 6E); labral rods kidney-shaped (Figs 2C, 6C)) and pupae (abdominal protuberances rounded (Figs 5A–C, 10A–C); urogomphi very short (Figs 5A–C, 10A–C); each femora with two *fes* (Figs 5A,C, 10A,C))

***R.antirrhini* group**: larvae (epicranial line reaching 3/4 of the frons (Figs 12A, 17A); premental sclerite vestigial (Figs 12E, 17E); posterior extension of premental sclerite absent (Figs 12E, 17E); thoracic spiracles bicameral (Figs 13A, 18A); abdominal segments I–VII with two *eps* (Figs 13B, 18B)) and pupae (urogomphi relatively well developed (Figs 15A–C, 20A–C))

***R.neta* group**: larvae (epicranial line reaching 4/5 of the frons (Figs 42A, 47A, 52A, 55A); frons densely covered with cuticular processes (Figs 42A, 47A, 52A, 55A); premental sclerite U-shaped, posterior extension of premental sclerite absent (Figs 42E, 47E, 52E, 55E); labium usually covered with asperities (Figs 42E, 47E, 52E, 55E); labral rods kidney-shaped (Figs 42C, 47C, 52C, 55C)) and pupae (abdominal ventrites with three setae each (Figs 45A, 50A, 58A); abdominal protuberances vestigial or absent (Figs 45A–C, 50A–C, 58A–C))

Based on the following morphological similarities, *R.rara* is an undeniable member of the ***R.pilosa* group**: larvae (head wide (Figs 27A, 32A); epicranial line reaching 1/2 of the frons (Figs 27A, 32A); premental sclerite vestigial, posterior extension of premental sclerite absent (Figs 27E, 32E); clypeus and labrum fused (Figs 27C, 32C); labral rods rounded (Figs 27C, 32C); abdominal postdorsal folds in the form of conical protuberances (Figs 28B, 33B)) and pupae (pronotal and abdominal protuberances absent (Figs 30A–C, 35A–C)).

It was very challenging to identify traits distinguishing a particular species group because the remaining species groups were represented by only one species. However, it is worth stressing that immatures of species representing these species groups differ significantly from those of other species groups. As a result, the findings of the investigation regarding immature stages strongly support the taxonomic division that Caldara et al. (2010) proposed.

### ﻿Comparison of the immature stages of *Rhinusa* and *Gymnetron*

The taxonomic positions of the genera *Gymnetron* and *Rhinusa* within Mecinini are still the subject of extensive study and discussion. With regard to the characters of the immature stages, despite the predominant similarities between the two genera, there are obvious visible differences in body structures.

Specifically, in the larval stage, the most visible differences between *Gymnetron* and *Rhinusa* are as follows: (1) cuticle smooth or covered with knobby, darkly pigmented spots (vs. cuticle covered with sharp asperities, unicoloured); (2) abd. segment X exserted, well visible, setae present (vs. abd. segment X completely hidden inside the IX segment, seta mostly absent); (3) pronotal shield usually well separated, darkly pigmented (vs. pronotal sclerite usually absent); (4) thoracic prodorsal folds usually with 2 *prs* (vs. thoracic prodorsal folds usually with 1 *prs*); (5) epipharynx mostly with 3 *als* (vs. epipharynx mostly with 2 *als*); and (7) premental sclerite usually incomplete, at most in the form of a tiny ring, posterior extension always absent (vs. premental sclerite at least in the form of an incomplete ring, usually well developed with elongated posterior extension (indistinguishable only in *R.linariae*)).

In the pupal stage, *Gymnetron* differs from *Rhinusa* by the following: (1) lack of head protuberances (vs. head protuberances always present); (2) pronotal protuberances well developed, always higher than wide, spoon-like (vs. pronotal protuberances always wider than higher, flattened) (elongated, conical only in *R.asellus*); (3) femora usually with 2 *fes* (vs. femora mostly with a single *fes*); and (4) urogomphi always present, relatively elongated (vs. urogomphi variable in shape: elongated, vestigial or absent).

The structure of the cuticle (in the larval stage) being smooth or covered with knobby, darkly pigmented spots and the presence of head protuberances (in the pupal stage) can be considered apomorphies for the genus *Rhinusa*.

However, the differentiation between *Gymnetron* and *Rhinusa* might be difficult due to some very characteristic features shared by species of the two genera: frontal suture sometimes very wide, but indistinct (e.g., Fig. 1B); labrum and clypeus fused; four anal folds, anus placed ventrally; count of setae greatly reduced; and postmentum covered with asperities. Unfortunately, other features useful in species recognition (e.g., length of endocarina, shape of labral rods) are highly interspecific variable for *Gymnetron* or *Rhinusa* species.

## ﻿Conclusions

As expected based on the appearance and lifestyles of the adults, the larvae and pupae of *Gymnetron* and *Rhinusa* are more closely similar to those of *Mecinus* than to those of *Miarus* and *Cleopomiarus*. However, it is noteworthy that the morphological differences in the immature stages between *Gymnetron* + *Rhinusa* and *Mecinus*, e.g., *des_1_* usually short, minute or absent (vs. *des_1_* always elongated), *fes_2_* always present (vs. *fes_2_* usually absent), and *mes* usually lacking or single (vs. almost two *mes*), are surely more consistent than the very few distinctive characters in the adults. On this basis, we think that a phylogenetic approach for the Mecinini based on the morphological characters of the immature stages will be able to further clarify the complex systematics of these genera, and this is our intended next step.

## Supplementary Material

XML Treatment for Rhinusa

XML Treatment for Rhinusa asellus

XML Treatment for Rhinusa tetra

XML Treatment for Rhinusa antirrhini

XML Treatment for Rhinusa florum

XML Treatment for Rhinusa linariae

XML Treatment for Rhinusa pilosa

XML Treatment for Rhinusa rara

XML Treatment for Rhinusa herbarum

XML Treatment for Rhinusa collina

XML Treatment for Rhinusa eversmanni

XML Treatment for Rhinusa incana

XML Treatment for Rhinusa neta

XML Treatment for Rhinusa vestita

XML Treatment for Rhinusa melas

## ﻿Additional information

### Author ORCIDs

Rafał Gosik https://orcid.org/0000-0002-2083-4905

Roberto Caldara https://orcid.org/0000-0001-9932-7078

Ivo Toševski https://orcid.org/0000-0002-3666-3151

Jiří Skuhrovec https://orcid.org/0000-0002-7691-5990

### Data availability

All of the data that support the findings of this study are available in the main text.
